# Chemokine-like Orion is involved in the transformation of glial cells into phagocytes in different developmental neuronal remodeling paradigms

**DOI:** 10.1242/dev.201633

**Published:** 2023-10-02

**Authors:** Clarisse Perron, Pascal Carme, Arnau Llobet Rosell, Eva Minnaert, Salomé Ruiz-Demoulin, Héloïse Szczkowski, Lukas Jakob Neukomm, Jean-Maurice Dura, Ana Boulanger

**Affiliations:** ^1^ IGH, Univ Montpellier, CNRS, Montpellier, France; ^2^ Department of Fundamental Neurosciences, University of Lausanne, 1005 Lausanne, Switzerland

**Keywords:** Neuron, Glia, Axon and cell body remodeling, Phagocytosis, Chemokine-like Orion, *Drosophila*

## Abstract

During animal development, neurons often form exuberant or inappropriate axons and dendrites at early stages, followed by the refinement of neuronal circuits at late stages. Neural circuit refinement leads to the production of neuronal debris in the form of neuronal cell corpses, fragmented axons and dendrites, and pruned synapses requiring disposal. Glial cells act as predominant phagocytes during neuronal remodeling and degeneration, and crucial signaling pathways between neurons and glia are necessary for the execution of phagocytosis. Chemokine-like mushroom body neuron-secreted Orion is essential for astrocyte infiltration into the γ axon bundle leading to γ axon pruning. Here, we show a role of Orion in debris engulfment and phagocytosis in *Drosophila*. Interestingly, Orion is involved in the overall transformation of astrocytes into phagocytes. In addition, analysis of several neuronal paradigms demonstrates the role of Orion in eliminating both peptidergic vCrz^+^ and PDF-Tri neurons via additional phagocytic glial cells like cortex and/or ensheathing glia. Our results suggest that Orion is essential for phagocytic activation of astrocytes, cortex and ensheathing glia, and point to Orion as a trigger of glial infiltration, engulfment and phagocytosis.

## INTRODUCTION

During animal development, neurons often form exuberant or incorrect axons and dendrites at early stages, followed by the refinement of neuronal circuits at late stages. Neural circuit refinement can proceed by the complete elimination of a neuron and its projections or the selective destruction of specific axons, dendrites or synapses. During development, this elimination occurs by different mechanisms, such as axonal degeneration, axon retraction or cell apoptosis. Similar molecular and cellular mechanisms are at work during neurodevelopmental disorders or after nervous system injury (Llobet Rosell and Neukomm, 2019; Luo and O'Leary, 2005; Neukomm and Freeman, 2014; Riccomagno and Kolodkin, 2015; Schuldiner and Yaron, 2015).

Neural circuit refinement leads to the production of large amounts of neuronal debris in the form of neuronal cell corpses, fragmented axons and dendrites, and pruned synapses requiring disposal. In particular, the predominant phagocytes acting during neuronal remodeling and degeneration are glial cells, and crucial signaling pathways between glia and neurons leading to phagocytosis have been recently identified (Boulanger and Dura, 2022). The elimination of neuronal debris by glial cells can be divided into three different cellular steps. The first step is the infiltration of axon bundles by glial cells to reach the target to be degraded. The second is the recognition/engulfment of neuronal debris. The third step is phagocytosis through the endocytosis of the engulfed debris by glial cells resulting in the formation of phagosomes which subsequently mature.

In *Drosophila*, the mushroom body (MB), a brain memory center, is remodeled at metamorphosis, and MB γ neuron pruning occurs by a degenerative mechanism (Boulanger and Dura, 2015; Watts et al., 2003; Yaniv and Schuldiner, 2016; Yu and Schuldiner, 2014). The γ axon fragmentation is controlled by the MB intrinsic γ neuron program, which depends essentially on the ecdysone receptor, transcriptional regulation of which is finely orchestrated (Boulanger et al., 2011; Boulanger and Dura, 2015). Astrocytic glia surrounding the MB has an active role in the process; blocking their infiltration into the MBs prevents remodeling (Awasaki and Ito, 2004; Hakim et al., 2014; Tasdemir-Yilmaz and Freeman, 2014). Thus, infiltration of astrocytic processes into the MB γ axon bundle appears to be an essential first step for the elimination of these axons by glia. In the ventral nerve cord (VNC), astrocytes already infiltrate the neuropil early in development and transform into phagocytes removing excess synaptic terminals at the initiation of metamorphosis. Peptidergic VNC Corazonin-expressing neurons (vCrz) are a known example of VNC neurons, the neurites of which are eliminated by astrocytes. Interestingly, the cell bodies of these neurons were cleared by non-astrocyte glia (Tasdemir-Yilmaz and Freeman, 2014). Thus, even though astrocytes have been identified as the major phagocytic cell type responsible for engulfing degenerating axons (Doherty et al., 2009; Stork et al., 2014) other glial cell types, such as ensheathing glia, cortex glia and wrapping glia, are involved in remodeling (Bittern et al., 2021; Boulanger and Dura, 2022). These phagocytic glial cells are located in different regions of the central and peripheral nervous system. Recent studies have shown that peptide-dispersing factor tritocerebrum (PDF-Tri) neurons die by apoptosis following adult eclosion (Gatto and Broadie, 2011) and are eliminated by ensheathing and cortex glia but not by astrocytes (Vita et al., 2021). In addition, ensheathing glia has a major role in the elimination of injury-induced debris. Several examples of the ensheathing glia function on olfactory receptor neuron (ORN) debris phagocytosis after antenna or palp ablation have been reported (Doherty et al., 2009; Musashe et al., 2016). The role of wrapping glia in neuronal remodeling was poorly illustrated, even though altering wrapping glia function blocks neuromuscular junction (NMJ) remodeling during metamorphosis (Boulanger et al., 2012). Moreover, after section of the L1 wing nerve, expression of the phagocytosis receptor *draper* (*drpr*) in wrapping glia was essential for the elimination of the resulting debris, suggesting a phagocytic role of this type of glia in wing nerve after injury (Neukomm et al., 2014).

Little is known about the signaling pathways and neuron-secreted ligands that activate glia and lead to their phagocytic transformation. Some ligands like phosphatidylserine (PS), Ilps, Spz and sAppl (Boulanger and Dura, 2022) are secreted or presented by neurons to glia to activate phagocytosis pathways. We recently isolated the chemokine-like MB neuron-secreted Orion ligand by ethyl methanesulfonate (EMS) mutagenesis (Boulanger et al., 2021). Orion acts non-cell-autonomously and is essential for astrocyte infiltration into the γ axon bundle leading to γ axon pruning. Moreover, we showed that the significant amount of axonal debris seen in adult *orion* null individuals was due to the failure of astrocytes to clear debris left from axon fragmentation. Interestingly, it was recently shown that secreted Orion detects the eat-me signal PS and bridges PS and Drpr during phagocytosis of *Drosophila* dendritic arborization neurons (Ji et al., 2023). Orion bears some chemokine features, such as a CX3C motif and three glycosaminoglycan (GAG) binding consensus sequences required for its function. Chemokines are a family of chemoattractant cytokines characterized by a CC, CXC or CX3C motif promoting the directional migration of cells. Mammalian CX3CL1 (also known as fractalkine) is involved in neuron-glia communication (Arnoux and Audinat, 2015; Paolicelli et al., 2014; Wilton et al., 2019). Fractalkine and its receptor, CX3CR1, have been recently shown to be required for post-trauma cortical brain neuron microglia-mediated remodeling in a whisker lesioning paradigm (Gunner et al., 2019).

To determine whether Orion was only needed for astrocyte infiltration into axonal MB bundles or whether it could have a general function in debris engulfment and phagocytosis, we examined the elimination of synaptic debris by astrocytes in the VNC, taking advantage of the fact that astrocytes are already present in the VNC at larval stages (Stork et al., 2014) before neuronal remodeling. Interestingly, we show that Orion is involved in the overall transformation of astrocytes into phagocytes. In addition, analysis of different neuronal remodeling paradigms, including vCrz^+^ and PDF-Tri neurons, demonstrate the role of Orion in eliminating structures other than MBs via ensheathing and cortex glia. Therefore, our results indicate that Orion activates phagocytosis in three different glial cells (astrocytes, cortex and ensheathing glia) and point to Orion as an activator not only of glial infiltration but also of engulfment and phagocytosis.

## RESULTS

### Orion is required for debris engulfment and phagocytosis by astrocytes in the pupal neuropil

**Fig. 1.**
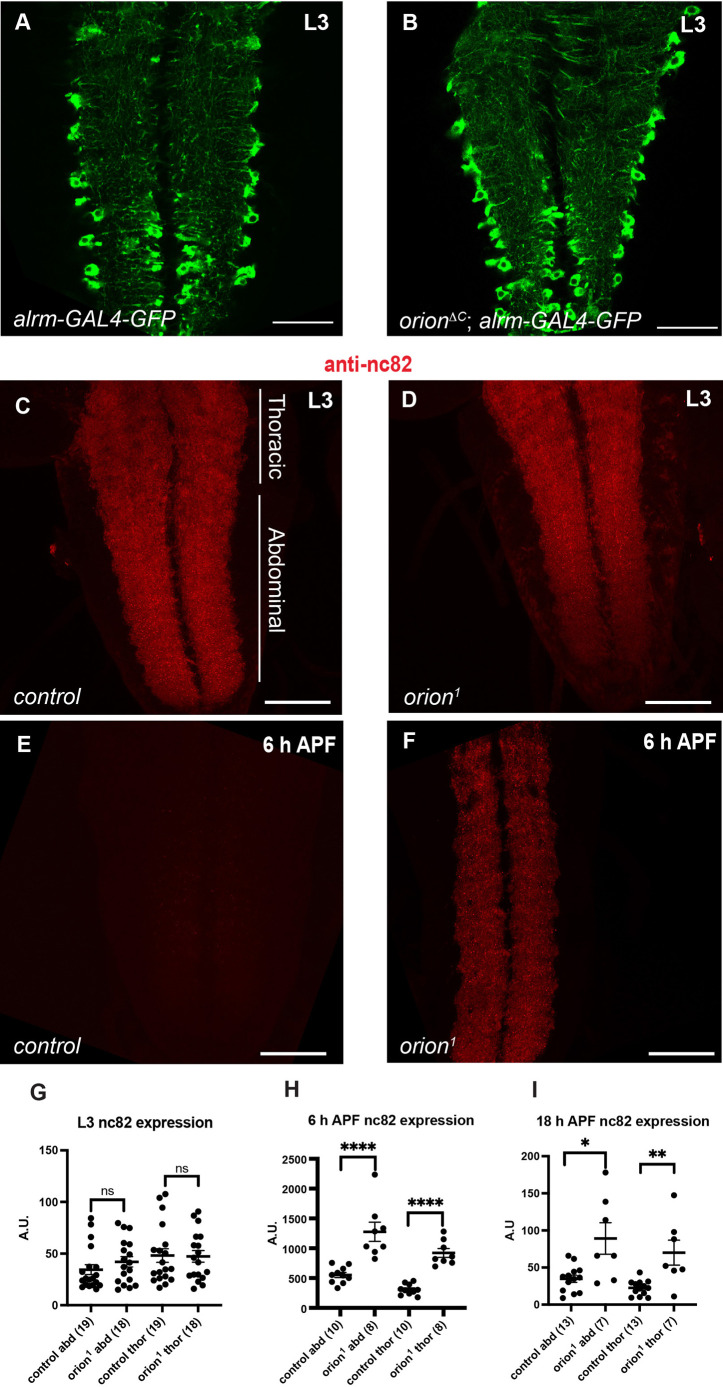
**Orion is required for the elimination of synaptic material in the astrocyte-infiltrated VNC.** (A,B) Astrocytes in controls (A) and *orion^ΔC^* (B) are visualized by the expression of *alrm-GAL4*-driven *UAS-mCD8-GFP* (green) at larval stage (*n*≥20 for each condition). Note that astrocyte extensions infiltrate the larval neuropil in both conditions. (C-F) Active zones were labeled with the nc82 antibody (antibody for Brp, red) at larval (C,D) and 6 h APF (E,F) stages in control (C,E) and *orion^1^* mutants (D,F) (*n*≥20 for each condition). Note the higher amount of nc82 staining observed at 6 h APF in F compared with wild type in E. Confocal images are *z*-projections. (G-I) Quantifications of expression levels of nc82^+^ puncta in arbitrary units at L3 (G), 6 h APF (H) and 18 h APF (I) in thoracic (thor) and abdominal (abd) regions (shown in C). Genotypes are listed in supplementary Materials and Methods, List of fly strains. Results are mean±s.e.m. *n* values are indicated in a parenthesis for each condition. Replicated three times. *P<0.05, **P<0.01, ****P<0.0001 (Mann–Whitney *U*-test). ns, non-significant. See Table S1 for raw data. Scale bars: 40 µm in A,B; 50 µm in C-F.

We recently established that Orion is required for astrocyte infiltration into the MB γ axon bundle during γ axon pruning (Boulanger et al., 2021). Consequently, the phagocytic steps succeeding infiltration, such as debris engulfment, phagosome formation and debris digestion into astrocytes, do not occur in the absence of Orion. As astrocytes send extensions and already infiltrate the whole neuropil during larval stages before remodeling at pupal stages, we considered the VNC a suitable emplacement to determine whether Orion was involved in debris engulfment and phagosome formation. For this purpose, we first examined the overall morphology of the VNC-located astrocytes in wild type and *orion* mutants at the L3 stage using *UAS-CD8-GFP* driven by the astrocyte-specific *alrm-GAL4* (*n*≥20) (Fig. 1A,B). In both cases, astrocytes displayed similar morphologies and locations, with thin extensions infiltrating the inner side of the neuropil. Similar results were observed with both *orion^1^* and *orion^ΔC^* mutant alleles and we used either one of those alleles during this work.

**Fig. 2.**
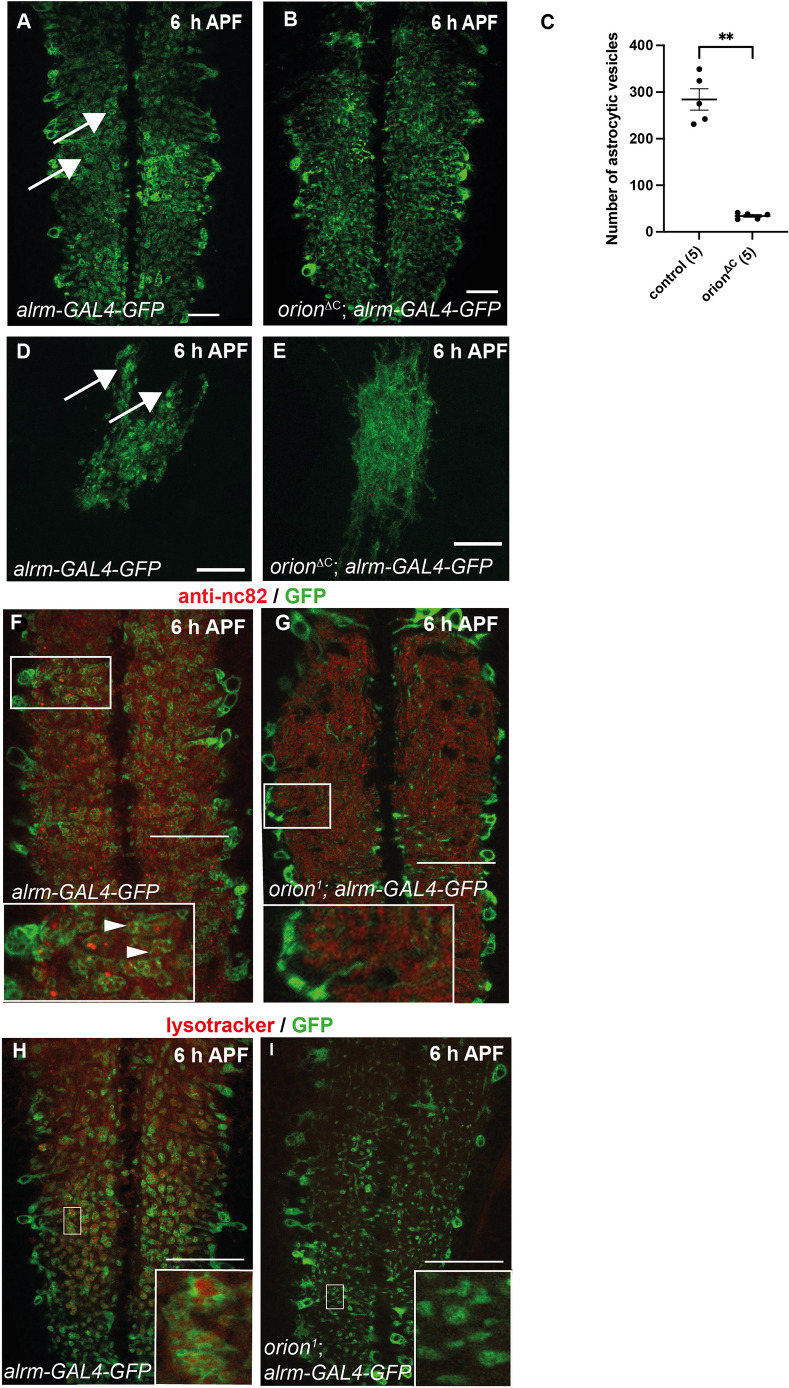
**Orion regulates the transformation of astrocytes into phagocytes that engulf and phagocytose synaptic material in the pupal neuropil.** (A-I) Astrocytes are visualized by the expression of *alrm-GAL4*-driven *UAS-mCD8-GFP* (green) in VNCs (A,B,F-I) and astrocyte clones (D,E). Red staining represents either active zones labeled with the nc82 antibody (F,G) or phagosomes labeled with a lysotracker staining (H,I). GFP-labeled vesicular structures are shown by arrows in A,D. Vesicles are mostly not observed, or are very small in *orion* mutants (B,E). *n*≥20 VNC and *n*≥10 clones in controls and *orion* mutants. (C) Quantification of the number of astrocytic vesicles in control and *orion* mutant whole VNC. The number of VNC analyzed is included in a parenthesis for each condition. Results are mean±s.e.m. **P<0.01 (Mann–Whitney *U*-test). Insets in F show astrocytic vesicles containing engulfed synaptic debris (arrowheads), absent in *orion* mutants (G). Inset in H shows astrocytic vesicles containing acidic phagosomes labeled with lysotracker. Note that lysotracker staining is not observed in *orion^1^* vesicles (I). Confocal images are *z*-projections. Genotypes are listed in supplementary Materials and Methods, List of fly strains. Replicated three times. See Table S2 for raw data. Scale bars: 30 µm in A,B; 20 µm in D,E; 40 µm in F-I.

Neuronal remodeling during metamorphosis results in the loss of nearly all synapses in the neuropil by 48 h after pupa formation (APF), before adult-specific synapses are generated. To determine whether Orion has an additional role in astrocyte engulfment and phagocytosis, we used antibodies against the presynaptic active zone marker Bruchpilot (Brp: also known as Nc82) to label synapses and examine their fate at larval and pupal stages. At L3, Nc82 labeled abdominal (abd) and thoracic (thor) VNC synapses throughout the neuropil at similar intensities in controls and null allele *orion^1^* (Fig. 1C,D,G). At 6 h APF, Nc82 staining persisted in *orion* mutant VNC compared with controls (Fig. 1E,F,H), as well as at 18 h APF (Fig. 1I). However, at this late stage, some VNC showed wild-type Nc82 staining, suggesting a compensatory pruning event. Together these data point to an additional role of Orion in the engulfment and phagocytosis of VNC synapses by the surrounding astrocytic cells. To confirm this data, we examined the astrocyte process morphology at 6 h APF in wild type and *orion*-lacking flies. In wild type, VNC astrocytes displayed vacuolar structures (Tasdemir-Yilmaz and Freeman, 2014) compared with null allele *orion^ΔC^*, in which the astrocyte structure was filamentous all over the VNC with a low number of small vesicles (Fig. 2A-C). Suppression of astrocytic transformation was also observed in *orion* mutant astrocyte clones, visualized in homozygous mutant animals, labeled with *mCD8-GFP* driven by *alrm-GAL4*, in which vesicular phagocytic structures were not apparent compared with wild-type astrocyte clones (Fig. 2D,E). Next, we sought to determine whether Orion was involved in astrocytic synapse engulfment. We observed GFP^+^ astrocyte extensions in wild-type and *orion* mutant VNCs (*n*≥7 VNCs), but engulfed synaptic debris (Nc82-labeled particles delimited by GFP^+^ rings contained in astrocytic processes) was only observed in wild-type conditions (Fig. 2F,G). Finally, to determine whether these vesicles corresponded to phagocytic vesicles, we labeled VNCs with the phagolysosome marker lysotracker. Acidic phagocytic activity was only observed in controls at 6 h APF, reflected by a high lysotracker staining inside astrocytic vesicles, which was absent in the rare small astrocytic vesicles observed in *orion-*lacking VNCs (Fig. 2H,I). These results suggested that Orion, in addition to its role in astrocytic infiltration into the MBs, has additional functions in synapse engulfment and phagocytosis, and point to Orion as an overall activator of astrocyte transformation into phagocytes.

### Orion is required for cortex glia-mediated phagocytosis of the vCrz neuron cell body in the pupal neuropil

**Fig. 3.**
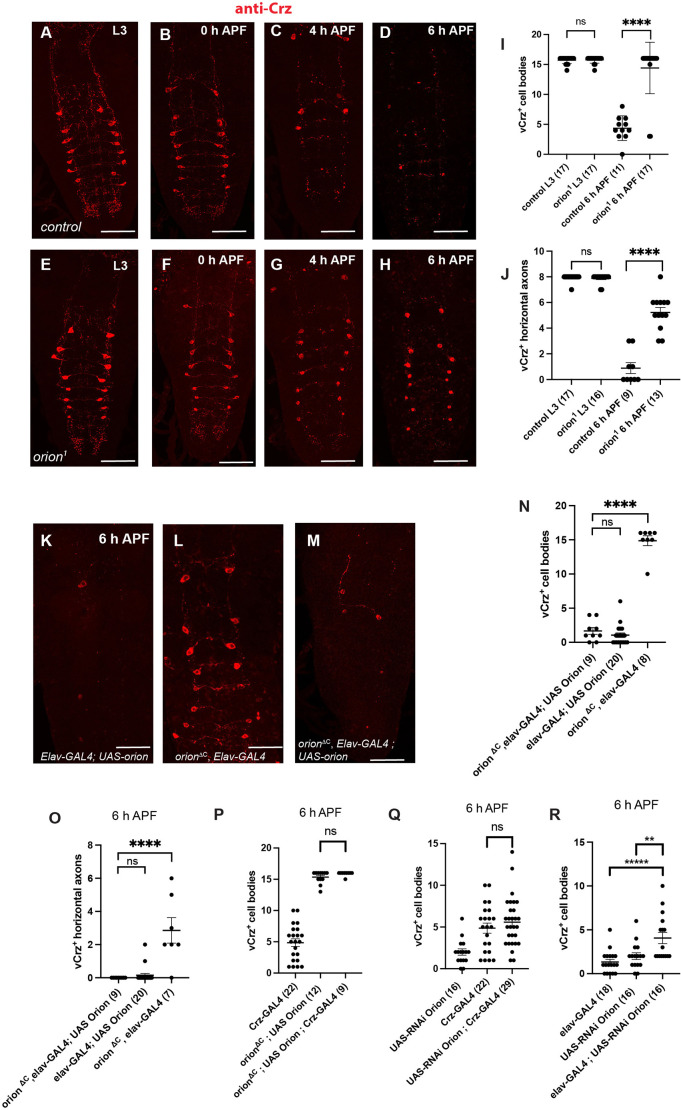
**Orion is required for the elimination of vCrz^+^ cell bodies and neurites.** (A-J) vCrz^+^ neurons were labeled with an anti-Crz antibody (red) at the indicated time points at larval (L3) and pupal stages in controls (A-D) and *orion^1^* mutants (E-H). (I) Quantification of vCrz^+^ cell bodies. (J) Quantification of vCrz^+^ horizontal axons. (K-M) 6 h APF vCrz^+^ neurons were labeled with anti-Crz (red) in controls (K) and *orion* mutants (L). Neuronal expression of *orion* rescued the *orion^ΔC^* phenotype (M). (N-R) Quantification of vCrz^+^ cell bodies (N,P-R) and horizontal axons (O) in rescue experiments using *UAS-orion* driven by *elav-GAL4* (N,O) or *Crz-GAL4* (P) and quantification of vCrz^+^ cell bodies in experiments using *UAS-Orion-RNAi* driven by *Crz-GAL4* (Q) and *elav-GAL4* (R). Genotypes are listed in supplementary Materials and Methods, List of fly strains. Error bars represent mean±s.e.m. *n* values are indicated in a parenthesis for each condition. **P<0.01, ****P<0.0001 (Mann–Whitney *U*-test). ns, non-significant. Replicated at least twice. See Table S3 for raw data. Scale bars: 70 µm in A-H; 40 µm in K-M.

As Orion is involved in eliminating synaptic debris in the neuropil, we raised the question of whether its action in the neuropil is specific to astrocyte glia or whether other types of phagocytic glial cells could also be targets of Orion. To explore this possibility, we examined the peptidergic vCrz^+^ neurons that undergo apoptosis during early metamorphosis between 0 and 6 h APF, the cell bodies and neurites of which, projecting into the neuropil, are eliminated (Choi et al., 2006). The vCrz^+^ system is composed of eight pairs of neurons (16 cell bodies) and three different types of neurite tracks: eight horizontal neurites connecting cell bodies from the same segment, two medial vertical neurite tracks in the center of the neuropil and two lateral neurite tracks vertically connecting cell bodies from contiguous hemi segments. Interestingly, the vCrz^+^ neurites are eliminated by astrocytic glia, but their cell body appears to be removed by other types of glia, most probably cortex glia (Tasdemir-Yilmaz and Freeman, 2014). Thus, we considered these neurons as a valuable model to determine whether Orion is involved in the elimination of cell bodies by glial cells other than astrocytes. In controls, at L3, the anti-Crz antibody recognizing the Crz neuropeptide labeled eight pairs of vCrz^+^ neurons in the VNC (and a few neurons in the brain). By 6 h APF, almost all of the vCrz^+^ neurons were cleared from the neuropil (Choi et al., 2006). In VNCs lacking *orion* (*orion^1^* and *orion^ΔC^*) we found no differences in the anti-Crz staining at L3 stages compared with wild-type larvae. However, most of the cell bodies and neurites persisted at 4 h APF in *orion* mutants, which was not the case in controls. Furthermore, many vCrz^+^ cell bodies and a high amount of neurite debris remained at 6 h APF in *orion* mutants (Fig. 3A-J). These results were confirmed by driving GFP in vCrz^+^ neuronal membranes by a Crz neuron-specific GAL4 (Fig. S1; see Table S8 for raw data). We observed that neurite and cell body membrane GFP-labeled debris remained until 18 h APF in VNCs lacking *orion* (Fig. S1E,J). However, at this late stage most of the vCrz GFP^+^ processes were missing and the persistent nuclei were small and displayed apoptotic structure with condensed GFP, suggesting a compensatory pruning event. Forced expression of *orion* in neurons using the pan-neuronal *elav-GAL4* driver at 6 h APF rescued the *orion* mutant cell body and neurite phenotype (Fig. 3K-O), indicating that expression of *orion* in all neurons is sufficient for vCrz^+^ neuron remodeling. However, expression of *orion* specifically in Crz neurons does not rescue pruning (Fig. 3P) and expression of an *UAS-Orion-RNAi* in these neurons does not lead to remodeling defects (Fig. 3Q), suggesting neuronal sources of Orion different from the Crz neurons. This idea is reenforced by the fact that an overall expression of *UAS-Orion-RNAi* in neurons driven by *elav-GAL4* results in a statistically significant decrease of vCrz neuron pruning (Fig. 3R).

**Fig. 4.**
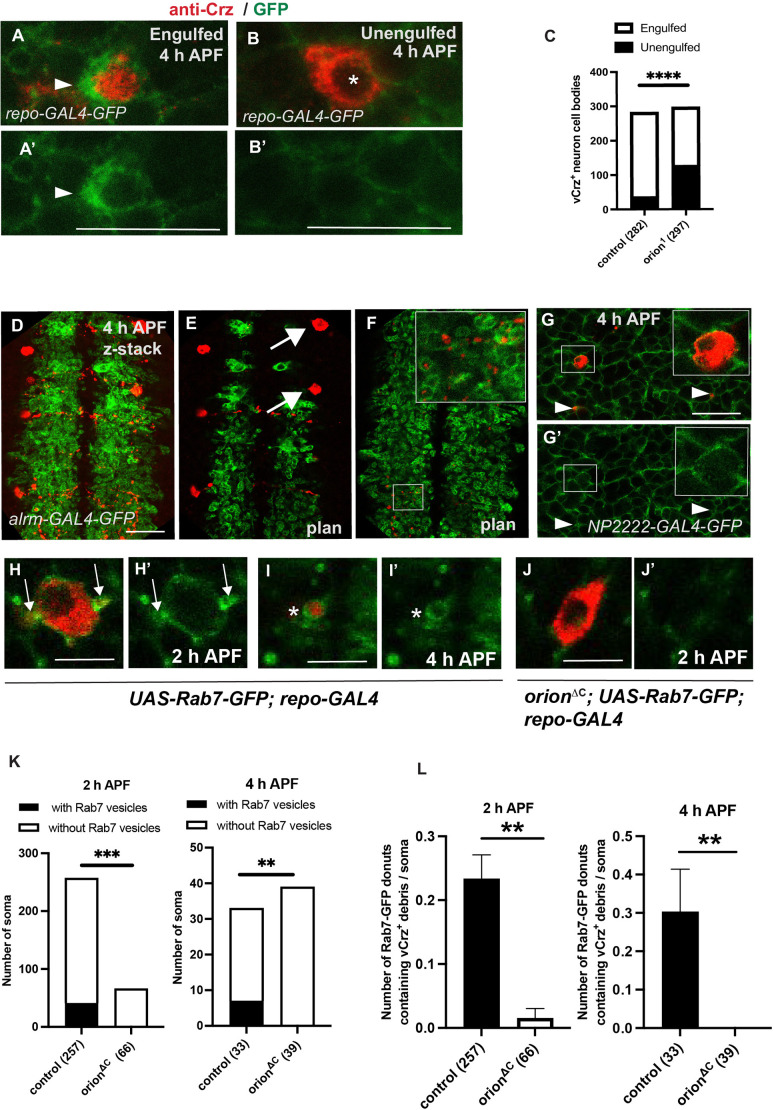
**The elimination of vCrz^+^ neurites and cell bodies is mediated by astrocytes and cortex glia respectively.** (A,B) Confocal *z*-stacks (from five to seven plans) showing expression of *UAS-mCD8-GFP* under the control of *repo-GAL4* (green) and anti-Crz staining (red) in 4 h APF neurons. Apoptotic cell body enwrapped in extended GFP^+^ cortex glia forming a phagocytic cup is shown (arrowheads) in A. A′ shows a single channel of A. Unengulfed cell body (B) displays discernible and uncondensed nuclear morphology (asterisk). GFP^+^ cortex glia still reaches the cell body but with very thin processes (B′, single channel of B). (C) Quantification of vCrz^+^ cell bodies engulfed and unengulfed by cortex glia in control versus *orion* mutant is shown. *n* values are indicated in a parenthesis for each condition. (D-F) Astrocytic glia visualized by the expression of *alrm-GAL4*-driven *UAS-mCD8-GFP* (green) and vCrz^+^ neurons by an anti-Crz antibody (red) at 4 h APF. (E) Arrows point to vCrz^+^ cell bodies which are not reached by astrocytic extensions. (F) Astrocyte extensions engulf vCrz^+^ debris. See inset for higher magnification. D is a *z*-projection confocal image, E and F are single confocal plans from D. (G,G′) Confocal plans showing cortex glia visualized by the expression of *NP2222-GAL4*-driven *UAS-mCD8-GFP* (green) and vCrz^+^ neurons by an anti-Crz antibody (red) at 4 h APF. Insets illustrate a vCrz^+^ cell body reached by cortex glia. Engulfed debris are labeled by arrowheads. *n*>5. (H-J′) are confocal plans showing *UAS-Rab7-GFP* driven by *repo-*GAL4 (green) and vCrz^+^ neurons labeled by an anti-Crz antibody (red) at 2 h and 4 h APF in wild type (H,I) and *orion* mutants (J). Rab7-GFP donuts attached to the soma contain vCrz^+^ soma-derived debris and are indicated by an arrow in H and H′. Individual donuts containing Crz^+^ debris are labeled by an asterisk in I,I′. Note the absence of Rab7 vesicles in *orion* mutants (J). H′,I′ and J′ show a single channel of H, I and J, respectively. (K,L) Quantification of the number of somas with or without Rab7 vesicles (K) and quantification of Rab7-GFP donuts containing vCrz^+^ debris per soma (L) at 2 h and 4 h APF. **P<0.01, ***P<0.001; ****P<0.0001 (Chi-square test). *n* values are indicated in a parenthesis for each condition and represent a number of soma (see raw data for number of animals). Replicated at least twice. Genotypes are listed in supplementary Materials and Methods, List of fly strains. See Table S4 for raw data. Scale bars: 20 µm in A,B; 30 µm in D-G; 10 µm in H-J.

To determine whether neuronal Orion is activating vCrz^+^ surrounding glia, leading to cell body and neurite glia-mediated phagocytosis, we analyzed the morphology of glial cells located in the vicinity of the vCrz^+^ cell bodies using the pan-glial *repo-GAL4* driver. A significantly higher number of vCrz^+^ cell bodies were engulfed (surrounded by expanded glial membranes forming phagocytic cups) by glial cells in wild type compared with *orion^1^* at 4 h APF [246/282 (87%) and 170/297 (57%), respectively] (Fig. 4A-C). Interestingly, in *orion* mutants, 42% of cell bodies were not engulfed by glia (Fig. 4A-C). Moreover, the cortex glia surrounding vCrz^+^ somas at larval stage displayed a similar morphology in controls and mutants, thus excluding a potential glial developmental defect induced by the *orion* mutation (Fig. S2). These results suggest that neuron-secreted Orion is required for glial cells to engulf vCrz^+^ cell bodies and neurites. We next sought to determine which type of phagocytic glia is involved in the elimination of vCrz^+^ cell bodies and neurites. For this purpose, we used several *GAL4* lines: *alrm-GAL4*, *NP2222-GAL4* and *MZ0709-GAL4*, specific for astrocytes, cortex glia and ensheathing glia, respectively, and analyzed *UAS-GFP*-driven expression at 4 h APF. As expected for *alrm-GAL4*, astrocyte extensions infiltrating the neuropil were covered by vesicular structures containing vCrz^+^ debris. These astrocytic extensions did not contact vCrz^+^ cell bodies (Fig. 4D-F), suggesting that astrocytes are not required for the elimination of vCrz^+^ cell bodies. We then assayed cortex glia-specific staining and observed that cortex glia extensions contact and engulf vCrz^+^ cell body debris (Fig. 4G). Moreover, we used a GFP-tagged *Rab7* construct (*UAS-Rab7-GFP* driven by *repo-GAL4*) to visualize phagocytic vesicles in cortex glia. Indeed, enhanced expression/localization of Rab7 during corpse engulfment (leading to Rab7 donut-shaped structures around apoptotic debris) has already been described during VNC remodeling as a sign of late phagosome/lysosome presence during cortex glia-mediated phagocytosis (McLaughlin et al., 2019). In controls we observed both diffuse cytoplasmic Rab7 expression and pronounced Rab7 localization in distinct donut-shaped structures. These donuts are located within cortex glial cells (identified based on their localization) that surround Crz^+^ soma-derived particles in the VNC and therefore represent a consistent argument in favor of the function of Orion in mediating cortex glia phagocytosis activity during remodeling. The impaired accumulation of Rab7 machinery in *orion* mutant backgrounds supports the idea that phagocytosis in cortex glia is defective (Fig. 4H-L). Moreover, ensheathing glia extensions were observed neither around the vCrz^+^ cell bodies nor the vCrz^+^ neurites (Fig. S3). These results indicate that Orion allows communication between neurites and astrocytes and between neuron cell bodies and cortex glia. This interaction is likely necessary to enable the elimination of cell bodies after apoptotic cell death induction.

To determine whether cortex glia have an active role in the elimination of the vCrz^+^ cell bodies during vCrz^+^ apoptosis, we compared wild-type and *orion*-lacking VNCs expressing the GFP-based caspase sensor (*UAS-Casor*) probe in Crz neurons. This probe is designed to change its subcellular localization from the cell membrane to the nucleus following proteolytic cleavage by active caspases. It allows differences in caspase activity in neurons, such as the vCrz^+^ (Lee et al., 2018), to be monitored. If Orion facilitated apoptosis after caspase activation, we would anticipate a delay in the nuclear localization of the Casor probe in *orion*-lacking VNCs compared with wild-type VNCs during development. However, no difference was observed between wild-type and *orion*-lacking VNCs (Fig. S4; see Table S9 for raw data): not at 0 h APF, at which time the overall vCrz^+^ soma GFP^+^ staining was cytoplasmic (78% and 86% for controls and *orion* mutants, respectively), nor at 2 h APF, when the cords displayed a mostly nuclear GFP^+^ staining (90% and 100% for wild type and *orion* mutants, respectively). These data suggest that Orion does not act on the vCrz^+^ neuron cell autonomous caspase activity leading to vCrz^+^ neuronal cell body apoptosis.

### Orion is required for the elimination of PDF-Tri neurons by ensheathing and cortex glia in young adult flies

**Fig. 5.**
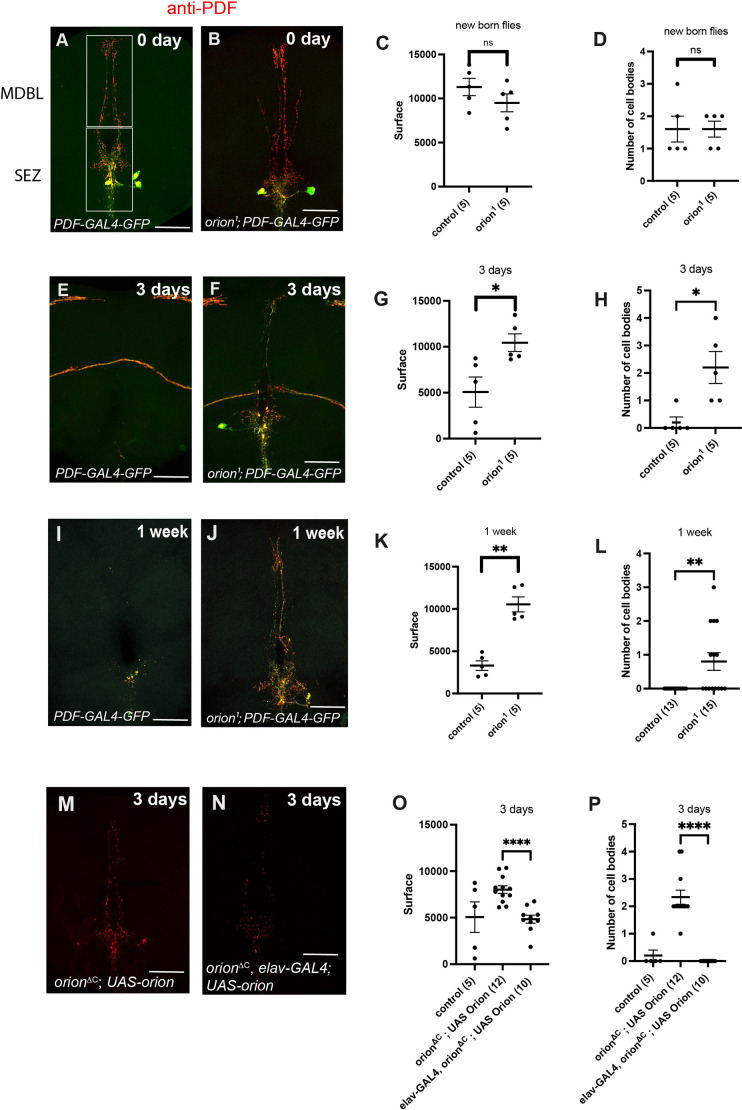
**Orion mutants retain developmentally transient PDF-Tri neurons.** (A,B,E,F,I,J) Confocal *z*-stacks showing PDF-Tri neurons visualized by the expression of *PDF-GAL4*-driven *UAS-mCD8-GFP* (green) and labeled with anti-PDF antibody (red) at the indicated time points in controls (A,E,I) and *orion^1^* mutants (B,F,J). (C,G,K) Surface (in µm^2^) occupied by the PDF-Tri arborization at 0 days (new born flies), 3 days and 1 week, respectively, in controls and *orion* mutants. (D,H,L) PDF-Tri cell bodies at 0 day, 3 days and 1 week in controls and *orion* mutants. (M,N) Confocal *z*-stacks showing PDF-Tri neurons labeled with an anti-PDF antibody at 3 days after birth in *orion^ΔC^* mutants (M) and in *orion^ΔC^* rescued by the expression of *elav-GAL4*-driven *UAS-orion* (N). (O,P) Rescue quantifications of PDF-Tri surface (in µm^2^) (O) and number of cell bodies (P) are shown. Genotypes are listed in supplementary Materials and Methods, List of fly strains. Error bars represent mean±s.e.m. *P<0.05, **P<0.01, ****P<0.0001 (Mann–Whitney *U*-test). ns, non-significant. *n* values are indicated in a parenthesis for each condition. Replicated at least twice. See Table S5 for raw data. Scale bars: 50 µm.

We used a different paradigm to study ensheathing glia phagocytic potential effects during remodeling. PDF-Tri neurons are characterized as a developmentally transient population that initially appears during mid-pupal development and undergoes programmed cell death soon after adult eclosion (Gatto and Broadie, 2011; Helfrich-Forster, 1997). PDF-Tri neurons consist of 1-2 pairs of somata bilaterally positioned in the tritocerebrum on the anterior face of the brain, close to the esophagus (Vita et al., 2021). PDF-Tri processes extend posteriorly and ventrally into the suboesophageal zone (SEZ), dorsally through the distal medial bundle (MDBL) and into the superior medial protocerebrum near the dorsal brain surface (Fig. 5A). The highly ramified dendritic arbors and axonal projections of the PDF-Tri neurons project throughout the SEZ immediately dorsal to, and surrounding, the esophageal foramen (Fig. 5A). As PDF-Tri processes project toward the posterior surface through the MDBL, two linearized branching tracks extend laterally toward the dorsal side of the brain (Fig. 5A). Recent studies have shown that these neurons fail to be removed when glial phagocytosis is blocked (Vita et al., 2021). In addition, cortex and ensheathing glia are the primary phagocytic glial cells responsible for eliminating their cell bodies and axonal processes, respectively. Consequently, the PDF-Tri neurons represent a suitable structure to establish whether Orion has a role in cell body and neurite elimination via the surrounding cortex and ensheathing glia, respectively. To examine PDF-Tri neurons, neurons were co-labeled with *PDF*-*GAL4*-driven membrane-tethered GFP and an anti-PDF (Fig. 5), which label not only the PDF-Tri neurons but also a network of surrounding PDF^+^ neurons (Fig. S5A,B; see Table S10 for raw data). In newly eclosed adults (0 days), PDF-Tri neurons are consistently present along the midline in wild-type and *orion^1^* mutant brains. Dense PDF^+^ projections occur in the SEZ and surrounding the esophageal foramen. Distribution of fluorescence and PDF-Tri staining overall are also similar in control and *orion^1^* mutants. In addition, a similar number of cell bodies is observed (Fig. 5A-D). Three days later (3 days post-eclosion), extensive removal of the PDF-Tri circuitry is underway, with the disassembly and loss of axons, dendrites and cell bodies in control animals as reported previously (Gatto and Broadie, 2011). However, the neurite circuitry and cell bodies are maintained in *orion^1^* mutants (Fig. 5E-H). Surrounding PDF^+^ neuronal circuitry, which is not fated to remodel, is affected neither in control nor mutant animals (Fig. S5A,B). The *PDF*-*GAL4*-driven GFP and anti-PDF labeling of the PDF-Tri neurons disappear with a similar time course, indicating elimination of both the developmentally transient PDF-Tri neuronal membranes and the cytoplasmic PDF peptide content at this stage. By 1 week post-eclosion (Fig. 5I-L), PDF-Tri cell bodies and most of the axonal and dendritic processes are still present in *orion^1^* mutants compared with controls, and they mainly occupy the initial surface. Finally, we observed that PDF-Tri neurites persist in *orion-*lacking flies at 3 weeks after eclosion (Fig. S5C-E), suggesting no compensatory PDF-Tri neuron pruning event in adult brains.

Forced expression of *orion* in neurons using the *elav-GAL4* driver 3 days after birth rescued the *orion* mutant cell body and neurite phenotypes (Fig. 5M-P), suggesting that expression of *orion* in neurons is sufficient for PDF-Tri neuronal remodeling. However, forced expression of *orion* in PDF-Tri neurons, using the *PDF-GAL4* driver, does not rescue these phenotypes (Fig. S5F). Moreover, expression of a *UAS-Orion-RNAi* in these neurons (*PDF-GAL4*) or in all neurons (*elav-GAL4*) does not give remodeling phenotypes when compared with controls (Fig. S5G-H), suggesting external sources of Orion.

To bolster the idea that Orion signals to glia to initiate phagocytosis for PDF-Tri neuron elimination, wild-type and *orion^1^* animals were imaged using specific glial markers and the anti-PDF antibody at 0 and 2 days post-eclosion, when cell bodies (0 day) and neurites (2 days) are still present. Cortex and ensheathing glia are both primary phagocytes of PDF-Tri neurons (Vita et al., 2021). The role of astrocytic glia was not evidenced in PDF-Tri phagocytosis, but as Orion was shown to signal to astrocytes in the MBs and the VNC, we also included them in our analysis. Selective drivers were used for the astrocyte-like (*alrm*-*GAL4*), ensheathing (*MZ0709*-*GAL4*) and cortex (*NP2222*-*GAL4*) glia. Combining *mCD8-GFP* under the control of each driver generated distinctive brain localization and cellular morphology for each glial class in regions surrounding the PDF-Tri neurons. GFP-labeled astrocytes exhibited a small cytoplasm size and some long and ramified extensions (Fig. S6). Astrocytes did not surround somas (Fig. S6A,B, arrow) or processes (Fig. S6A,C,D), and their cytoplasm did not show phagocytic vesicles. In addition, engulfment of debris was not observed at either 0 or 2 days. Astrocytic extensions became longer at 2 days, but they did not appear to phagocytose PDF-Tri debris (Fig. S6E,F′).

**Fig. 6.**
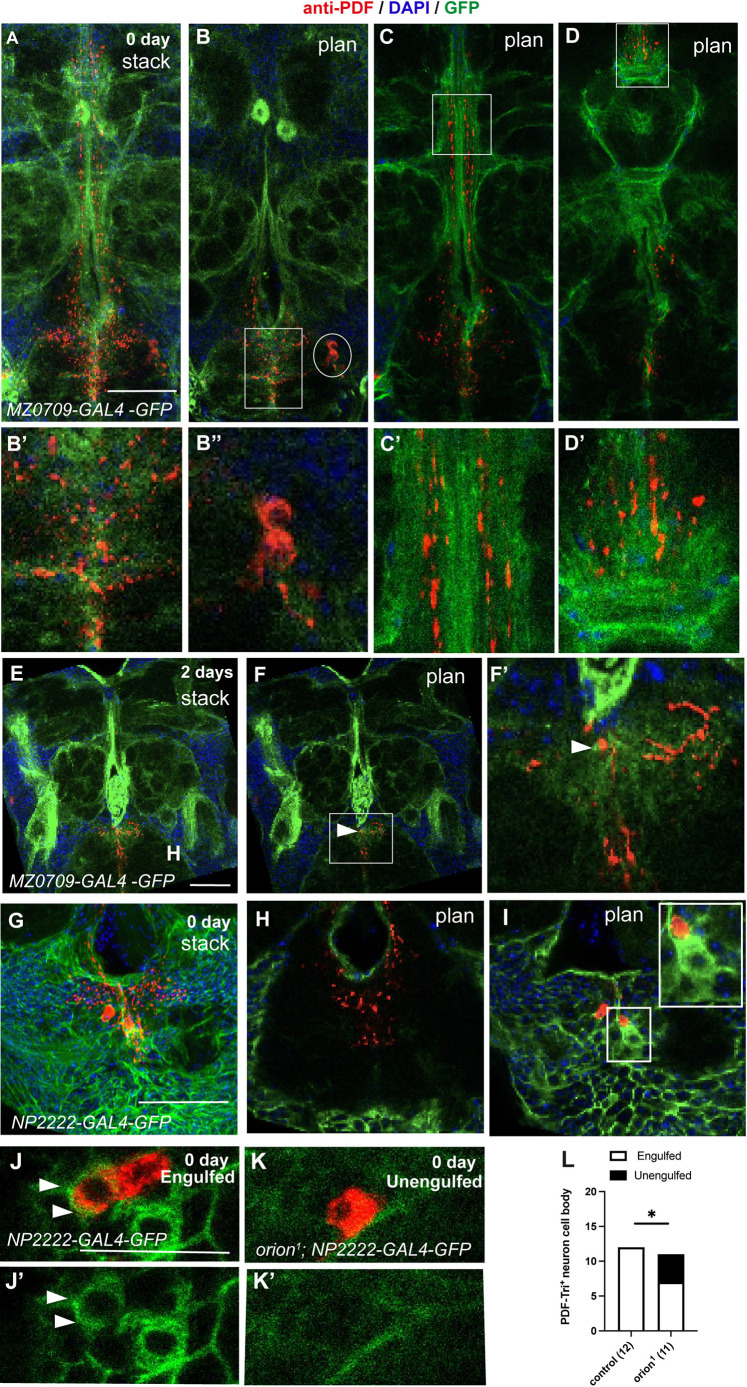
**Orion mediates PDF-Tri neuron elimination via cortex and ensheathing glia.** (A-F′) Confocal *z*-stacks or plans (labeled stack or plan) showing ensheathing glia visualized by the expression of *MZ0709-GAL4*-driven *UAS-mCD8-GFP* (green) and PDF-Tri neurons labeled with anti-PDF antibody (red) and DAPI (blue) at 0 (A) and 2 (E) days. Different regions of the brain contained in these *z*-stacks are shown as single confocal plans (B-D′ for *z*-stack in A, F,F′ for *z*-stack in E). Suboesophageal zone (SEZ) region is included in a rectangle in B and F, distal medial bundle (MDBL) regions are included in a rectangle in C and D. These regions are shown at higher magnification in B′,C′,D′ and F′, respectively, showing PDF-Tri neurites engulfed by ensheathing glia. Arrowheads point to ensheathing glia-engulfed debris in F and F′. Oval in B encircles a PDF-Tri cell body devoid of ensheathing glia, which does not engulf PDF-Tri cell bodies (higher magnification is in B″). (G) Confocal *z*-stack labeled with anti-PDF antibody (red) and DAPI (blue) and showing cortex glia visualized by the expression of *NP2222-GAL4*-driven *UAS-mCD8-GFP* (green) at 0 day. (H,I) Single confocal plans showing PDF-Tri dendrites (H) and cell bodies (I) contained in the panel G image stack. Note the absence of PDF-Tri dendrite surrounded by cortex glia in H and the high amount of cortex glia extensions present around the PDF-Tri cell bodies in I. A cortex glia cell phagocytosing a PDF-Tri cell body (red) is included in the region enclosed by the square in I. *n*≥5 for each condition. Replicated twice. (J-K′) Confocal plans showing expression of *UAS-mCD8-GFP* under the control of *NP2222-GAL4* (green) and anti-PDF staining (red) in day 0 neurons. An engulfed apoptotic PDF-Tri cell body enwrapped in thick GFP^+^ cortex glia extensions is indicated with arrowheads in J and J′. An unengulfed PDF-Tri cell body is shown in K. J′ and K′ show a single channel of J and K, respectively. (L) Quantification of PDF-Tri^+^ cell bodies engulfed and unengulfed by cortex glia is shown. *n* values are indicated in a parenthesis for each condition. Statistically significant differences were observed between the two groups (*P<0.05, Fisher test). Genotypes are listed in supplementary Materials and Methods, List of fly strains. See Table S6 for raw data. Scale bars: 50 µm in A-I; 20 µm in J,K.

Next, we looked at ensheathing glia GFP^+^-driven membranes that prominently define and surround each brain neuropil at 0 and 2 days (Fig. 6A-F′). PDF-Tri dendrites are surrounded by GFP^+^ ensheathing glial membranes at 0 day (birth) (Fig. 6A,B) as are both of the PDF-Tri vertical tracks (Fig. 6A,C) and the distal PDF-Tri neurites (Fig. 6A,D). Ensheathing glia also enwrap *orion* mutant PDF-Tri tracks (Fig. S7), which prevents us from quantifying engulfment levels. Ensheathing glia extensions are not observed around PDF-Tri cell bodies (Fig. 6B). Similar results were observed in brains at 2 days post-eclosion (Fig. 6E-F′).

We observed cortex glia extensions phagocytosing individual PDF-Tri cell bodies (Fig. 6G,I), but not neurites (Fig. 6H), at 0 day after eclosion, suggesting that Orion mediates PDF-Tri cell body phagocytosis. Control PDF-Tri cell bodies were all (100%) engulfed by cortex glia compared with *orion^1^*, of which 64% were engulfed and 36% unengulfed (Fig. 6J-L). Moreover, the cortex glia surrounding PDF-Tri somas at pharate stage displayed a similar morphology in controls and mutants, thus excluding a potential glial developmental defect induced by the *orion* mutation (Fig. S8).

As well as the vCrz^+^ cell bodies, we did not observe any active role of glia in the PDF-Tri cell body apoptosis (Fig. S9; see Table S11 for raw data). These data suggest that Orion signals to both ensheathing and cortex glia, allowing PDF-Tri neuronal debris phagocytosis, with the cortex glia acting specifically on cell body clearance and the ensheathing glia facilitating debris elimination more distally.

### Orion is dispensable for debris clearance of both ORN axons and L1 vein nerve by ensheathing and wrapping glia, respectively, after injury

A well-established system to study communication between remodeling neurons and ensheathing glia is based on the analysis of ORNs and surrounding ensheathing glia after the ablation of maxillary palps or antennae (Doherty et al., 2009). ORN cell bodies are housed in the third antennal segment and maxillary palps of adult *Drosophila*, with axons projecting to the antennal lobe of the brain via the antennal and maxillary nerve, respectively.

**Fig. 7.**
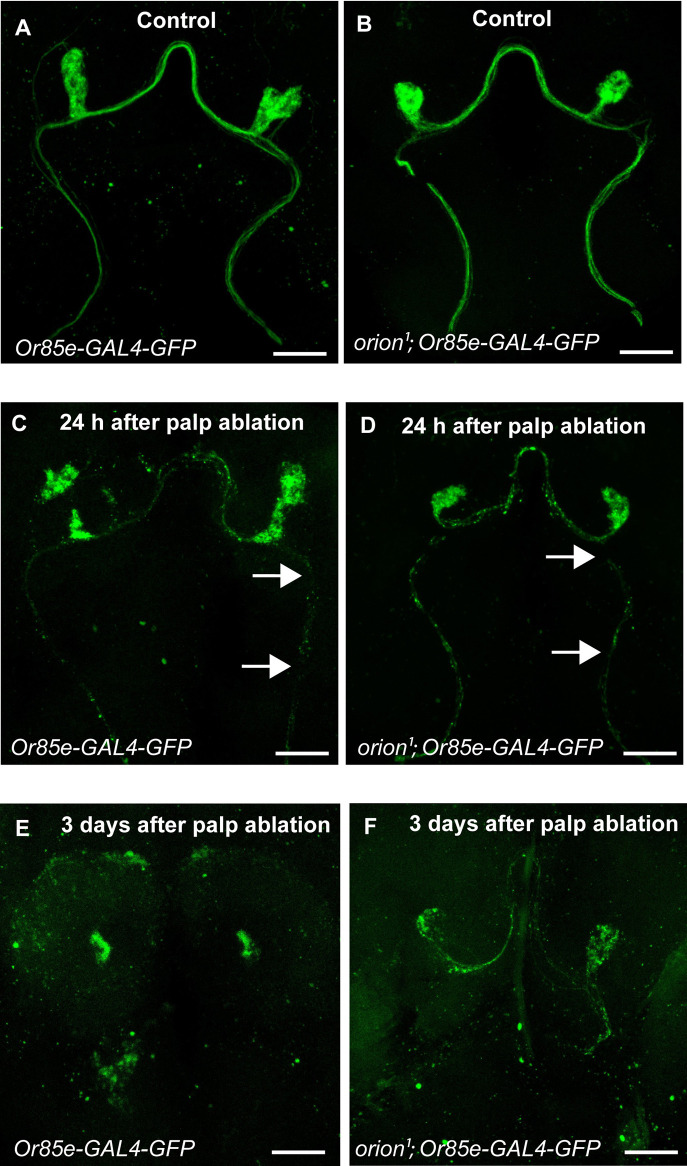
**Orion is not involved in the elimination of ORN debris by ensheathing glia after injury by palp ablation.** (A,B) Confocal *z*-stacks showing ORNs before injury (control) visualized by the expression of *Or85e-GAL4*-driven *UAS-mCD8-GFP* (green) in wild type (A) and *orion^1^* mutants (B). (C-F) Confocal *z*-stacks showing ORNs 24 h after palp ablation visualized by the expression of *Or85e-GAL4*-driven *UAS-mCD8-GFP* (green) in wild type (C) and *orion^1^* mutants (D) and 3 days after palp ablation in wild type (E) and *orion^1^* mutants (F). Note the axon discontinuities observed in both conditions in C and D, 24 h after palp ablation (arrows), and that only disseminated debris are observed in both conditions 3 days after palp ablation. Genotypes are listed in supplementary Materials and Methods, List of fly strains. *n*≥20 brains for each condition. Replicated twice. Scale bars: 30 µm.

After axotomy, the severed axon separated from its neuronal cell body degenerates by an evolutionarily conserved axon death signaling cascade. The resulting debris is cleared by surrounding glia, a process commonly known as Wallerian degeneration (Llobet Rosell and Neukomm, 2019). The surgical ablation of the antennae or maxillary palps severs ORN axons that project into the brain, triggering Wallerian degeneration (Doherty et al., 2009). Ensheathing glia infiltrate the injury site and engulf ORN axonal debris. In control animals, GFP^+^ axonal debris was cleared from the brain, whereas virtually all GFP^+^ degenerating ORN axons persisted in several mutants in which glial phagocytosis was blocked (Doherty et al., 2009; Musashe et al., 2016). Thus, this is a model of interest to test the involvement of Orion. We used flies expressing membrane-tethered GFP in a subset of maxillary palp ORNs (*Or85e-GAL4*). Our qualitative ORN structural analysis showed that in uninjured animals (control), ORN axons (labeled with mCD8-GFP) had a smooth morphology as they projected across the antennal lobe, and GFP intensity in glomeruli was very strong and presented membrane continuity in all the brains analyzed. These observations were similar in wild type and *orion^1^* (Fig. 7A,B). At 24 h after palp ablation, we observed discontinuity in axon fibers (dotted staining) outside of the glomeruli, reflecting a high level of Wallerian degeneration in both controls and *orion^1^* (Fig. 7C,D) and only traces of GFP^+^ axonal debris from glomeruli and fibers were present in wild type and *orion^1^* mutant brains 3 days after palp ablation (Fig. 7E,F). These results suggest that Orion is not required to eliminate ORN neurons by ensheathing glia after maxillary palp injury.

The wing serves as another well-established model to study the communication between severed axons and surrounding glia. The *Drosophila* marginal L1 wing vein allows for a graded level of axotomy, which results in two populations of axons: severed axons, cell bodies of which are distal to the injury site, and intact axons, cell bodies of which are proximal to the injury site (Neukomm et al., 2014). The axonal debris of severed mechanosensory and sensory neurons is cleared by wrapping glia (Neukomm et al., 2014). Sensory neuron MARCM clones in the wing expressing membrane-tethered GFP *(UAS-mCD8-GFP)* driven by *dpr1-GAL4* were subjected to axotomy, in wild-type and *orion^1^* heterozygous females as well as wild-type and *orion^1^* hemizygous males. Then, 7 days after axotomy (dpa), in the injured wing, the uninjured (control) axons, axonal debris traces and severed intact axons were scored. Age-matched uninjured wings served as controls.

**Fig. 8.**
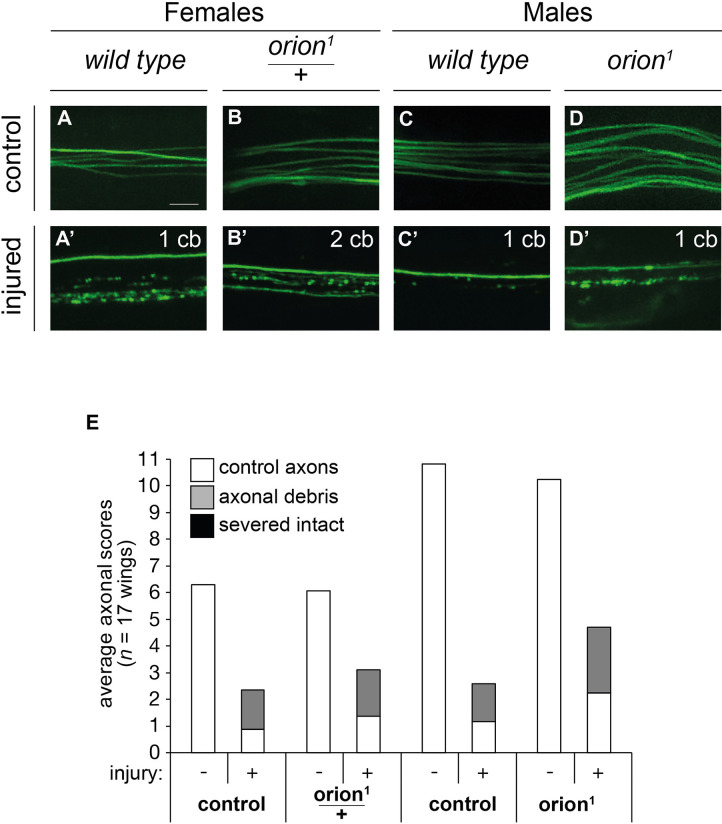
**Orion is dispensable for clearing axonal debris after axotomy in the wing.** (A-D′) Images are confocal *z*-stacks showing L1 vein axons labeled by *dpr1-GAL4*-driven *UAS-mCD8-GFP* (green). Representative pictures of control and injured (7 dpa) axons in wild type, heterozygous *orion^1^* and hemizygous *orion^1^* animals are shown. In the upper right corner of each image, the number of neuronal cell bodies (cb) indicates the number of uninjured, thus attached, neurons and therefore the expected intact axons in the field of view. (E) Quantification of average axonal scores of uninjured controls, debris and severed intact axons (white, gray and black, respectively). MARCM clones were generated in animals of the indicated genetic background. Genotypes are listed in supplementary Materials and Methods, List of fly strains. See Table S7 for raw data. Scale bar: 5 µm.

We found no extra severed intact axons at 7 dpa, suggesting that the execution of the axon death program is not affected by the *orion^1^* mutation (Fig. 8). We also found that the resulting axonal debris was cleared to a similar extent in all genotypes. This was in stark contrast to the *drpr*^Δ*5*^ mutation that impairs glial clearance and results in a complete penetrance of persistent axonal debris (Neukomm et al., 2014). It is worth mentioning that the MARCM clone numbers were higher in males than in females. However, clone numbers do not have any impact on the outcome of the injury assay. Together, these observations suggest that Orion is dispensable for the wrapping glia-mediated clearance of axonal debris in the L1 wing vein.

### Orion is dispensable for NMJ dismantling

In further analysis, we explored the effect of Orion on wrapping glia during NMJ developmental dismantling, which was previously described as a process involving retraction of wrapping glia preceding motor neuron retraction instead of degeneration (Boulanger et al., 2012). We investigated a potential role of motor neuron-secreted Orion that could affect NMJ dismantling by allowing wrapping glia dynamics. To do this, we studied muscle 4, at abdominal segment 3, NMJ development in larva, and at 6 h APF. Presynaptic motor neuron membranes were labeled with anti-HRP and postsynaptic muscle with anti-discs-large (DLG). Anti-HRP and anti-DLG staining of larval and pupal NMJs showed well-defined and organized synaptic boutons at L3 in controls and *orion^1^* mutants in 100% of the analyzed NMJ (Fig. S10A-F). By 6 h APF, the NMJs appeared to be completely disorganized in wild-type and *orion*-lacking animals (Fig. S10G-L), thus preventing the distinction of individual synaptic boutons. In addition, the postsynaptic components labeled with the anti-DLG antibody became fuzzy, and the DLG staining appeared to be completely fragmented and often absent in 90.3% of wild-type NMJ versus 89.2% of *orion^1^* NMJ. Thus, the similar NMJ morphology observed in controls and *orion^1^* mutants suggests no effect of Orion in NMJ-surrounding wrapping glia.

## DISCUSSION

### Role of Orion in engulfment and phagocytosis by astrocytes

Developmental remodeling of neural circuitry is a key strategy employed to prune redundant, inappropriate or interfering neurons to optimize connectivity (Wilton et al., 2019). Signals sent from dying neurons or neurites to be removed are received by appropriate glial cells. After receiving these signals, glia infiltrate degenerating sites, engulf, and clear neuronal debris through phagocytic mechanisms. We recently identified the chemokine-like Orion as a ligand, secreted by *Drosophila* MB γ neurons of the central brain, inducing astrocytic infiltration into the γ axon bundle (Boulanger et al., 2021). Thus, in *orion*-lacking flies, astrocytes are unable to infiltrate the γ axon bundle and, consequently, axon fragmentation-resulting debris is not eliminated during metamorphosis. Previous observations have shown a high elimination of neuronal processes and synaptic terminals along the VNC early in development by astrocytes (Tasdemir-Yilmaz and Freeman, 2014), which already infiltrate the neuropil before neuronal remodeling (Stork et al., 2014), differing from what occurs in MB bundles. This study shows that Orion has a crucial role in engulfment and phagocytosis of VNC neuronal debris. These data suggest that Orion not only orchestrates glia infiltration into the axonal bundle fated to degenerate but is also required to engulf remnant debris and for phagocytosis.

### Orion mediates the transformation of astrocytes into phagocytes in the VNC

Little is known about the pathways involved in glial activation. Astrocyte activation into phagocytes has been previously documented (Konishi et al., 2022; Lee and Chung, 2021; Morizawa et al., 2017). Glia activation is characterized by enlarged processes and abundant phagocytic vacuoles displaying phagolysosomal activity in the astrocyte cytoplasm. This activation depends on the expression of steroid hormone 20-hydroxyecdysone (ecdysone) receptor (EcR), which regulates the expression of *drpr*. Thus, loss of EcR signaling is sufficient to cell-autonomously suppress the transformation of astrocytes into phagocytes at pupariation (Tasdemir-Yilmaz and Freeman, 2014). Interestingly, we observed a similar phenotype of astrocyte vacuolated appearance and thick extensions in *orion*-lacking flies, suggesting that Orion mediates the overall transformation of astrocytes into phagocytes leading to engulfment and phagocytosis.

### Orion mediates the elimination of peptidergic neurons by cortex and ensheathing glia

MB γ neurons prune their medial and dorsal axon branches and dendrites at an early pupal stage, while their cell bodies remain. Later, at mid-pupal stages, they re-extend medial axon branches to establish adult-specific connectivity (Lee et al., 2000; Watts et al., 2004). In contrast, peptidergic vCrz^+^ and PDF-Tri neurons exhibit complete neurite degeneration, and their cell bodies undergo apoptotic death and are eventually eliminated. This elimination occurs at different developmental stages: the vCrz^+^ neuron apoptosis initiates in early pupae, whereas the PDF-Tri neuron apoptosis starts early after adult eclosion (Choi et al., 2006; Gatto and Broadie, 2011; Helfrich-Forster, 1997; Selcho et al., 2018). We show here that Orion is involved in eliminating both types of peptidergic neurons, extending the role of Orion to the elimination of apoptotic neurons. Interestingly, Orion is needed not only for the elimination of vCrz^+^ and PDF-Tri neurites, but also cell bodies. This suggests that Orion can also be presented to phagocytic cells by soma to be removed. Furthermore, as PDF-Tri neurons are eliminated in newly eclosed flies, our results formally extend the role of Orion to young adult stages.

Cortex glia appears to be essential for eliminating both types of neuronal cell bodies, as revealed by the high level of cortex glia engulfment around the vCrz^+^ and PDF-Tri cell bodies in wild types compared with *orion* mutants. Interestingly, we observed a high number of ensheathing glia extensions along the PDF-Tri neurites on both the MDBL and SEZ regions. The proximity of this type of glia to PDF-Tri axons in wild-type and *orion* mutant flies does not allow us to determine the engulfment degree of these axons. Nevertheless, as the lack of Orion induces a high amount of uncleared axonal PDF-Tri debris and based on our observations showing PDF-Tri debris surrounded by ensheathing glia in wild-type flies during PDF-Tri neuronal remodeling, we can anticipate that this type of glia is also a target of Orion. This idea is reinforced by the study of Vita et al. (Vita et al., 2021) suggesting that ensheathing glia drive the clearance of PDF-Tri neurons. Together, these data provide evidence that Orion is able to signal via astrocyte, cortex and ensheathing glia, depending on the neuronal remodeling paradigm.

### *orion* is dispensable in two adult axonal injury paradigms

Recent studies have shown that *orion* is required for the elimination of larval dendritic arborization (da) dendrites by phagocytic epidermal cells after laser ablation at larval stages (Ji et al., 2023). To extend the role of *orion* to axonal injury paradigms, we explored two distinct antennal and wing axotomy models in *Drosophila*. Orion is not involved in the clearance of the ORN axonal debris, as a similar level of axon debris clearance is observed in controls and *orion^1^* mutants after ORN axotomy. In the *Drosophila* L1 wing vein, wrapping glia eliminate the debris from injured axons. After axotomy, Orion is not involved in the communication between severed axons and wrapping glia, suggesting that *orion* is not required for proper glial clearance after axotomy in adult flies, which contrasts with the dendrite injury model in larvae. Concerning the lack of *orion* effect in NMJ dismantling, it could be explained by the fact that this is a retraction process not implying phagocytosis. Thus, *orion* plays a crucial role during development, but not in adult flies (see Table 1).

**Table 1.**
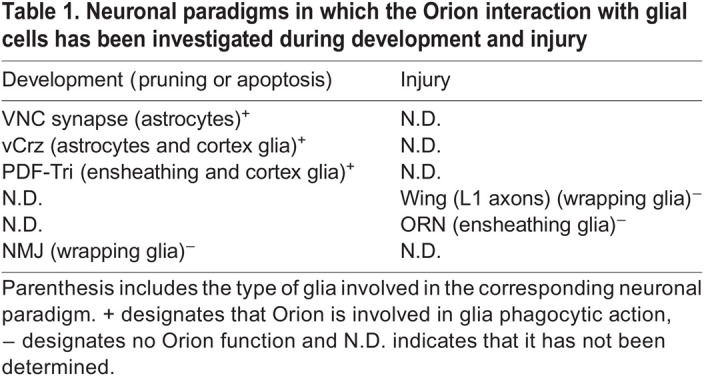
Neuronal paradigms in which the Orion interaction with glial cells has been investigated during development and injury

### Orion expression and sources

*orion* expression in MB is necessary and sufficient for γ neuron remodeling (Boulanger et al., 2021). In contrast, *orion* expression is not required in da neurons for their remodeling (Ji et al., 2023). Moreover, expression of *orion* in the fat body is sufficient to rescue da remodeling phenotypes, suggesting, in this case, sources of Orion external from the neurons to be remodeled. Similarly, here, our data led us to hypothesize that secretion of Orion from sources other than vCrz and PDF-Tri neurons is sufficient to rescue vCrz and PDF-Tri neuron remodeling phenotypes seen in an *orion* mutant (*elav-GAL4; UAS-orion*). The specific *orion* expression in both types of remodeling neurons, which does not rescue *orion* mutant phenotypes (*Crz-* and *PDF-GAL4; UAS-orion*), and the specific *UAS-orion-RNAi* expression, which does not block their pruning, are in accordance with this hypothesis.

Orion is required in neurons other than Crz for vCrz neuronal remodeling (*elav-GAL4; UAS-orion-RNAi*). This implies that other neuron-secreted Orion targets vCrz neurons to be remodeled. For the PDF-Tri neuronal remodeling, we could not conclude about the source of Orion for technical reasons (leakiness of the *UAS-orion-RNAi* at adult stage).

One can note that, even if neuronal sources of Orion are required for vCrz neuronal remodeling, it does not preclude that other sources of Orion are not also required. Multiple sources of Orion could also be the case for the PDF-Tri neuronal remodeling. In accordance with this hypothesis, it is reported that a strong expression of Orion was observed during development in many tissues, such as fat body, epidermal cells, trachea, hemocytes and glia (Ji et al., 2023). Therefore, MBs could be a particular case in which Orion expression is required in the neurons to be remodeled. This could be due to the requirement of glia infiltration into the MB bundles, an initial step of pruning not required for remodeling of individual neurons. Consequently, this suggests that Orion has two functions: one specific for glia infiltration in MB bundles and one general for phagocytosis of neuronal debris.

## MATERIALS AND METHODS

### *Drosophila* stocks

All crosses were performed using standard culture medium at 25°C. Except where otherwise stated, alleles have been previously described (http://flystocks.bio.indiana.edu). *orion* mutants (*orion^1^* and *orion^ΔC^* were produced in a previous study from our laboratory (Boulanger et al., 2021). *UAS-orion-RNAi* comes from Vienna *Drosophila* Resource Center (stock 30843); *Crz-GAL4* and *UAS-Casor* were provided by Jae H. Park (University of Tennessee, TN, USA), *alrm-GAL4* and *alrm-GAL4 UAS-GFP repoflp^6^; FRT2A, Tub-GAL80* were from Marc Freeman (Vollum Institute, Oregon Health & Science University, OR, USA), *UAS-Rab7-GFP* was provided to us from Chun Han (Weill Institute, Cornell University, NY, USA) and comes from Hugo Bellen (Baylor College of Medicine, University of Houston, TX, USA). We used ten *GAL4* lines: *201Y-GAL4* expressed in γ MB neurons, *PDF-GAL4* expressed in all PDF neurons, *Crz-GAL4* expressed in central and ventral Crz neurons, *Or85e-GAL4* expressed in ORNs, the pan-neuronal drivers *elav-GAL4*, *dpr1-GAL4* (Nakamura et al., 2002), expressed in the wing nerve of wing vein L1, *NP2222-GAL4* expressed essentially in cortex glia, *MZ0709-GAL4* expressed in ensheathing glia, *alrm-GAL4* expressed exclusively in glial astrocytes (Doherty et al., 2009; Stork et al., 2014) and the pan-glial driver *repo-GAL4* expressed in all glia (Sepp et al., 2000).

### Adult brain dissection, immunostaining and astrocyte clone visualization

Adult fly heads and thoraxes were fixed for 1 h in 3.7% formaldehyde in phosphate-buffered saline (PBS) and brains were dissected in PBS. For larval and pupal brains, brains were first dissected in PBS and then fixed for 15 min in 3.7% formaldehyde in PBS. They were then treated for immunostaining as previously described (Boulanger et al., 2021). Antibodies, obtained from the Developmental Studies Hybridoma Bank, were used at the following dilutions: mouse monoclonal anti-Fas2 (1D4) 1:10; mouse monoclonal anti-bruchpilot (nc82) 1:25; mouse monoclonal anti-PDF (PDF C7) 1:50; mouse monoclonal anti-discs large (4F3) 1:100. Anti-Crz rabbit antibody (from Jan Veenstra, University of Bordeaux, France) was used at 1:2000. Goat secondary antibodies conjugated to Cy3 and Cy5 against mouse or rabbit IgG (Jackson ImmunoResearch, 115-166-006, 711-165-152, 711-175-152), Alexa488 and Alexa647 (Invitrogen, A11029, A21236) were used at 1:300 for detection, Cy3-conjugated goat anti-HRP was used at 1:100 (Jackson ImmunoResearch, lot: 85411). DAPI (Sigma-Aldrich) was used at 1:1000 from a 10 mg/ml stock solution. To visualize astrocyte clones in the VNC, first instar larvae were heat-shocked at 37°C for 1 h. Adult brains were fixed for 15 min in 3.7% formaldehyde in PBS before dissection and GFP visualization. For lysotracker staining, VNC were dissected in 1× PBS without fixation and incubated in a lysotracker solution (Lysotracher Red DND-99, Invitrogen, 1:5000) for 15 min, then washed and fixed as previously described.

### Microscopy and image processing

Quantitation of intensity was performed using ImageJ software. Images were acquired at room temperature using a Zeiss LSM 780 and a Leica SP8 laser scanning confocal microscope (MRI Platform, Institute of Human Genetics, Montpellier, France) equipped with a ×40 PLAN apochromatic 1.3 oil-immersion differential interference contrast objective lens. The immersion oil used was Immersol 518F. The acquisition software used was Zen 2011 (black edition). Contrast and relative intensities of the green (GFP), of the red (Cy3) and of the blue (Cy5 and Alexa 647) channels were processed using ImageJ and Fiji software. Settings were optimized for detection without saturating the signal. For each set of figures, settings were constant.

### Quantitation of immunolabeling

For nc82 signal quantitation, we used the ImageJ software and we performed ten measurements for each picture, five in the thoracic region (neuromeres t1-t3) and five in the abdominal region (neuromeres a1-a8), (intensity 1, intensity 2, etc.) of the VNC neuropil and the same number of measurements in the background (VNC cortex region) using confocal single slices. The mean of these background measurements is called mean background. We then subtracted intensities of mean background from each intensity value (intensity 1 minus mean background, etc.) to obtain normalized intensity values. Finally, we compared normalized intensity values between two genetic conditions. To quantify phagocytic vesicles, we manually counted the total number of vesicles observed in a VNC randomly chosen confocal 2D plan. We considered a vesicle as a clearly distinguishable GFP^+^ ring. To determine vCrz^+^ and PDF-Tri cell body engulfment degree, we established two categories of phenotypes: ‘engulfed’, when vCrz^+^ cell bodies were surrounded or contacted by expanded glial membranes strongly labeled with GFP forming phagocytic cups, and ‘unengulfed’, when the GFP^+^ glial membrane surrounding the cell body was either thin or not apparent at the set laser gain (which does not imply its absence). We used the Imaris (Bitplane) software to generate 3D structures of glia-surrounded vCrz^+^ or PDF-Tri neuron cell bodies from regular confocal images to determine their engulfment degree (engulfed, unengulfed). The images were taken using the same confocal settings within the same set of experiments, and the data were processed in parallel. The experiments were repeated at least twice. To quantify vCrz^+^ neurites we analyzed three types of GFP^+^ neurites: horizontal axons (eight tracks), medial and lateral axons (two tracks). We considered an axon present when the GFP staining was not disrupted along the whole track. Only horizontal axons were quantified with the anti-Crz antibody, because they are shorter and easier to follow all along the process. To quantify vCrz^+^ cell bodies (16 cell bodies), we considered only dots bigger than surrounding debris and located at the right emplacement on the VNC. To quantify the *Rab7-GFP* vesicles, for each phenotype the total number of *Rab7-GFP* donuts comprising vCrz-soma-derived debris was manually quantified in serial *z*-sections including all of the vCrz^+^ cell bodies. To ensure that the quantified vesicles contained soma-derived debris included in cortex-glia and not astrocytes, we only quantified vesicles detaching from the vCrz soma. To quantify the Casor probe GFP localization, we considered nuclei (when the fluorescence was only nuclear), cytoplasm (when only cytoplasmic GFP expression was observed) and nuclei plus cytoplasm (when GFP expression was homogeneously distributed between nuclei and cytoplasm). For PDF-Tri neuron surface, measures were performed using ImageJ software. The outline of the PDF-Tri staining was drawn using the freehand selection tool on a *z*-stack of all the sections comprising PDF-Tri neurons in which we merged GFP and anti-PDF staining. The number of somas at each timepoint and in each condition was calculated based on confocal images. Quantifications were performed unaware of genotype.

### Injury (axotomy) assay in the wing

Animals were kept at 20°C for 5-7 days before axotomy, unless stated otherwise. Axotomy was performed as previously described (Llobet Rosell et al., 2022; Paglione et al., 2020). One wing per anesthetized fly was cut approximately in the middle. Flies were returned to individual vials. Wings were mounted onto a slide at 7 dpa and imaged using a spinning disk microscope to assess and quantify for intact or degenerated axons, as well as the remaining uninjured control axons.

### Statistics

Comparison between two groups expressing a qualitative variable was analyzed for statistical significance using the Fisher's and Chi-squared exact tests. Comparison of two groups expressing a quantitative variable was analyzed using the two-sided nonparametric Mann–Whitney *U* test (BiostaTGV: http://biostatgv.sentiweb.fr/?module=tests). Values of *P*<0.05 were considered to be significant. Graphs were created using the GraphPad Prism software (version 8.1.1). Statistical significance was defined as: ****P<0.0001; ***P<0.001; **P<0.01; *P<0.05; ns, not significant. The sample size of each group (*n*) is included in a parenthesis in figures.

## Supplementary Material

10.1242/develop.201633_sup1 Supplementary information
