# Supplementary material for: Chemokine-like Orion is involved in the transformation of glial cells into phagocytes in different developmental neuronal remodeling paradigms
Source: Development. 2023 Oct 2;150(19):dev201633. doi: 10.1242/dev.201633 (PMC10565233; doi:10.1242/dev.201633)
Supplement: Supplementary information [file develop-150-201633-s1.pdf]

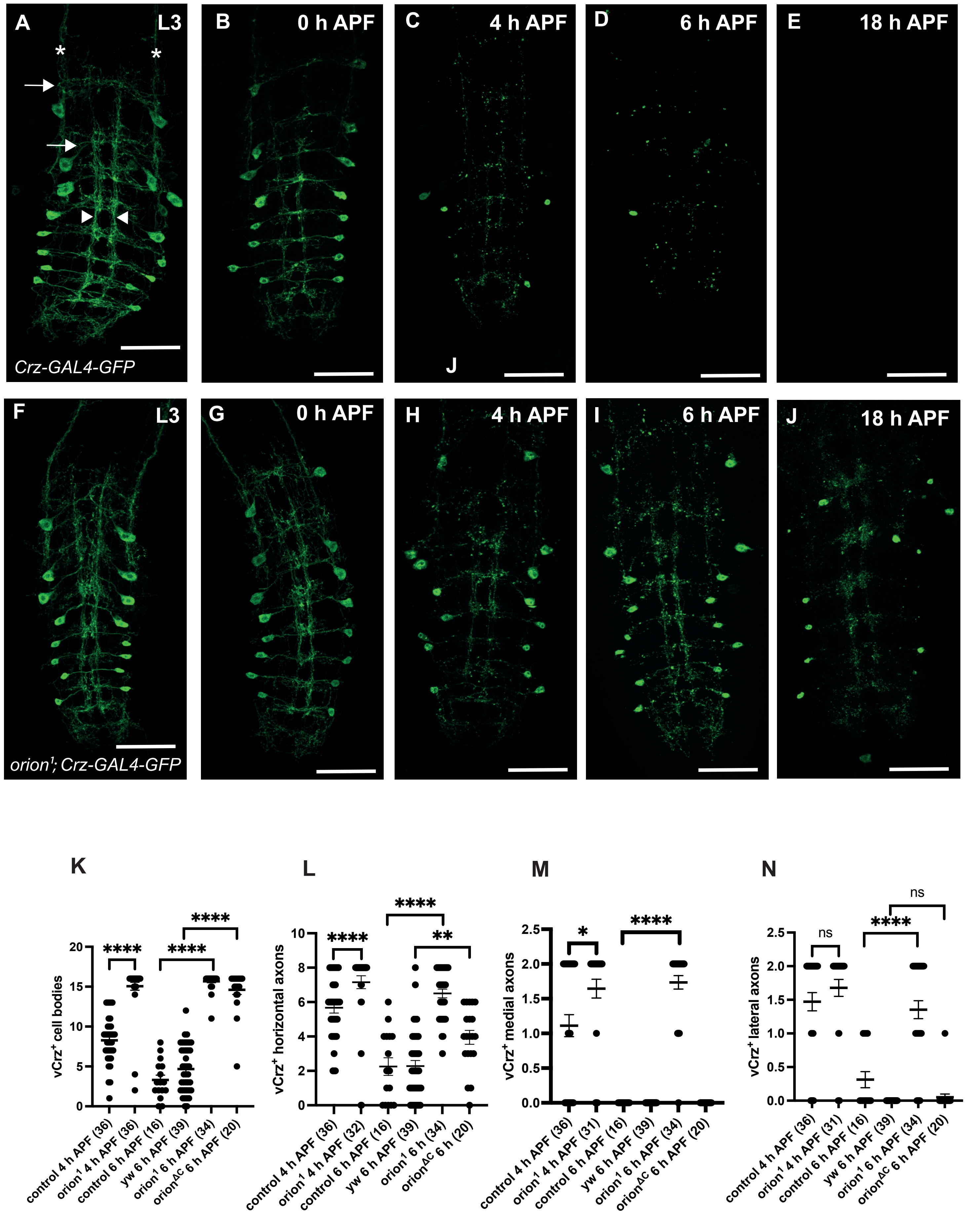

Development • Supplementary information

Fig S1. Orion is required for the elimination of vCrz$^+$ cell bodies and axons. (A-J) Confocal z-stacks showing vCrz$^+$ neurons visualized by the expression of *Crz-GAL4-* driven *UAS-mCD8-GFP* (green) at the indicated developmental stages in control **(A-E)** and *orion^1* mutants **(F-J)**. Horizontal axons were labeled by arrows, medial and lateral axons were labeled by arrowheads and an asterisk respectively in A. **(K)** Quantification of the number of vCrz$^+$ cell bodies, **(L)** vCrz$^+$ horizontal axons, **(M)** vCrz$^+$ medial axons and **(N)** vCrz$^+$ lateral axons in controls and *orion* mutants at 4 h and 6 h APF. Genotypes are listed in Supplementary list of fly strains. n values are indicated in a parenthesis for each condition, replicated twice Error bars represent mean ± SEM (Mann-Whitney *U* test). Scale bars is 70 µm.

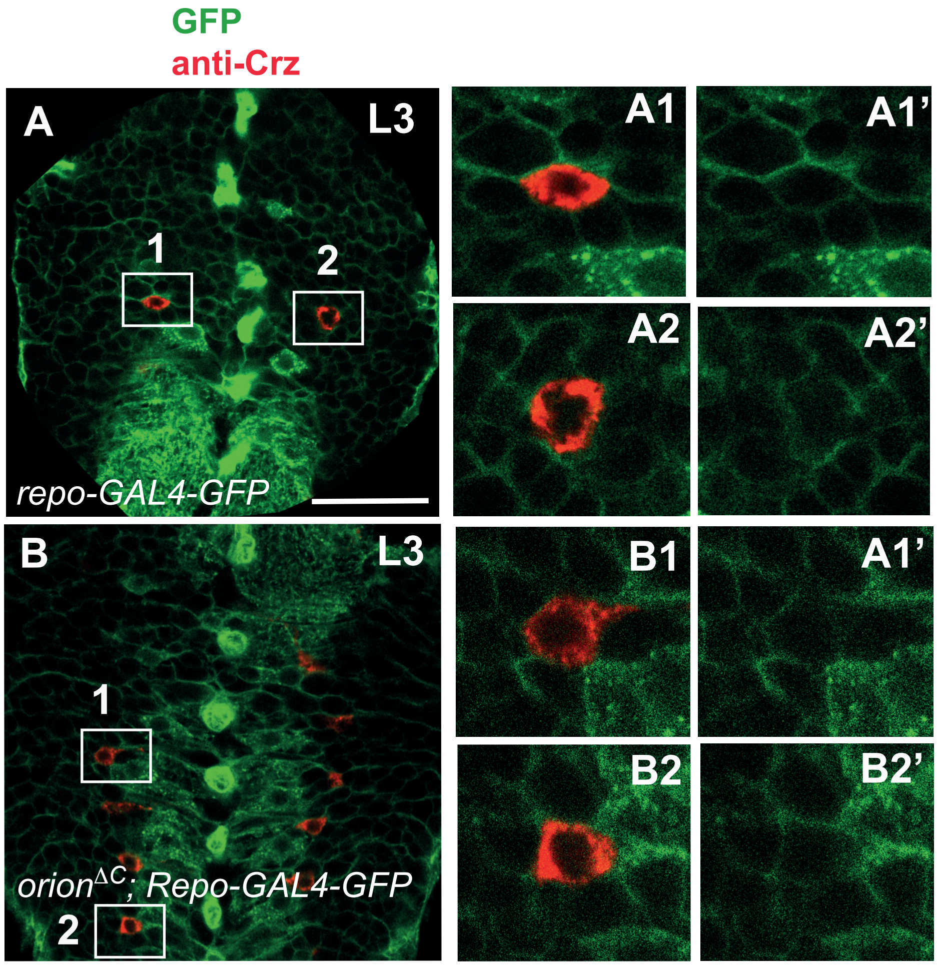

Development • Supplementary information

Fig S2. The morphology of cortex glia surrounding vCrz$^+$ cell bodies is not affected by the *orion* mutation early in development. (A, B) Confocal plans showing *UAS- GFP* expression driven by *repo-GAL4* (green) and anti-Crz staining (red) in controls **(A)** and *orion* mutants **(B)** VNCs at larval stage (L3). Insets A1, A2, B1, B2 are high magnification of boxes in A and B showing vCrz$^+$ cell bodies surrounded by cortex glia. Note the similar morphology of the cortex glia in controls A1, A2 and mutants B1, B2. A1', A2', B1' and B2' are single channels of A1, A2, B1, B2 respectively. Genotypes are listed in Supplementary list of fly strains, n = 17 somas for wild type and 39 for *orion* mutants. Scale bars are 30 µm.

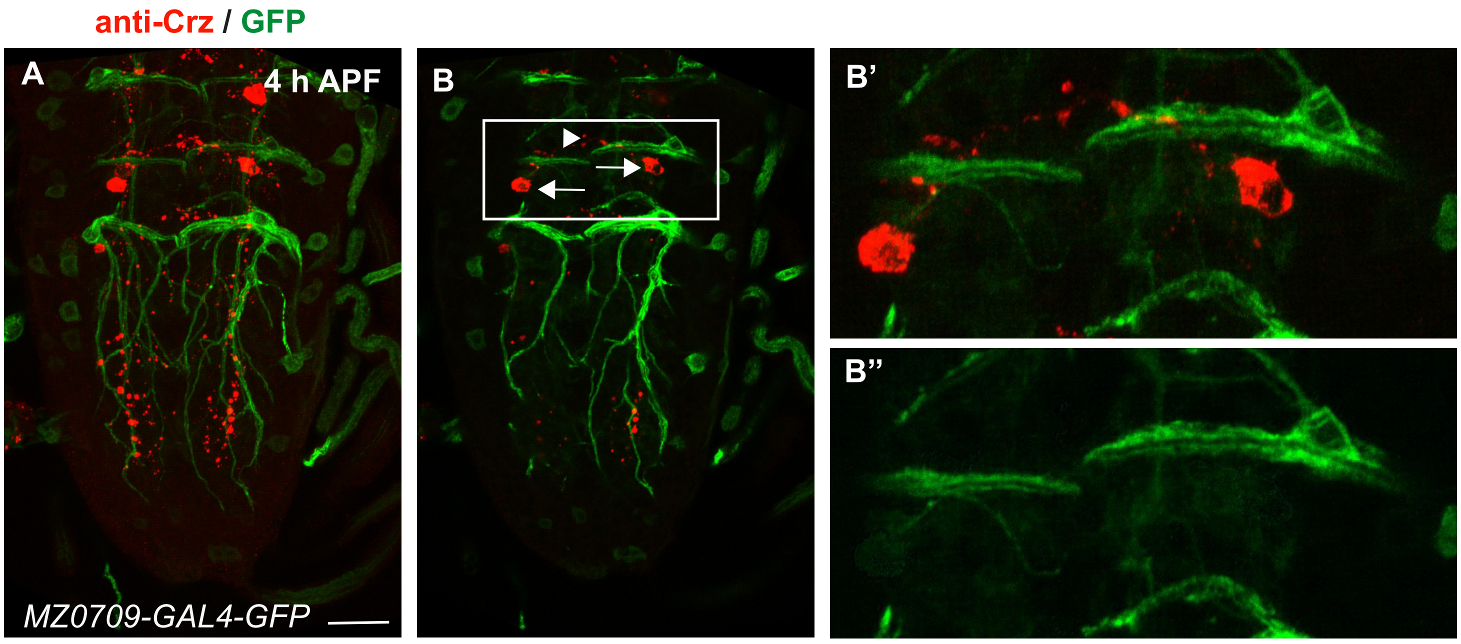

Fig S3. The elimination of vCrz[+] neurites and cell bodies does not seem mediated by ensheathing glia. (A, B) Ensheathing glia visualized by the expression of *MZ0709-GAL4*-driven *UAS-mCD8-GFP* (green) and vCrz[+] neurons by an anti-Crz antibody (red) at 4 h APF. **(B)** Arrows point to vCrz[+] cell bodies, which are not reached by ensheathing glia extensions and arrowhead to unengulfed neurites. See inset for higher magnifications of B in **B'** and **B''**. **A** is a z-projection confocal image, **B** is a single confocal plan. Genotypes are listed in Supplementary list of fly strains, n = 8, replicated twice. Scale bar is 30 µm.

Development • Supplementary information

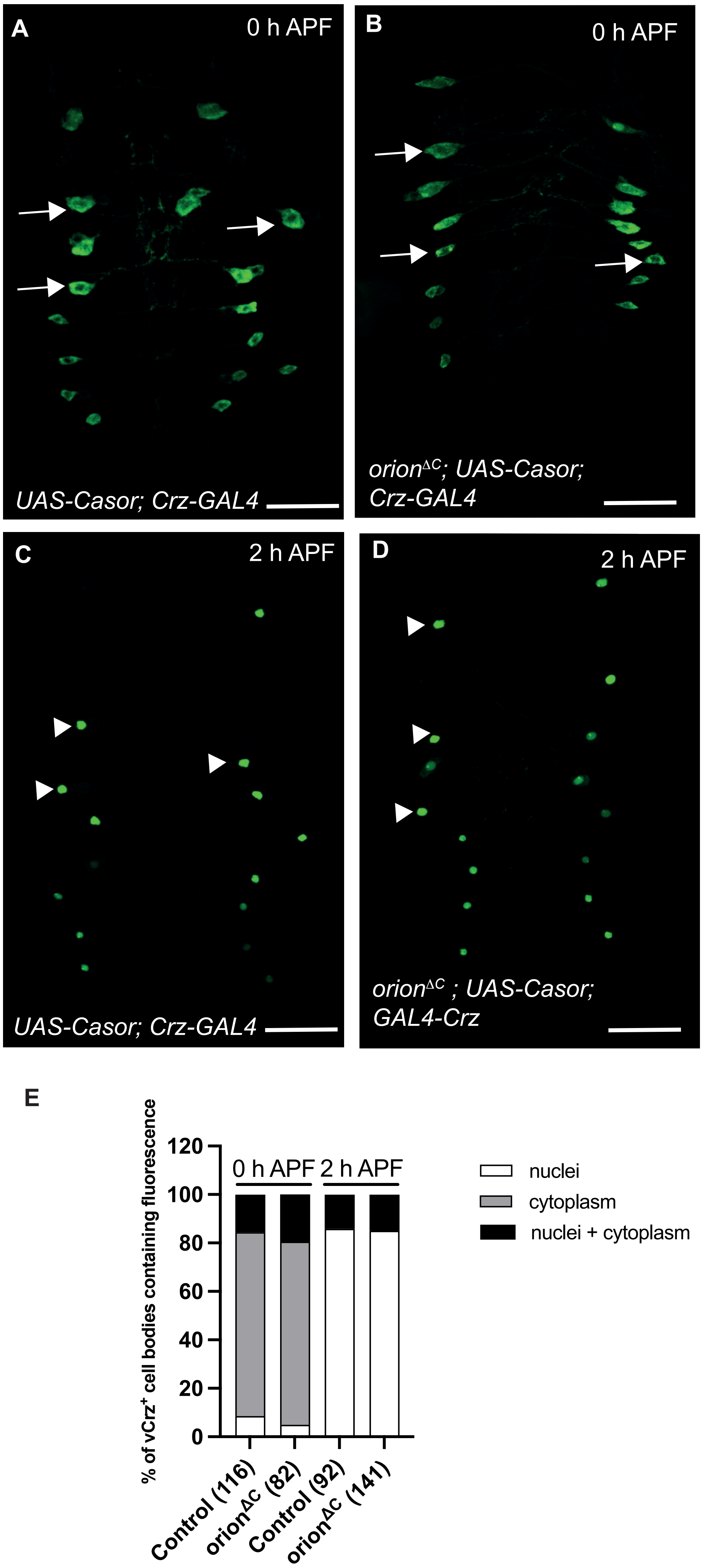

Development • Supplementary information

Fig S4. Nuclear targeting of Casor probe in the vCrz neurons. Confocal z- stacks showing vCrz cell bodies visualized by the expression of *Crz-GAL4*-driven *UAS-Casor* probe (green) **(A, B)** Intact casor probe monitored by intrinsic GFP was detected in most of the soma of controls **(A)** and *orion*$^{\Delta C}$ **(B)** at 0 h APF (arrows). **(C, D)** Cleaved Casor probe was detected mainly in the nuclei in controls **(C)** and *orion*$^{\Delta C}$ **(D)** (arrowheads) at 2 h APF. Scale bar is 40 µm **(E)** Percentage of vCrz^{+} cell bodies containing Casor-GFP fluorescence in the nuclei, cytoplasm or in both nuclei and cytoplasm (nuclei + cytoplasm) at 0 and 2 h APF. The number of cell bodies is included in a parenthesis for each condition (see raw data for number of animals). Replicated twice. Genotypes are listed in Supplementary list of fly strains.

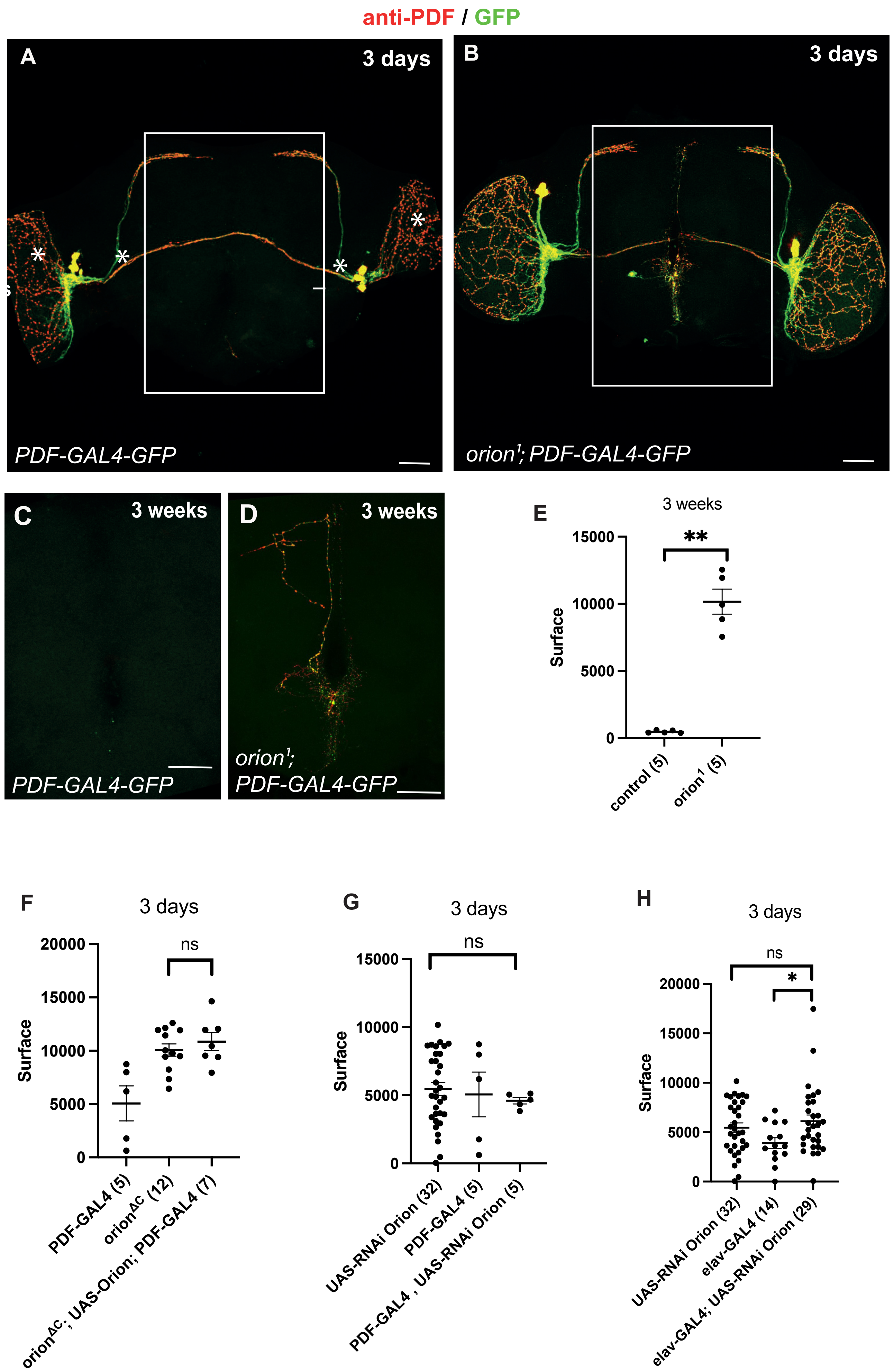
anti-PDF / GFP
A
3 days
PDF-GAL4-GFP
B
3 days
orion1; PDF-GAL4-GFP
C
3 weeks
PDF-GAL4-GFP
D
3 weeks
orion1;
PDF-GAL4-GFP
E
3 weeks
Surface
**
control (5)
orion1 (5)
F
3 days
ns
PDF-GAL4 (5)
orionΔC (12)
orionΔC; UAS-Orion; PDF-GAL4 (7)
G
3 days
ns
UAS-RNAi Orion (32)
PDF-GAL4 (5)
PDF-GAL4 , UAS-RNAi Orion (5)
H
3 days
ns
*
UAS-RNAi Orion (32)
elav-GAL4 (14)
elav-GAL4; UAS-RNAi Orion (29)

Fig S5. Orion mutant retains developmentally transient PDF-Tri neurons. **(A-D)** Confocal z-stacks showing PDF **(A, B)** and PDF-Tri **(C, D)** neurons visualized by the expression of *PDF-GAL4*-driven *UAS-mCD8-GFP* (green) and labeled with anti-PDF antibody (red) at the indicated time points in wild type **(A, C)** and *orion*[1] mutants **(B, D).** Note that the whole PDF neuron network is seen in A and B (asterisk in A) and that no differences are observed between controls and mutants in PDF regions other than the PDF-Tri one. Genotypes are listed in Supplementary list of fly strains. Scale bar is 50 µm. **(E-H)** Surfaces (in μm^2) occupied by the PDF-Tri arborization at 3 weeks in controls and *orion* mutants (E), as well as at three days (F-H) in condition of *orion* forced-expression driven by *PDF-GAL4* **(F)** and expression of *UAS-Orion-RNAi* driven by *PDF-GAL4* **(G)** and *elav-GAL4* **(H).** Error bars represent mean ± SEM (Mann-Whitney *U* test). The number of brains analyzed is included in a parenthesis for each condition. Replicated at least twice except for C-E which was performed only once as a verification of data from Figure 5 I-L

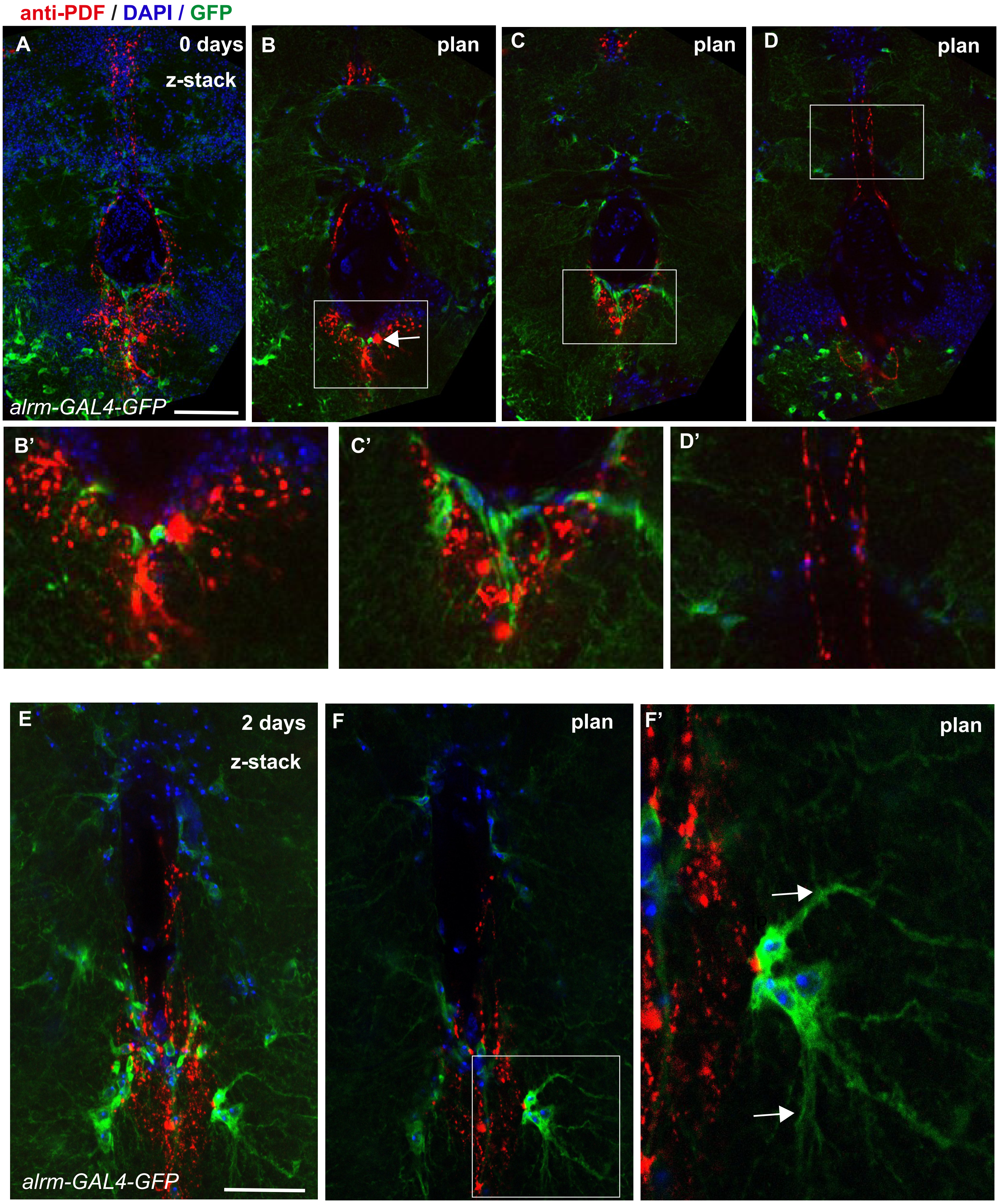

Development • Supplementary information

Fig. S6. Astrocytes surrounding PDF-Tri neurons do not seem to eliminate PDF-Tri neurites. (A, E) Confocal z-stacks showing astrocytic glia, PDF-Tri neurons and nuclei respectively visualized by the expression of *alrm-GAL4*-driven *UAS-mCD8-GFP* (green), an anti-PDF antibody (red) and DAPI (blue) at 0 **(A)** and two **(E)** days. Different regions of the brain contained in these z-stacks are shown as single confocal plans (**B, C, F)** (SEZ region), (**D)** (MDBL region). Rectangles border regions showing PDF-Tri dendrites close to astrocytic glia, but engulfment is not observed. These regions are shown at higher magnification in **B'**, **C'**, **D'** and **F'**. Note the long and thin structure of the filamentous processes of the astrocytes in 2 days flies (arrows) that does not engulf neurites. A-D four independent experiments, E-F, two independent experiments. Scale bars are 50 µm (A-D) and 30 µm (E-F).

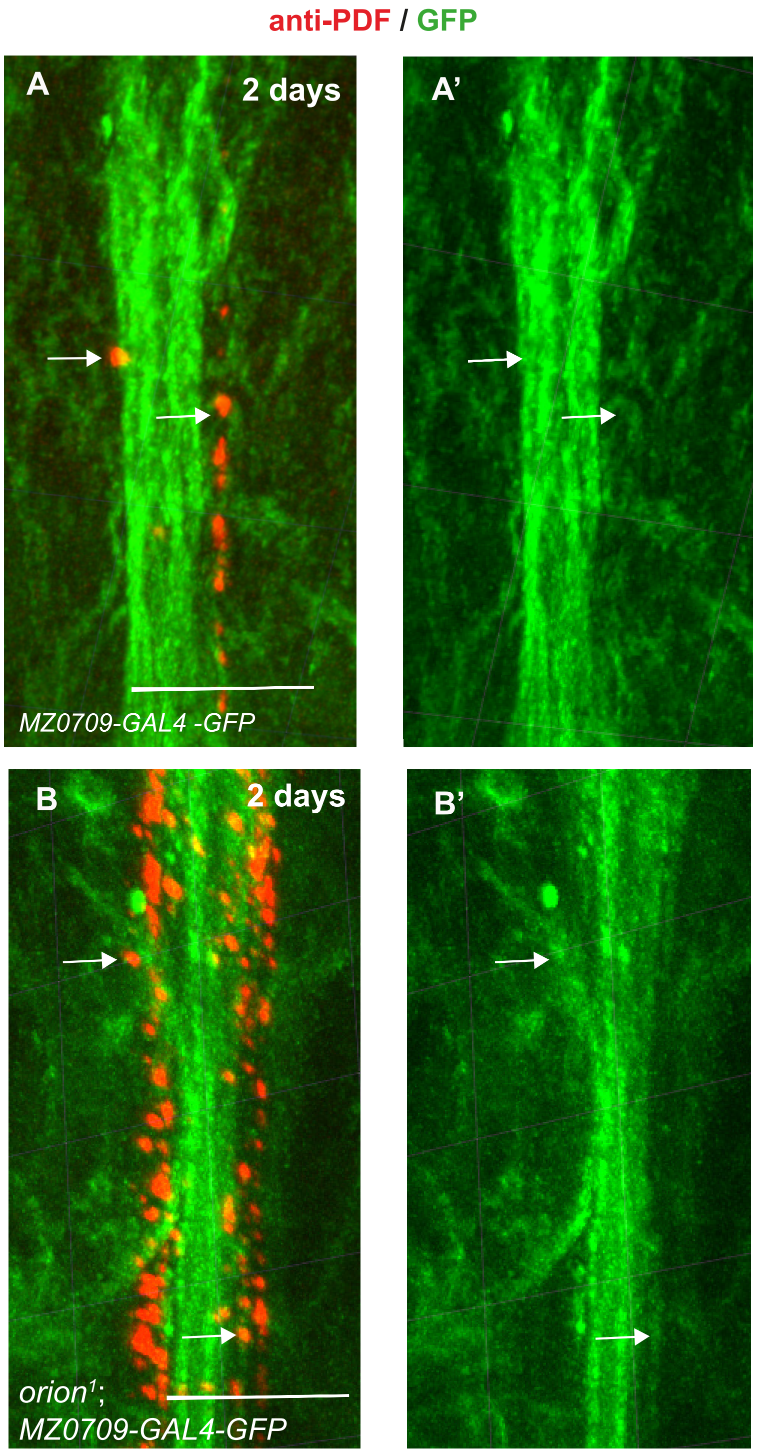

Development • Supplementary information

Fig S7. Ensheathing glia enwrap PDF-Tri labeled axons in a similar way in control and *orion* mutants. (A- B) Confocal 3D images showing ensheathing glia visualized by the expression of *MZ0709-GAL4*-driven *UAS-mCD8-GFP* (green) and PDF-Tri neurons labeled with anti-PDF antibody (red) at two days in control **(A)** and mutant **(B)** flies. **A'** and **B'** are single channel of **A** and **B** respectively. Glial cells surrounding a PDF-Tri labelled axons are pointed by arrows. Note that in both cases PDF-Tri-labelled axons are wrapped in ensheathing glia. In control flies, PDF-labeled axonal debris disappear surrounded by ensheathing glia (see the smaller amount of PDF red staining in controls **(A)**, compared to *orion* mutants **(B)**), while in *orion* mutants the PDF-labeled axon track is retained. Genotypes are listed in Supplementary list of fly strains, n = 9 VNCs for wild type and 9 for *orion* mutants. Scale bars are 20 µm.

Development • Supplementary information

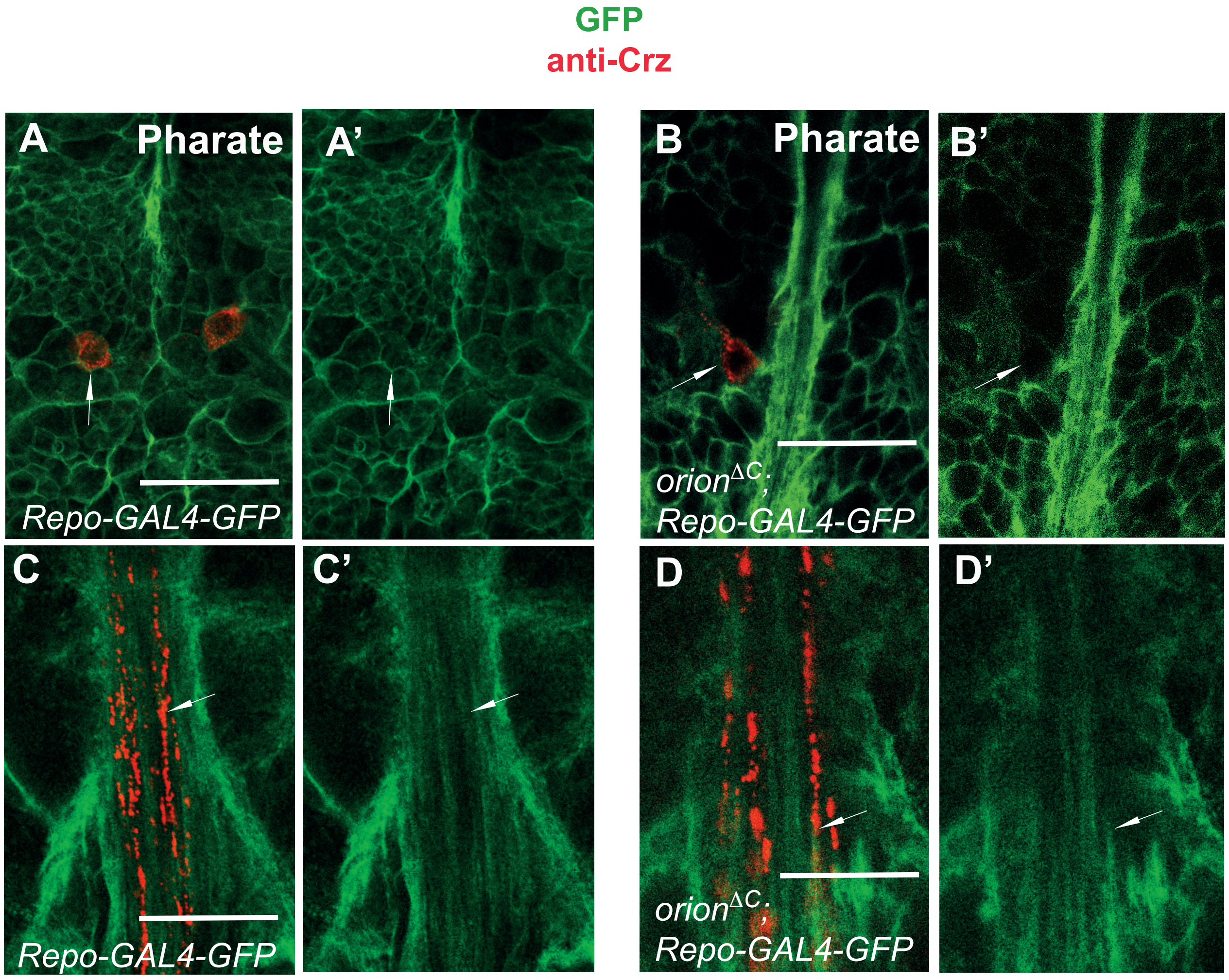

Development • Supplementary information

Fig S8. The morphology of cortex and ensheathing glia surrounding PDF- Tri cell bodies and axons respectively is not affected by the *orion* mutation early in development. (A-D) Confocal plans showing *UAS-GFP* expression driven by *repo-GAL4* (green) and anti-PDF staining (red) in controls **(A, C)** and *orion* mutants **(B, D)** at pharate stage. Arrows point to cortex glia in **A'** and **B'** and ensheathing glia in **C'** and **D'**. Note the similar morphology of the cortex and ensheathing glia in control and *orion* mutants in regions close to cell bodies and axons. A', B', C' and D' are single channels of A, B, C and D respectively. Genotypes are listed in Supplementary list of fly strains, n = 10 for wild type and 9 for *orion* mutants. Scale bars are 30 µm.

Development • Supplementary information

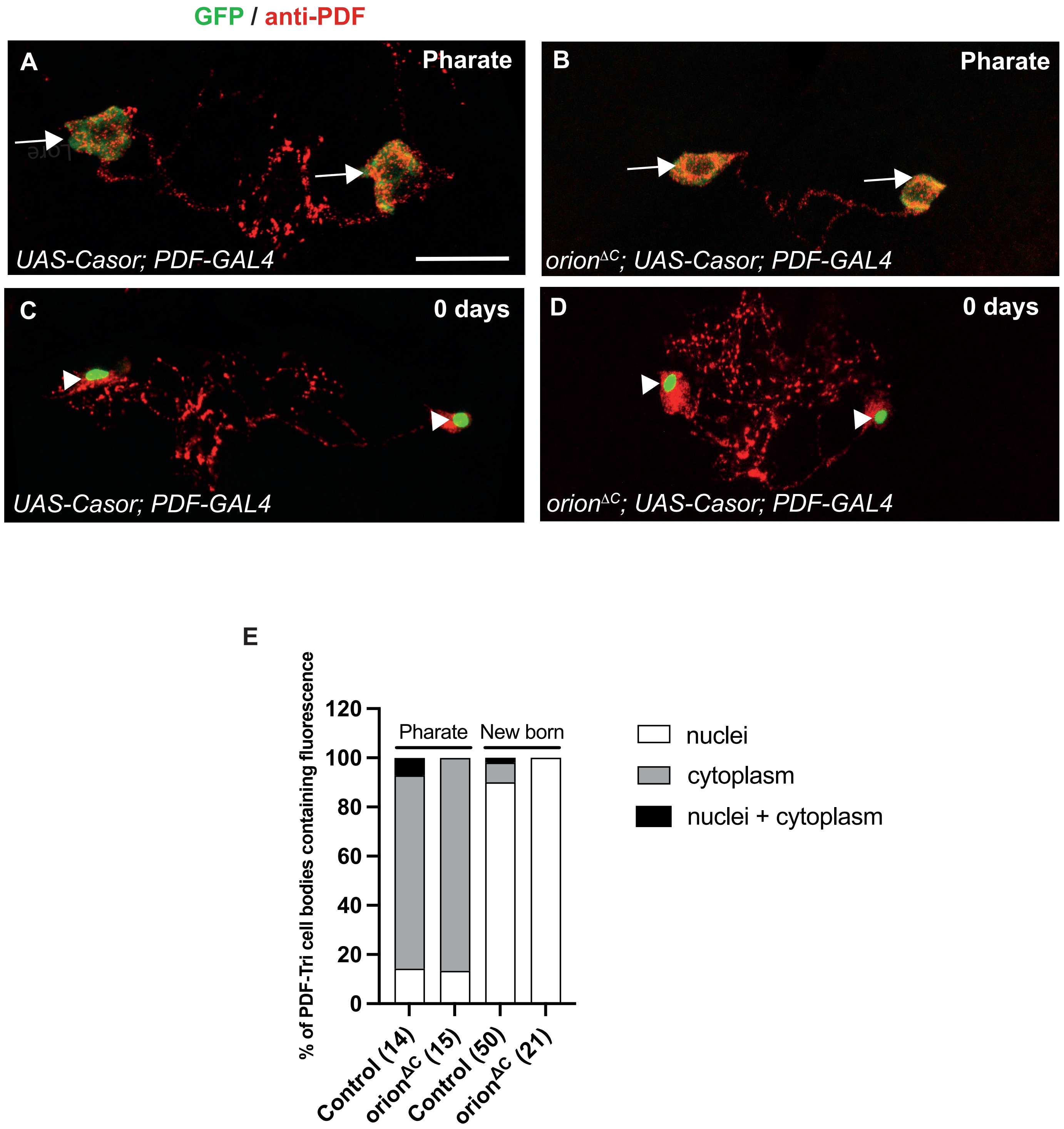

Development • Supplementary information

Fig. S9. Nuclear targeting of Casor probe in PDF-Tri neurons. Confocal z- stacks showing PDF-Tri cell bodies visualized by the expression of *PDF-Tri-GAL4*-driven *UAS-Casor* probe (green) and anti-PDF antibody co-staining (red) **(A, B)** Intact Casor probe monitored by intrinsic GFP was detected in most of the soma of controls **(A)** and *orion*$^{\Delta C}$ **(B),** at late pupal stage **(C, D)** Cleaved Casor probe was detected in most of the nuclei of controls **(C)** and *orion*$^{\Delta C}$ **(D)** new born (0 days) flies (arrowheads). Genotypes are listed in Supplementary list of fly strains. Scale bar is 30 µm **(E)** Representation of the percentage of PDF-Tri cell bodies containing Casor-GFP fluorescence in the nuclei, cytoplasm or both nuclei and cytoplasm (nuclei + cytoplasm) in pharates and new born flies. The number of cell bodies is included in a parenthesis for each condition (see raw data for number of animals).

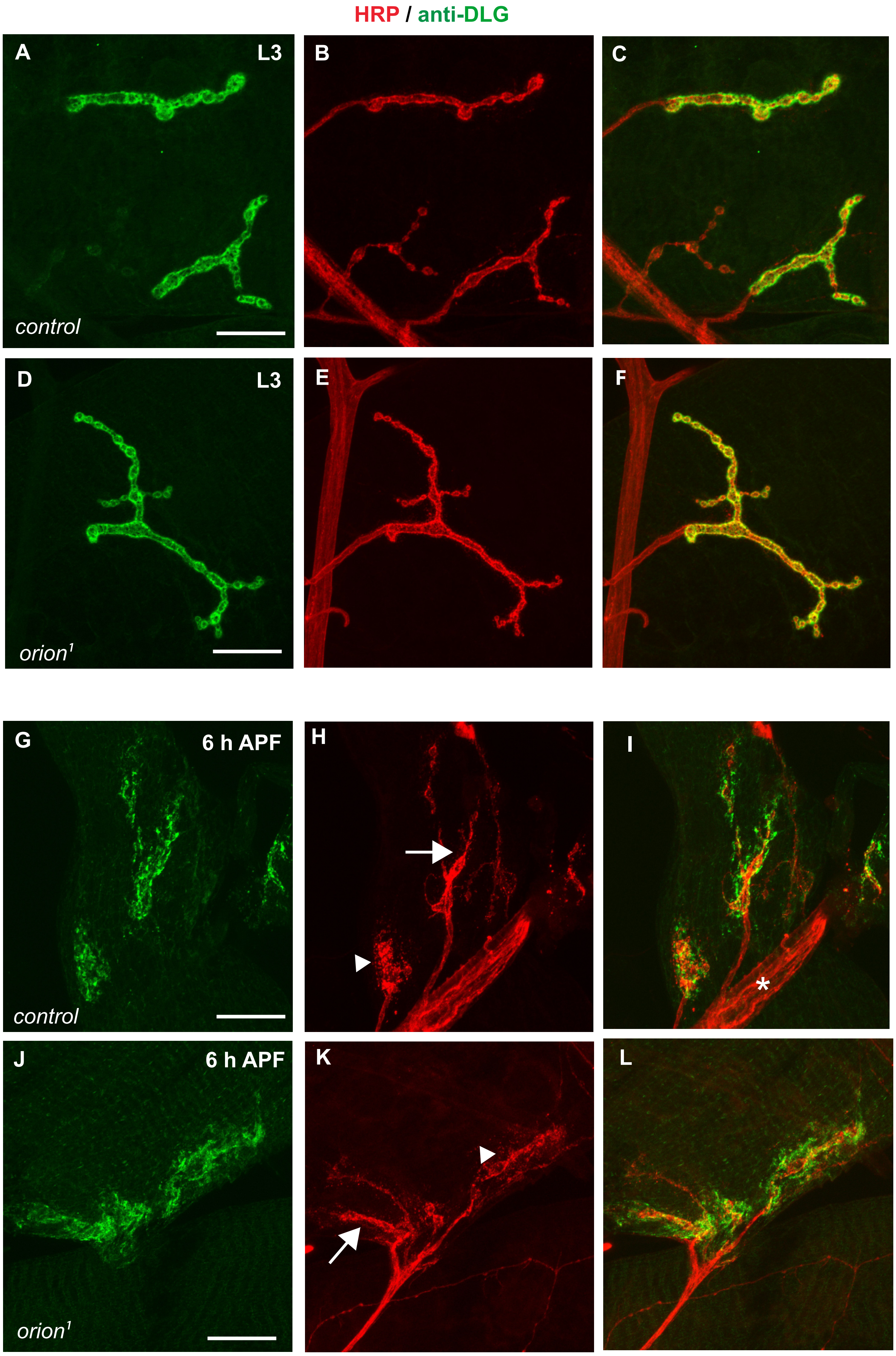

Development • Supplementary information

Fig. S10. NMJ dismantling during *Drosophila* metamorphosis is similar in controls and *orion* mutants. A-L, Composite confocal z-stack images of muscle 4 NMJ in wild type larvae (L3) **(A-F)** and 6 h APF **(G-L)**, labeled with anti-DLG antibody (green) and anti-HRP antibody (red). At L3, the anti-DLG staining surrounds the bouton membranes as a thin layer in controls **(A-C)** and *orion* mutants **(D-F)**. **(G-L)** At 6 h APF the NMJ is similarly disorganized in both, controls **(G-I)** and *orion* mutants **(J-L)**, a high number of filopodia (arrow in H and K) and HRP-labelled debris (arrowhead in K, L) were observed, the anti-DLG staining became discontinuous and fragmented. Individual synaptic boutons are no longer observed with the HRP-staining. The motor neuron became shorter and swollen at the synapse. Genotypes are listed in Supplementary list of fly strains. Scale bar is 20 µm. n = 31 and 38 for wild type and $orion^{1}$ mutants respectively. Replicated at least three times.

Supplementary Materials and Methods

List of Fly Strains:

Fig. 1.

(A) alrm-GAL4-GFP: y w^{67c23} / Y; alrm-GAL4 2x UAS-mCD8-GFP/+
(B) orion$^{\Delta C}$; alrm-GAL4-GFP: w^{67c23} orion$^{\Delta C}$ / Y; alrm-GAL4 2x UAS-mCD8-GFP/+
(C, E) control: y w^{67c23} sn^3 FRT19A / Y or y w^{67c23} sn^3 FRT19A / y w^{67c23} sn^3 FRT19A
(D, F) orion^1: y w^{67c23} sn^3 orion ^{1} FRT19A / Y or y w^{67c23} sn^3 orion ^{1} FRT19A / y w^{67c23} sn^3 orion^1 FRT19A

Fig. 2.

(A, F, H) alrm-GAL4-GFP: y w^{67c23} / Y; alrm-GAL4 2x UAS-mCD8-GFP/+
(B) orion$^{\Delta C}$; alrm-GAL4-GFP: w^{67c23} orion$^{\Delta C}$ / Y; alrm-GAL4 2x UAS-mCD8-GFP/+
(C) control: y w^{67c23} / Y; alrm-GAL4 2x UAS-mCD8-GFP/+; **orion$^{\Delta C}$:** w^{67c23} orion$^{\Delta C}$ / Y; alrm- GAL4 2x UAS-mCD8-GFP/+
(D) alrm-GAL4-GFP: w* / Y; alrm-GAL4 UAS-mCD8-GFP hs-repo-FLP $^{6.2}$; FRT2A/FRT2A, TubGAL80
(E) orion$^{\Delta C}$; alrm-GAL4-GFP: w^{67c23} orion$^{\Delta C}$ / Y; alrm-GAL4 UAS-mCD8-GFP hs-repo-FLP $^{6.2}$; FRT2A/FRT2A, TubGAL80
(G, I) orion^1; alrm-GAL4-GFP: y w^{67c23} sn^3 orion ^{1} FRT19A / Y; alrm-GAL4 2x UAS-mCD8GFP

Fig. 3.

(A-D) control : y w^{67c23} /Y or y w^{67c23} / y w^{67c23}
(E-H) orion^1 : y w^{67c23} sn^3 orion ^{1} FRT19A / Y or y w^{67c23} sn^3 orion ^{1} FRT19A/ y w^{67c23} sn^3 orion ^{1} FRT19A
(I, J) control: y w^{67c23} /Y or y w^{67c23} / y w^{67c23}, **orion^1:** y w^{67c23} sn^3 orion ^{1} FRT19A / Y or y w^{67c23} sn^3 orion ^{1} FRT19A/ y w^{67c23} sn^3 orion ^{1} FRT19A
(K) elav-GAL4; UAS-orion: elav-GAL4 w UAS-CD8-GFP hs-FLP / Y or elav-GAL4 w UAS- CD8-GFP hs-FLP / y w^*; UAS-orion-B-myc / +
(L) orion$^{\Delta C}$ elav-GAL4 : elav-GAL4 w^* UAS-CD8-GFP orion$^{\Delta C}$ / Y
(M) orion$^{\Delta C}$ elav-GAL4 ; UAS-orion: elav-GAL4 w^* UAS-mCD8-GFP orion$^{\Delta C}$; UAS-orion- B-myc / +
(N, O) orion$^{\Delta C}$ elav-GAL4 ; UAS-orion : elav-GAL4 w^* UAS-mCD8-GFP orion$^{\Delta C}$ / Y ; UAS- orion-B-myc / +, **elav-GAL4; UAS-orion:** elav-GAL4 w UAS-CD8-GFP hs-FLP / Y or elav- GAL4 w UAS-mCD8-GFP hs-FLP / y w^*; UAS-orion-B-myc / +, **orion$^{\Delta C}$, elav-GAL4 :** elav- GAL4 w^* UAS-mCD8-GFP orion$^{\Delta C}$ / Y
(P, Q, R) Crz-GAL4 : yw^{67c23}/Y ; Crz-GAL4 / +, **orion $^{\Delta C}$; UAS-orion :** w^{67c23} orion$^{\Delta C}$ / Y ; UAS-orion-B-myc / +, **orion $^{\Delta C}$; UAS-orion ; Crz-GAL4 :** w^{67c23} orion$^{\Delta C}$ / Y ; UAS-orion-B- myc / Crz-GAL4 ; **UAS-RNAi-Orion :** w^{67c23}/Y **or** yw^{67c23}/ w^*, UAS-orion-RNAi/+ ;**UAS- RNAi-Orion ; Crz-GAL4 :** w^*/Y, UAS-orion-RNAi/+ ; Crz-GAL4 /+
elav-GAL4 : elav-GAL4 w UAS-CD8-GFP hs-FLP / Y or elav-GAL4 w UAS-CD8-GFP hs-FLP / y w^* ;
elav-GAL4 ; UAS-RNAi-Orion : elav-GAL4 w UAS-CD8-GFP hs-FLP / Y or or elav- GAL4 w UAS-CD8-GFP hs-FLP / y w^*; UAS-orion-RNAi/+

Fig. 4.

(A, B) ***repo-GAL4-GFP :*** *y w^{67c23} sn^3 FRT19A / Y ; repo-GAL4 UAS-mCD8-GFP/+*
(C) control : *y w^{67c23} sn^3 FRT19A / Y ; repo-GAL4 UAS-mCD8-GFP/+ ,* ***orion^1:*** *y w^{67c23} sn^3 orion ^{1} FRT19A/ Y; repo-GAL4 UAS-mCD8-GFP/+*
(D-E) ***alrm-GAL4-GFP:*** *y w^{67c23} / Y* or *yw^{67c23} / yw^{67c23}; alrm-GAL4 2x UAS-mCD8-GFP / Tb* **(G, G')** ***NP2222-GAL4-GFP:*** *w^* / Y*; or *w * / y w^{67c23}; NP2222-GAL4 / +; 2x UAS-mCD8- GFP/+*
(H, H', I, I') ***UAS-Rab7-GFP; repo-GAL4:*** *w^* / Y*; or *w * / y w^{67c23}; UAS-Rab7-GFP/+; repo- GAL4 / +*
(J, J') ***orion$^{\Delta C}$; UAS-Rab7-GFP; repo-GAL4:*** *w^{67c23} orion$^{\Delta C}$ / Y; UAS-Rab7-GFP/+; repo- GAL4 /+*
(K, L) ***control:*** *yw / Y*; or *w * / yw; UAS-Rab7-GFP/+; repo-GAL4 /+;* ***orion$^{\Delta C}$:*** *w^{67c23} orion$^{\Delta C}$ / Y; UAS-Rab7-GFP/+; repo-GAL4 /+*

Fig. 5.

(A, E, I) ***PDF-GAL4-GFP:*** *y w^{67c23} sn^3 FRT19A / Y; Pdf-GAL4 / 2 x UAS-mCD8-GFP; Pdf-GAL4 / +*
(B, F, J) ***orion^1; PDF-GAL4-GFP:*** *y w^{67c23} sn^3 orion ^{1} FRT19A / Y; Pdf-GAL4 / 2 x UAS- mCD8-GFP; Pdf-GAL4 / +*
(C, D, G, H, K, L) control: *y w^{67c23} sn^3 FRT19A / Y; Pdf-GAL4 / 2 x UAS-mCD8-GFP; Pdf-GAL4 / +,* ***orion^1:*** *y w^{67c23} sn^3 orion ^{1} FRT19A / Y; Pdf-GAL4 / 2 x UAS-mCD8-GFP; Pdf-GAL4 / +*
(M) orion $^{\Delta C}$; UAS-orion : *w^{67c23} orion$^{\Delta C}$ / Y ; UAS-orion-B-myc / +.*
(N) orion$^{\Delta C}$, elav-GAL4 ; UAS-orion : *elav-GAL4 w^* UAS-CD8-GFP orion$^{\Delta C}$ / Y ; UAS- orion-B-myc / +*
(O, P) ***control:*** *y w^{67c23} sn^3 FRT19A / Y; Pdf-GAL4 / 2 x UAS-mCD8-GFP; Pdf-GAL4 / +,* ***orion $^{\Delta C}$; UAS-orion*** **:** *w^{67c23} orion$^{\Delta C}$ / Y ; UAS-orion-B-myc / +,* **elav-GAL4, orion$^{\Delta C}$; UAS-** ***orion*** **:** *elav-GAL4 w^* UAS-CD8-GFP orion$^{\Delta C}$ / Y ; UAS-orion-B-myc / +*

Fig. 6.

(A-F') ***MZ0709-GAL4-GFP:*** *w ^{1118} / Y*; or *w ^{1118} / y w^{67c23}; MZ0709-GAL4 / 2x UAS-mCD8- GFP*
(G-I) ***NP2222-GAL4-GFP:*** *w^{11118} / Y*; or *w^{1118} / y w^{67c23}; NP2222-GAL4 / +; 2x UAS-mCD8- GFP/+*
(J) ***NP2222-GAL4-GFP:*** *y w^{67c23} sn^3 FRT19A / Y; NP2222-GAL4 / 2x UAS-mCD8-GFP* **(K)** ***orion^1; NP2222-GAL4-GFP:*** *y w^{67c23} sn^3 orion ^{1} FRT19A / Y ; 2 x UAS-mCD8-GFP / NP2222-GAL4.* **(L)** ***control*:** *y w^{67c23} sn^3 FRT19A / Y; NP2222-GAL4 / 2x UAS-mCD8-GFP;* **orion^1:** *y w^{67c23} sn^3 orion ^{1} FRT19A / Y ; 2 x UAS-mCD8-GFP / NP2222-GAL4.*

Fig. 7.

(A, C, F) ***Or85e-GAL4-GFP:*** *y w^{67c23} sn^3 FRT19A / Y; 2 x UAS-mCD8-GFP / +; Or85e-GAL4 / +*
(B, D, G) ***orion^1;*** **Or85e-GAL4-GFP:** *y w^{67c23} sn^3 orion ^{1} FRT19A / Y; 2 x UAS-mCD8-GFP / +; Or85e-GAL4 / +*

Development • Supplementary information

Fig. 8.
(A, A') ***wild type*:** *y, w^{67c23}, sn^3, FRT19A / w^-; dpr1–GAL4, 5xUAS–mCD8-GFP, ase-FLP^{2b} / +; FRT2A/tub–GAL80, FRT2A*
(B, B') ***$orion^1$/+*:** *y, w^{67c23}, sn^3, $orion^1$, FRT19A / w^-; dpr1–GAL4, 5xUAS–mCD8-GFP, ase- FLP^{2b} / +; FRT2A/tub–GAL80, FRT2A*
(C, C') ***wild type*:** *y, w^{67c23}, sn^3, FRT19A / Y; dpr1–GAL4, 5xUAS–mCD8-GFP, ase-FLP^{2b} / +; FRT2A/tub–GAL80, FRT2A*
(D, D') ***$orion^1$*:** *y, w^{67c23}, sn^3, $orion^1$, FRT19A / Y; dpr1–GAL4, 5xUAS–mCD8-GFP, ase-FLP^{2b} / +; FRT2A/tub–GAL80, FRT2A*

Fig. S1.
(A-E) ***Crz-GAL4-GFP :*** *y w^{67c23} sn^3 ^{1} FRT19A / Y; + / 2 x UAS-mCD8-GFP; Crz-GAL4 / +* **(F-J)** ***$orion^1$; Crz-GAL4-GFP:*** *y w^{67c23} sn^3 orion ^{1} FRT19A / Y; + / 2 x UAS-mCD8-GFP; Crz- GAL4 / +*
(K-N) ***control:*** *y w^{67c23} sn^3 ^{1} FRT19A / Y; + / 2 x UAS-mCD8-GFP; Crz-GAL4 / + ;* ***$orion^1$: :*** *y w^{67c23} sn^3 orion ^{1} FRT19A / Y; + / 2 x UAS-mCD8-GFP; Crz-GAL4 / +,* ***yw:*** *y w^{67c23} ,* ***$orion^{\Delta C}$*:** *w^{67c23} $orion^{\Delta C}$*

Fig. S2.
(A, C, E) ***repo-GAL4-GFP*:** *y w^{67c23} / Y* or *y w^{67c23} / y w^{67c23}; repo-GAL4 UAS-mCD8-GFP/+* **(B, D, F)** ***$orion^{\Delta C}$; repo-GAL4-GFP*:** *w^{67c23} $orion^{\Delta C}$ / Y; repo-GAL4 UAS-mCD8-GFP/+.*

Fig. S3.
(A-B'') ***MZ0709-GAL4-GFP:*** *w^{1118} / Y*; or *w ^{1118} / y w^{67c23}; MZ0709-GAL4 / 2x UAS-mCD8- GFP*

Fig. S4.
(A, C) ***UAS-Casor; Crz-GAL4:*** *+ / UAS-Casor; Crz-GAL4 / +*
(B, D) ***$orion^{\Delta C}$; UAS-Casor; Crz-GAL4:*** *w^{67c23} $orion^{\Delta C}$/Y; + / UAS-Casor; Crz-GAL4 / +* **(E)** ***control:*** *+ / UAS-Casor; Crz-GAL4 / +* ***$orion^{\Delta C}$:*** *w^{67c23} $orion^{\Delta C}$/Y; + / UAS-Casor; Crz- GAL4 / +*

Fig. S5.

(A, C) ***PDF-GAL4-GFP:*** *y w^{67c23} sn^3 FRT19A / Y; Pdf-GAL4 / 2 x UAS-mCD8-GFP; Pdf-GAL4 / +*
(B, D) ***$orion^1$; PDF-GAL4-GFP:*** *y w^{67c23} sn^3 orion ^{1} FRT19A / Y; Pdf-GAL4 / 2 x UAS- mCD8-GFP; Pdf-GAL4 / +*
(E) ***control:*** *y w^{67c23} sn^3 FRT19A / Y; Pdf-GAL4 / 2 x UAS-mCD8-GFP; Pdf-GAL4 / +,* ***$orion^1$:*** *y w^{67c23} sn^3 orion ^{1} FRT19A / Y; Pdf-GAL4 / 2 x UAS-mCD8-GFP; Pdf-GAL4 / +*
(F-H) ***PDF-GAL4-GFP:*** *y w^{67c23}/Y or w^* / y w^{67c23}; Pdf-GAL4 / +; Pdf-GAL4 /+;* ***$orion^{\Delta C}$:*** *w^{67c23} $orion^{\Delta C}$/Y* ; or *w^{67c23} $orion^{\Delta C}$/ w^{67c23} $orion^{\Delta C}$*; **$orion^{\Delta C}$; UAS-orion ; PDF-GAL4:** *w^{67c23} $orion^{\Delta C}$/Y; Pdf-GAL4 / +; Pdf-GAL4 / UAS-orion-B-myc;* **UAS-RNAi Orion:** *w^{67c23}/Y* ***or*** *yw^{67c23}/ w^*; UAS-orion-RNAi/+;***PDF-GAL4, UAS-RNAi Orion:** *y w^{67c23}/Y or w^* / y w^{67c23}; Pdf-GAL4 / UAS-orion-RNAi; Pdf-GAL4 /+;* **elav-GAL4:** *elav-GAL4 w UAS-CD8-GFP hs-*

FLP / Y or elav-GAL4 w UAS-CD8-GFP hs-FLP / y w**;* **elav-GAL4; UAS-RNAi Orion:** *elav- GAL4 w UAS-CD8-GFP hs-FLP / Y or elav-GAL4 w UAS-CD8-GFP hs-FLP / w***; UAS-orion- RNAi/+*

Fig. S6.
(A-F') *alrm-GAL4-GFP:* *yw*^{67c23} */ Y* or *yw*^{67c23} */ yw*^{67c23}; *alrm-GAL4 2x UAS-mCD8-GFP/ alrm-GAL4 2x UAS-mCD8-GFP*

Fig. S7.
(A, A') *MZ0709-GAL4-GFP:* *y w*^{67c23} *sn*^{3} *FRT19A / Y*; or *w* ^{1118} */ y w*^{67c23} *sn*^{3} *FRT19A; MZ0709-GAL4 / 2x UAS-mCD8-GFP*
(B, B') *orion*^{1}*; MZ0709-GAL4-GFP*: *y w*^{67c23} *sn*^{3} *orion* ^{1} *FRT19A / Y*; *MZ0709-GAL4 / 2x UAS- mCD8-GFP/+*

Fig. S8.

(A, C, E) *repo-GAL4-GFP:* *y w*^{67c23} */ Y* or *y w*^{67c23} */ y w*^{67c23}*; repo-GAL4 UAS-mCD8-GFP/+* **(B, D, F) *orion*$^{\Delta C}$*; repo-GAL4-GFP:*** *w*^{67c23} *orion*$^{\Delta C}$ */ Y; repo-GAL4 UAS-mCD8-GFP/+*

Fig. S9.

(A, C) *UAS-Casor; PDF-GAL4:* *yw* / Y; Pdf-GAL4 / UAS-Casor; Pdf-GAL4 / +*
(B, D*) orion*$^{\Delta C}$*; UAS-Casor; PDF-GAL4:* *w*^{67c23} *orion*$^{\Delta C}$*/ Y; Pdf-GAL4 / UAS-Casor; Pdf-GAL4 / +*
(E) *control:* *yw* / Y; Pdf-GAL4 / UAS-Casor; Pdf-GAL4 / +* ***orion*$^{\Delta C}$*:*** *w*^{67c23} *orion*$^{\Delta C}$*; Pdf-GAL4 / UAS-Casor; Pdf-GAL4 / +*

Fig. S10.

(A-C, G-I) *control*: *y w*^{67c23} *sn*^{3} *FRT19A /* Y or *y w*^{67c23} *sn*^{3} *FRT19A / y w*^{67c23} *sn*^{3} *FRT19A* **(D-F, J-L) *orion*^{1}*:*** *y w*^{67c23} *sn*^{3} *orion* ^{1} *FRT19A /* Y or *y w*^{67c23} *sn*^{3} *orion* ^{1} *FRT19A / y w*^{67c23} *sn*^{3} *orion*^{1} *FRT19A*

Development • Supplementary information

RAW DATA

Figure 1												
	Graph G				Graph H				Graph I			
	control abd	orion1 abd	control thor	orion1 thor	control abd	orion1 abd	control thor	orion1 thor	control abd	orion1 abd	control thor	orion1 thor
	21,8961667	41,3083333	34,63975	41,57175	436,762	1532,631	177,50075	788,52425	34,7127	65,8867667	9,11845	52,22035
	17,5778	33,9541	24,8023	66,42735	755,651767	1028,94613	208,9021	695,13205	14,0028667	28,6827333	9,46395	11,0629
	19,2931333	75,8267667	24,0178	66,42735	542,454933	1283,26903	392,81585	862,04545	39,2135667	178,057067	10,2324	147,42415
	19,4985667	44,0330333	35,1334	81,8301	587,9113	1029,73243	426,2298	820,27685	15,5698	114,0627	15,53105	97,88895
	25,9722333	37,0714	50,4024	58,0487	735,759833	822,785467	295,032	756,8893	19,0366333	138,693633	22,47855	47,66205
	84,3345667	18,9020333	78,4689	46,0574	657,353267	1334,2379	314,60935	1150,94465	44,3616333	65,9402667	21,51805	88,29885
	18,6113667	53,1314	27,9952	28,6487	501,249933	2234,63947	452,7041	1280,6598	37,7060667	32,9073333	29,9859	45,9835
	37,3486167	60,3272	41,57825	51,11165	332,412767	939,2958	303,28885	1012,2353	8,91606667		18,1789	
	29,2266	15,0428	52,8566	57,6917	552,419233		272,2224		46,2096667		43,46825	
	36,9074667	79,5422667	53,54105	15,88605	416,403333		240,14825		36,6116667		31,31175	
	32,2965	19,2487333	58,80975	86,8146					30,4459		27,7654	
	57,2237667	30,5724	94,5331	22,8679					65,9808		29,9148	
	66,1134	59,0249333	104,08265	32,16635					61,7733333		25,0725	
	22,1913	16,6603333	20,12855	19,34275								
	78,3604667	23,1413333	107,6848	29,71675								
	15,5778667	49,1932667	16,8752	29,71675								
	24,8654	24,7690667	32,0604	26,62065								
	19,8748333	74,8542333	30,9585	90,8719								
	25,9555333		26,06445									

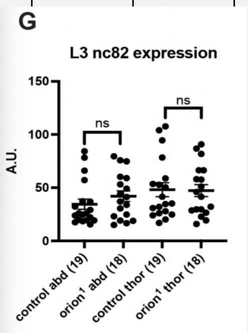

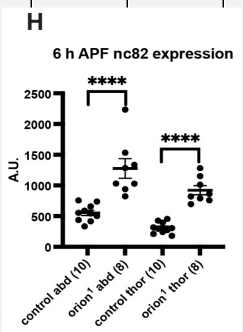

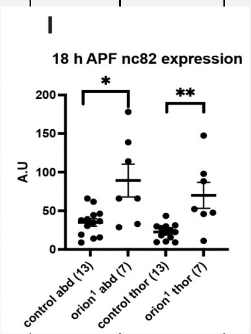

	Graph G				Graph H				Graph I			
MEAN	34,37503071	42,03353518	48,13858158	47,32324444	551,8378366	1275,692153	308,345345	920,8384563	34,9646692	89,1757857	22,61845769	70,07725
n	19	18	19	18	10	8	19	8	13	7	13	7
Number of animal	19	18	19	18	10	8	19	8	13	7	13	7
Number of experiments included in the graph	1	1	1	1	1	1	1	1	1	1	1	1
Number of expriments realised	4	4	4	4	3	3	3	3	3	3	3	3
Comment on the experiments not included in the graph.	Similar results were obtained for the unincluded experiments. Experiments realised in parallel are presented.				Similar results were obtained for the unincluded experiments. Experiments realised in parallel are presented.				Similar results were obtained for the unincluded experiments. Experiments realised in parallel are presented.			

Table S1. Raw data for Fig. 1 graphs

Development • Supplementary information

Development: doi:10.1242/dev.201633: Supplementary information

	Figure 2	
	Graph C	
	control	*Orion∆C*
	349	27
	324	37
	275	28
	242	41
	231	38
**MEAN**	284,2	34,2
**n**	5	5
**Number of animal**	5	5
Number of experiments included in the graph	1	1
Number of expriments realised	3	3
Comment on the experiments not included in the graph.	Visualization of vesicles in n=3 experiments, quantification in 1.	

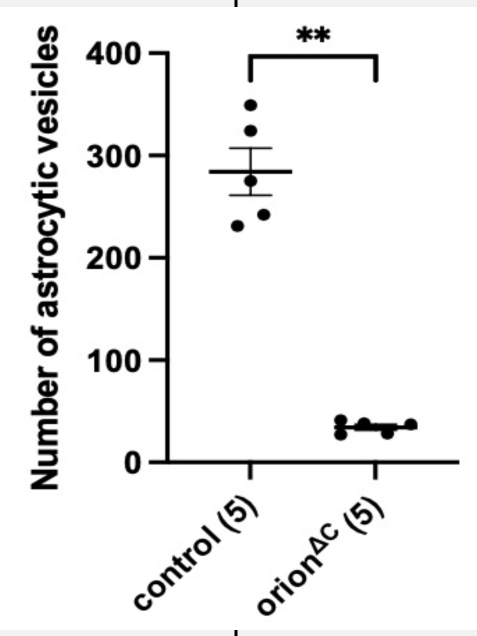

Table S2. Raw data for Fig. 2 graphs

Development • Supplementary information

Figure 3																							
	**Graph I**				**Graph J**				**Graph N**			**Graph O**			**Graph P**			**Graph Q**			**Graph R**		
	control L3	*orion1 L3*	*control 6h APF*	*orion1 6h APF*	*control L3*	*orion1 L3*	*control 6h APF*	*orion1 6h APF*	*Orion∆C ; elav-GAL4 ; UAS Orion*	*Elav-GAL4 ; UAS Orion*	*Orion∆C ; elav-GAL4*	*Orion∆C ; elav-GAL4 ; UAS Orion*	*Elav-GAL4 ; UAS Orion*	*Orion∆C ; elav-GAL4*	*Crz-Gal4*	*Orion∆C ; UAS Orion*	*Orion∆C ; UAS Orion ; Crz-GAL4*	*UAS-RNAi Orion*	*Crz-GAL4*	*UAS-RNAi-Orion ; Crz-GAL4*	*elav-GAL4*	*UAS-RNAi-Orion*	*elav-GAL4 ; UAS-RNAi Orion*
	16	16	3	16	8	8	0	6	1	0	16	0	0	2	8	16	16	0	8	3	0	0	8
	16	16	4	16	8	8	0	5	4	1	15	0	0	2	4	16	16	1	4	8	0	1	6
	15	16	4	16	7	8	1	8	2	1	15	0	0	3	7	16	15	4	7	4	1	4	5
	16	16	6	16	8	8	0	4	2	0	16	0	0	5	3	15	16	3	3	8	1	3	2
	16	16	6	16	8	7	1	6	0	0	16	0	0	6	1	15	16	2	1	14	2	2	2
	16	16	5	16	8	8	0	5	1	0	10	0	0	0	6	16	16	1	6	6	1	1	3
	16	16	0	16	8	8	0	6	4	1	15	0	0		7	15	16	2	7	6	2	2	2
	16	16	3	16	8	7	3	6	1	2	16	0	0	2	10	13	16	2	10	7	1	2	2
	16	16	4	16	8	8	3	6	0	0		0	0		2	14	16	3	2	8	0	3	2
	15	16	5	16	8	8		5		0			0		6	16		1	6	6	3	1	2
	14	16	8	16	8	8		5		1			0		5	16		2	5	8	0	2	5
	16	16		16	8	8		3		6			2		6	16		1	6	1	1	1	10
	16	15		16	8	8		3		0			0		1			2	1	9	5	2	2
	16	14		15	8	8				2			0		10			0	10	5	2	0	7
	16	15		16	8	8				0			0		8			2	8	3	2	2	5
	15	16		3	8	8				0			0		5			6	5	3	2	6	2
	16	16		3	8					1			0		1				1	3	0		
										2			0		3				3	6	1		
										1			0		6				6	5			
										3			1		1				1	2			
															5				5	5			
															2				2	5			
																				6			
																				12			
																				1			
																				4			
																				7			
																				3			
																				4			
**MEAN**	15,70588235	15,76470588	4,363636364	14,41176471	7,941176471	7,875	0,888888889	5,230769231	1,666666667	1,05	14,875	0	0,15	2,857142857	4,86363636	15,3333333	15,88888889	2	4,86363636	5,5862069	1,33333333	2	4,0625
**n**	17	17	11	17	17	16	9	13	9	20	8	9	20	7	22	12	9	16	22	29	18	16	16
**Number of animal**	17	17	11	17	17	16	9	13	9	20	8	9	20	7	22	12	9	16	22	29	18	16	16
Number of experiments included in the graph	2	2	2	2	2	2	2	2	2	2	1	2	2	1	3	2	1	2	3	3	2	2	4
Number of expriments realised	2	2	2	2	2	2	2	2	4	2	3	4	2	3	3	2	3	2	3	3	2	2	4
Comment on the experiments not included in the graph.									Similar results for the 4 experiments.		Similar results for the 3 experiments.	Similar results for the 4 experiments.		Similar results for the 3 experiments.			Similar results for the 3 experiments.						

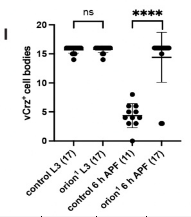

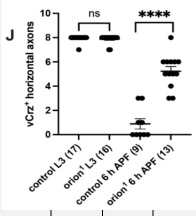

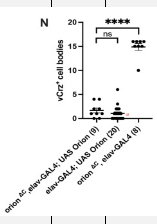

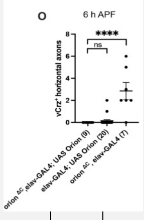

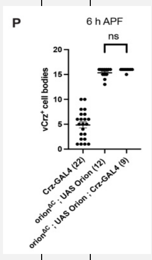

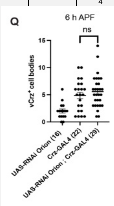

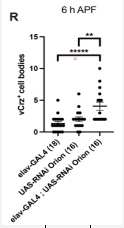

Table S3. Raw data for Fig. 3 graphs

	Figure 4, Graph C			
	Control		Orion1	
	Engulfed	Unengulfed	Engulfed	Unengulfed
	246	36	170	127
n	282		297	
Number of animals	n ≥ 20		n ≥ 20	
Number of experiments included in the graph	4		4	
Number of experiments realised	4		4	
Comments				

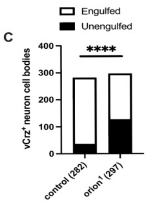

	Figure 4 , Graph K and L									
	2h APF (number of vesicle for each soma)								4h APF (number of vesicle for each soma)	
	control						Orion∆C		control	Orion∆C
	0	0	0	0	0	0	0	0	0	0
	2	1	0	0	0	2	0	0	0	0
	0	0	3	0	0	0	0	0	1	0
	0	0	0	0	0	0	0	0	2	0
	0	1	0	0	1	0	1	0	0	0
	0	0	0	0	0	0	0	0	0	0
	0	0	0	0	2	0	0	0	2	0
	0	0	2	0	0		0	0	1	0
	0	0	0	0	0		0	0	0	0
	0	0	1	0	0		0	0	0	0
	0	1	3	0	0		0	0	0	0
	0	0	0	0	0		0	0	0	0
	0	0	0	0	0		0	0	2	0
	0	0	0	0	0		0	0	0	0
	0	0	0	0	0		0	0	1	0
	0	0	0	0	0		0	0	0	0
	0	0	1	0	0		0		0	0
	2	0	0	0	0		0		0	0
	0	0	0	0	0		0		0	0
	0	0	0	0	3		0		1	0
	0	0	0	0	2		0		0	0
	0	0	1	0	0		0		0	0
	0	0	0	0	1		0		0	0
	0	1	0	0	0		0		0	0
	0	0	0	0	0		0		0	0
	0	0	0	0	0		0		0	0
	0	0	0	0	0		0		0	0
	0	0	0	1	0		0		0	0
	1	0	0	1	0		0		0	0
	0	1	0	0	1		0		0	0
	0	0	0	1	0		0		0	0
	0	0	0	0	0		0		0	0
	0	0	0	0	0		0		0	0
	0	3	0	0	0		0			0
	0	0	0	0	0		0			0
	0	0	0	0	1		0			0
	0	0	0	1	0		0			0
	1	2	0	0	0		0			0
	0	1	0	0	0		0			0
	0	0	0	2	2		0			
	0	1	0	0	0		0			
	0	2	0	0	0		0			
	1	0	0	1	0		0			
	1	0	0	0	0		0			
	0	0	0	0	0		0			
	0	0	0	1	0		0			
	0	0	0	0	0		0			
	0	1	0	0	0		0			
	0	2	0	0	0		0			
	1	0	1	0	0		0			
Number of somas with vesicles	41						1		7	0
Number of somas without vesicles	216						65		26	39
Total number of vesicles	61						1		10	0
TOTAL number of soma	257						66		33	39
n	257						66		33	39
Number of animals	24						6		5	3
MEAN	0,237354086						0,015151515		0,303030303	0
Number of experiments included in the graph	4						2		3	2
Number of experiments realised	4						2		3	2
Comments										Only three animals were analysed for this experiment due to the lethality of the progeny. We made sure the analysed animals were alive thanks to the appearance of the pupae (color, resistance, shape…).

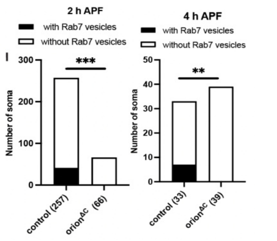

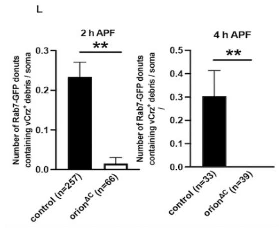

Table S4. Raw data for Fig. 4 graphs

Development • Supplementary information

	Figure 5																	
	Graph C		Graph D		Graph G		Graph H		Graph K		Graph L		Graph O			Graph P		
	Control	*Orion1*	*Control*	*Orion1*	*Control*	*Orion1*	*Control*	*Orion1*	*Control*	*Orion1*	*Control*	*Orion1*	*Control*	*Orion∆C ; UAS Orion*	*Elav-Gal4 Orion∆C ; UAS Orion*	*Control*	*Orion∆C ; UAS Orion*	*elav-GAL4 , Orion∆C ; UAS Orion*
	11347,839	6551,279	2	1	620,407	8978,671	0	1	4224,746	9422,144	0	1	620,407	10356,016	4752,348	0	2	0
	13837,369	10511,786	3	2	6196,652	9142,868	1	4	2011,291	9076,685	0	1	6196,652	9392,982	5002,046	1	2	0
	8368,117	7731,561	1	1	8744,821	12004,113	0	2	4913,721	12812,679	0	3	8744,821	10258,032	1855,852	0	2	0
	12923,424	12044,731	1	2	7997,676	8634,479	0	1	2184,99	12575,581	0	0	7997,676	6117,998	4530,676	0	1	0
	10061,203	10694,581	1	2	1768,831	13462,741	0	3	3174,462	8835,706	0	1	1768,831	8122,775	3805,847	0	2	0
											0	0		7397,253	6753,329		4	0
											0	0		8244,308	6398,646		3	0
											0	0		8271,86	4672,435		2	0
											0	0		7795,063	5291,265		2	0
					Δ						0	0		6181,141	5316,804		2	0
											0	0		7404,163			4	
											0	2		6713,954			2	
											0	2						
												2						
												0						
MEAN	11307,5904	9506,7876	1,6	1,6	5065,6774	10444,5744	0,2	2,2	3301,842	10544,559	0	0,8	5065,6774	8021,295417	4837,9248	0,2	2,333333333	0
n	5	5	5	5	5	5	5	5	5	5	13	15	5	12	10	5	12	10
Number of animal	5	5	5	5	5	5	5	5	5	5	13	15	5	12	10	5	12	10
Number of experiments included in the graph	1	1	1	1	1	1	1	1	1	1	3	3	1	2	1	1	2	1
Number of experiments realised	more than twice	more than twice	more than twice	more than twice	2	2	2	2	3	3	3	3	2	3	3	2	3	3
Comments on the experiments not inculded in the graph.	Similar results were observed with the unincluded experiments.		Similar results were observed with the unincluded experiments.		Similar results. We chose to compare experiments done in parallel.	Similar results. We chose to compare experiments done in parallel.	Similar results. We chose to compare experiments done in parallel.	Similar results. We chose to compare experiments done in parallel.	Similar results. We chose to compare experiments done in parallel.	Similar results. We chose to compare experiments done in parallel.			Similar results.	Similar results.		Similar results.	Similar results.	

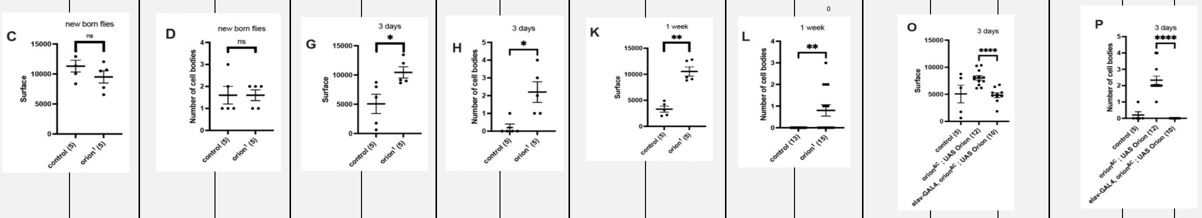

Table S5. Raw data for Fig. 5 graphs

Development • Supplementary information

	Figure 6			
	Graph L			
	CONTROL		MUTANT	
	NP2222-GAL4-GFP		*orion1; NP2222-GAL4-GFP*	
	Engulfed	Unengulfed	Engulfed	Unengulfed
	2	0	0	1
	1	0	3	0
	3	0	0	2
	3	0	2	1
	3	0	2	0
TOTAL	12	0	7	4
MEAN	2,4	0	1,4	0,8
n	12		11	
Number of animal	5		5	
Number of experiments included in the graph	1		1	
Number of experiments realised	1		1	

L

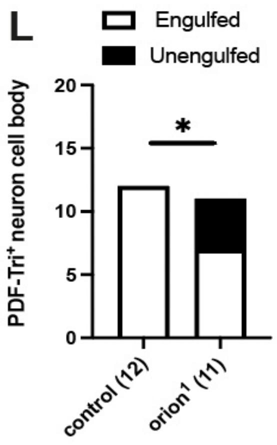

Table S6. Raw data for Fig. 6 graphs

Development • Supplementary information

FIGURE 8

wt female clones:

control

	exp	total ax	
cb	axon	int	frag
10	10	10	0
6	6	6	0
4	4	4	0
6	6	6	0
8	8	8	0
3	3	3	0
6	6	6	0
4	4	4	0
12	12	12	0
9	9	9	0
8	8	8	0
7	7	7	0
7	4	4	0
4	4	4	0
4	4	4	0
4	4	4	0
8	8	8	0
3	3	3	0
10	10	10	0

Number of experiments	n=	average exp	average injured int	average injured frag
3	17	6,3	0,0	0,0

7 dpa

exp		extra ax	
cb	axon	int	frag
2	2	2	2
2	2	2	0
1	1	1	2
0	0	0	2
2	2	2	0
0	0	0	3
3	3	3	1
1	1	1	2
0	0	0	2
1	1	1	1
0	0	0	2
0	0	0	4
1	1	1	0
0	0	0	2
0	0	0	0
0	0	0	2
2,0	2,0	2,0	0,0
2,0	2,0	2,0	2,0
2,0	2,0	2,0	1,0

Number of experiments	n=	average exp	average injured int	average injured frag
3	17	0,9	0,0	1,5

wt male clones

control

	exp	extra ax	
cb	axon	int	frag
5	5	5	0
13	13	13	0
12	12	12	0
15	15	15	0
12	12	12	0
13	13	13	0
13	13	13	0
6	6	6	0
10	10	10	0
13	13	13	0
7	7	7	0
10	10	10	0
12	12	12	0
12	12	12	0
10	10	10	0
10	10	10	0
11	11	11	0
13	13	13	0
7	7	7	0

Number of experiments	n=	average exp	average injured int	average injured frag
3	17	10,8	0,0	0,0

7 dpa

	exp	extra ax	
cb	axon	int	frag
1	1	1	0
1	1	1	1
0	0	0	2
0	0	0	2
0	0	0	3
1	1	1	3
4	4	4	1
1	1	1	2
1	1	1	1
0	0	0	2
1	1	1	0
1	1	1	0
1	1	1	2
2	2	2	1
2	2	2	2
3	3	3	0
1	1	1	2
3	3	3	3
2	2	2	3

Number of experiments	n=	average exp	average injured int	average injured frag
3	17	1,2	0,0	1,4

summary:

		ctl	int	deb
wt female	-	6,3	0,0	0,0
	+	0,9	0,0	1,5
orion female	-	6,1	0,0	0,0
	+	1,4	0,0	1,8
wt male	-	10,8	0,0	0,0
	+	1,2	0,0	1,4
orion male	-	10,2	0,0	0,0
	+	2,2	0,0	2,5

orion female clones:

control

	exp	total ax	
cb	axon	int	frag
9	9	9	0
4	4	4	0
5	5	5	0
5	5	5	0
10	10	10	0
7	7	7	0
10	10	10	0
4	4	4	0
6	6	6	0
4	4	4	0
4	4	4	0
2	2	2	0
9	9	9	0
6	6	6	0
2	2	2	0
7	7	7	0
9	9	9	0
6	6	6	0

Number of experiments	n=	average exp	average injured int	average injured frag
3	17	6,1	0,0	0,0

7 dpa

exp		extra ax	
cb	axon	int	frag
3	3	3	1
0	0	0	4
0	0	0	1
0	0	0	1
2	2	2	2
1	1	1	2
2	2	2	0
0	0	0	2
1	1	1	3
3	3	3	1
1	1	1	1
2	2	2	1
1	1	1	2
1	1	1	3
1	1	1	2
2	2	2	2
3	3	3	2
2	2	2	2

Number of experiments	n=	average exp	average injured int	average injured frag
3	17	1,4	0,0	1,8

orion male clones

control

	exp	extra ax	
cb	axon	int	frag
5	5	5	0
15	15	15	0
10	10	10	0
15	15	15	0
12	12	12	0
5	5	5	0
12	12	12	0
10	10	10	0
8	8	8	0
13	13	13	0
9	9	9	0
6	6	6	0
13	13	13	0
8	8	8	0
10	10	10	0
11	11	11	0
12	12	12	0

Number of experiments	n=	average exp	average injured int	average injured frag
3	17	10,2	0,0	0,0

7 dpa

	exp	extra ax	
cb	axon	int	frag
1	1	1	2
2	2	2	1
3	3	3	3
3	3	3	3
4	4	4	2
3	3	3	2
3	3	3	2
2	2	2	0
1	1	1	3
1	1	1	4
1	1	1	2
4	4	4	3
2	2	2	2
3	3	3	3
2	2	2	3
1	1	1	4
2	2	2	3

Number of experiments	n=	average exp	average injured int	average injured frag
3	17	2,2	0,0	2,5

E

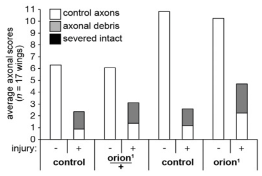

Table S7. Raw data for Fig. 8 graphs

Supplemental Figure 1

Graph K

	control 4h APF	orion1 4h APF	control 6h APF	yw 6h APF	orion1 6h APF	orion⊿C 6h APF
	8	15	8	4	16	15
	11	16	5	7	15	16
	13	16	5	12	16	15
	7	16	6	8	16	16
	10	16	2	6	15	15
	9	16	0	8	16	13
	9	16	2	5	16	16
	6	15	3	2	16	11
	9	15	3	7	16	16
	13	16	3	2	16	16
	11	16	2	7	16	16
	10	16	7	3	11	5
	7	15	1	2	16	15
	5	16	4	1	16	16
	7	16	2	3	16	16
	8	16	0	1	16	16
	9	16		3	16	15
	13	14		2	16	14
	7	16		2	14	14
	7	16		1	16	16
	6	16		8	16	
	5	16		9	16	
	3	2		4	16	
	9	4		5	16	
	9	15		2	16	
	3	16		4	16	
	10	16		5		
	6	16		1	16	
	8	15		5	16	
	1	16		9	16	
	13	16		8	15	
	8	16		7	16	
	9	16		3	16	
	9	16		8	16	
	7	16		0	15	
	13	16		0		
				8		
				7		
				3		
MEAN	8,277777778	15,05555556	3,3125	4,666666667	15,67647059	14,6
n	36	36	16	39	34	20
Number of animal	36	36	16	39	34	20
Number of experiments included in the graph	2	2	2	2	2	2
Number of expriments realised	2	2	2	2	2	2

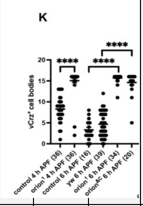

Graph L

	control 4h APF	orion1 4h APF	control 6h APF	yw 6h APF	orion1 6h APF	orion⊿C 6h APF
	8	8	5	3	8	6
	8	8	4	4	7	3
	8	6	4	8	4	3
	6	8	4	4	5	1
	8	8	1	4	6	3
	4	8	0	4	8	5
	4	3	0	3	8	6
	5	8	2	1	8	4
	7	8	3		8	5
	6	8	1	2	6	4
	6	8	2	0	5	4
	6	7	6	2	5	6
	5	8	0	0	5	6
	3	8	4	2	7	5
	4	8	0	2	3	4
	5	8	0	2	8	6
	6	8		1	7	3
	8	8		1	7	4
	5	0		1	4	1
	6	0		2	8	0
	5	8		1	5	
	2	8		4	8	
	2	8		5	6	
	6	8		1	7	
	8	8		3	5	
	2	7		0	7	
	8	8		3		
	6	6		0	6	
	6	8		0	8	
	4	8		0	5	
	8	8		7	8	
	6	8		3	8	
	5			3	8	
	7			1	6	
	4			5	7	
	7			0		
				0		
				5		
				1		
				1		
MEAN	5,666666667	7,15625	2,25	2,28205128	6,5	3,95
n	36	32	16	39	34	20
Number of animal	36	32	16	39	34	20
Number of experiments included in the graph	2	2	2	2	2	2
Number of expriments realised	2	2	2	2	2	2

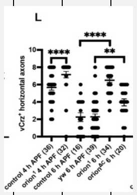

Graph M

	control 4h APF	orion1 4h APF	control 6h APF	yw 6h APF	orion1 6h APF	orion⊿C 6h APF
	2	2	0	0	2	0
	2	2	0	0	2	0
	2	2	0	0	0	0
	2	2	0	0	1	0
	2	1	0	0	1	0
	0	2	0	0	2	0
	0	0	0	0	2	0
	0	2	0	0	2	0
	2	2	0	0	2	0
	2	2	0	0	2	0
	0	2	0	0	2	0
	2	2	0	0	2	0
	0	2	0	0	2	0
	0	2	0	0	2	0
	0	2	0	0	0	0
	1	2	0	0	2	0
	2	2		0	2	0
	2	2		0	2	0
	2	0		0	2	0
	2	0		0	2	0
	1	2		0	1	
	2	2		0	2	
	0	2		0	1	
	2	2		0	2	
	2	2		0	2	
	0	2		0	2	
	2	2		0	2	
	2	2		0	2	
	1	2		0	2	
	0	0		0	2	
	0	0		0	1	
	0			0	2	
	0			0	2	
	2			0	2	
	0			0		
	1			0		
				0		
				0		
				0		
MEAN	1,11111111	1,64516129	0	0	1,73529412	0
n	36	31	16	39	34	20
Number of animal	36	31	16	39	34	20
Number of experiments included in the graph	2	2	2	2	2	2
Number of expriments realised	2	2	2	2	2	2

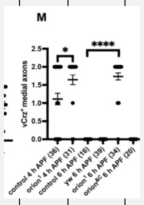

Graph N

	control 4h APF	orion1 4h APF	control 6h APF	yw 6h APF	orion1 6h APF	orion⊿C 6h APF
	0	2	1	0	2	0
	2	2	0	0	1	0
	2	2	0	0	1	0
	2	2	1	0	1	0
	2	2	0	0	1	0
	2	1	0	0	2	0
	2	0	0	0	2	0
	2	2	0	0	2	0
	2	2	1	0	2	0
	2	2	0	0	0	0
	2	2	0	0	1	0
	2	2	1	0	0	1
	2	2	0	0	1	0
	0	1	0	0	2	0
	2	2	1	0	0	0
	2	2	0	0	0	0
	1	2		0	2	0
	2	2		0	2	0
	1	0		0	1	0
	0	0		0	2	0
	2	2		0	1	
	0	2		0	2	
	1	2		0	0	
	1	2		0	2	
	0	2		0	1	
	2	2		0	2	
	2	2		0	2	
	2	2		0	0	
	1	2		0	2	
	0	2		0	2	
	2	0		0	1	
	2			0	2	
	0			0	2	
	2			0	2	
	2			0		
	2			0		
				0		
				0		
				0		
MEAN	1,47222222	1,67741935	0,3125	0	1,35294118	0,05
n	36	31	16	39	34	20
Number of animal	36	31	16	39	34	20
Number of experiments included in the graph	2	2	2	2	2	2
Number of expriments realised	2	2	2	2	2	2

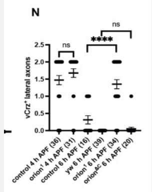

Table S8. Raw data for Fig. S1 graphs

Supplemental Figure 4

	Graph E			
	0h APF		2h APF	
	Control	*Orion⊿C*	*Control*	*Orion⊿C*
nuclei	7	4	79	120
cytoplasm	46	36	0	0
nuclei+cytoplasm	9	8	13	21
n	62	48	92	141
Number of animal	8	5	6	9
Number of experiments included in the graph	2	2	2	2
Number of expriments realised	2	2	2	2

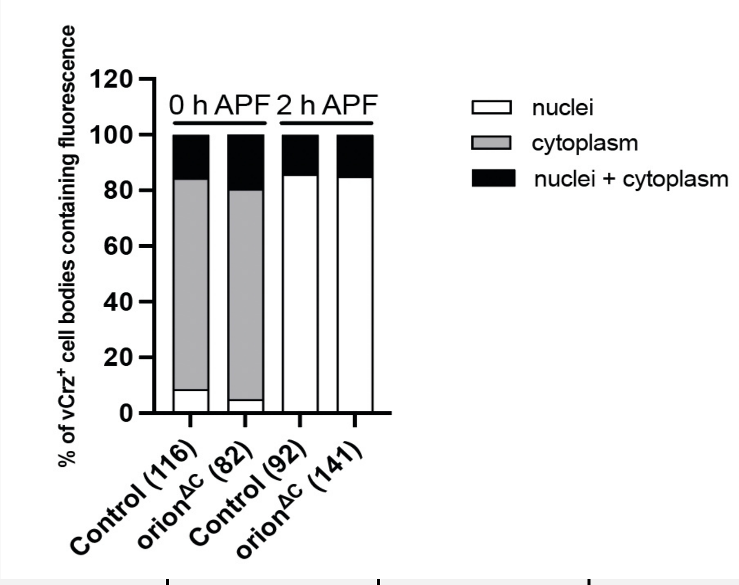

Table S9. Raw data for Fig. S4 graphs

Development • Supplementary information

	Supplemental figure 5										
	Graph E		***Graph F***			***Graph G***			***Graph H***		
	control	*orion1*	*PDF-GAL4*	*Orion∆C*	*Orion∆C ; UAS-Orion ; PDF-GAL4*	*UAS-RNAi-Orion*	*PDF-GAL4*	*PDF-GAL4 ; UAS-RNAi Orion*	*UAS-RNAi Orion*	*elav-GAL4*	*elav-GAL4 ; UAS-RNAi Orion*
	600,062	11931,35	620,407	12133,497	14645,747	3640,668	620,407	3846,414	3640,668	3577,393	8739,813
	418,033	9936,448	6196,652	6443,99	10435,893	4529,672	6196,652	4344,462	4529,672	2904,901	9052,169
	385,361	8858,635	8744,821	11928,7	7949,237	10164,141	8744,821	4687,229	10164,141	6293,905	6627,353
	404,509	12537,994	7997,676	9445,017	11961,662	5347,222	7997,676	5050,777	5347,222	6063,906	8098,313
	564,278	7541,227	1768,831	11439,189	9411,301	8794,479	1768,831	5125,397	8794,479	7190,37	4407,427
				10049,284	12055,355	39,17			39,17	2816,23	8584,998
				7337,627	9549,042	3117,852			3117,852	5984,267	6050,275
				12603,381		8623,468			8623,468	2309,712	4631,399
				8237,304		4893,312			4893,312	1385,344	13248,967
				9484,227		8661,76			8661,76	4443,274	17475,895
				9758,184		4106,979			4106,979	0	7999,598
				11941,736		3684,716			3684,716	3646,054	9639,865
						8041,781			8041,781	3353,144	4714,761
						1616,879			1616,879	4559,483	6670,684
						8910,554			8910,554		2855,975
						6675,419			6675,419		4239,842
						4967,43			4967,43		4112,431
						4833,847			4833,847		7146,321
						474,83			474,83		5613,665
						5557,165			5557,165		3515,123
						3391,99			3391,99		3075,069
						2942,891			2942,891		54,522
						7144,975			7144,975		2846,345
						2123,528			2123,528		5799,33
						8598,485			8598,485		3766,167
						7505,984			7505,984		5949,622
						8047,337			8047,337		3296,268
						3602,008			3602,008		3493,803
						7513,612			7513,612		5229,766
						8722,952			8722,952		
						2660,606			2660,606		
						5937,867			5937,867		
MEAN	474,4486	10161,1308	5065,6774	10066,84467	10858,31957	5464,79934	5065,6774	4610,8558	5464,79934	3894,85593	6101,23331
n	5	5	5	12	7	32	5	5	32	14	29
Number of animal	5	5	5	12	7	32	5	5	32	14	29
Number of experiments included in the graph	1	1	1	2	2	5	1	1	5	2	5
Number of experiments realised	1	1	2	2	2	5	2	3	5	2	5
Comment on the experiments not included in the graph and on single replicates.	Experiment done only one time to check the results we obtained at the previous stage (1 week, Fig 5 I-L).		Similar results with the 2 experiments.				Similar results with the 2 experiments.	Similar results with the 3 experiments.			

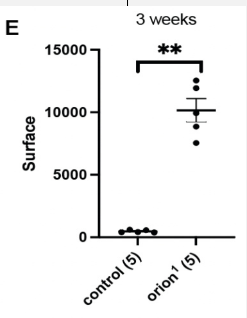

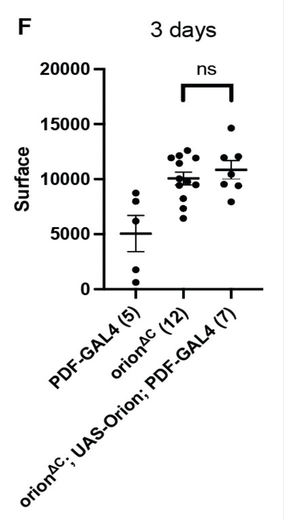

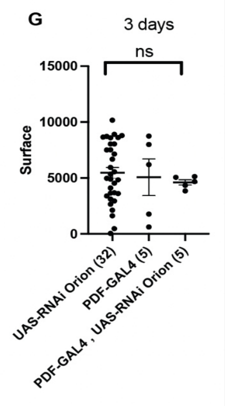

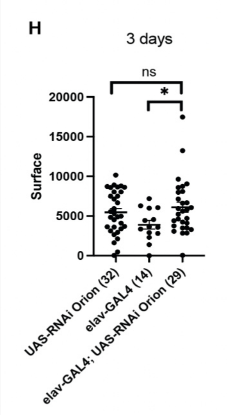

Table S10. Raw data for Fig. S5 graphs

	Supplemental figure 9			
	Graph E			
	Pharate		New born	
	Control	Orion ΔC	Control	Orion ΔC
nuclei	2	2	45	21
cytoplasm	11	13	4	0
nuclei+cytoplasm	1	0	1	0
n	14	15	50	21
Number of animals	7	8	27	12
Number of experiments included in the graph	1	1	3	1
Number of experiments	1	1	3	1

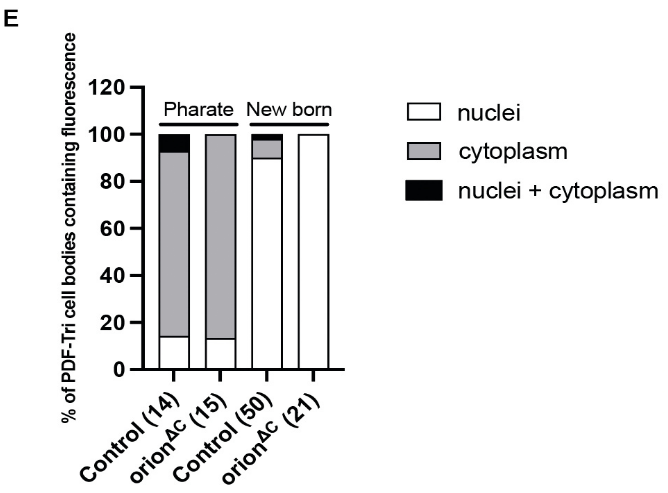

Table S11. Raw data for Fig. S9 graphs
