# Supplementary material for: Chemokine-like Orion is involved in the transformation of glial cells into phagocytes in different developmental neuronal remodeling paradigms
Source: Development. 2023 Oct 2;150(19):dev201633. doi: 10.1242/dev.201633 (PMC10565233; doi:10.1242/dev.201633)
Supplement: Supplementary file 1 [file develop-150-201633_review_history.pdf]

Chemokine-like Orion is involved in the transformation of glial cells into phagocytes in different developmental neuronal remodelling paradigms

Clarisse Perron, Pascal Carme, Arnau Llobet Rosell, Eva Minnaert, Salome Ruiz Demoulin, Heloise Szczkowski, Lukas Jakob Neukomm, Jean-Maurice Dura and Ana Boulanger
DOI: 10.1242/dev.201633

Editor: Steve Wilson

Review timeline
Original submission: 20 January 2023
Editorial decision: 7 March 2023
First revision received: 6 July 2023
Editorial decision: 8 August 2023
Second revision received: 1 September 2023
Accepted: 11 September 2023

Original submission

First decision letter

MS ID#: DEVELOP/2023/201633

MS TITLE: Neuron-secreted chemokine-like Orion is involved in the transformation of glial cells into phagocytes in different neuronal remodeling paradigms

AUTHORS: Clarisse PERRON, Pascal CARME, Arnau Llobet Rosell, Eva Minnaert, Salome Ruiz Demoulin, Heloise Szczkowski, Lukas Jakob Neukomm, Jean-Maurice Dura, and Ana Boulanger

I have now received all the referees' reports on the above manuscript, and have reached a decision. The referees' comments are appended below, or you can access them online: please go to BenchPress and click on the 'Manuscripts with Decisions' queue in the Author Area.

As you will see, there is some variation in the referees' opinions but all express interest in your work. Reviewers 1 and 3 raise some significant criticisms and all reviewers make suggestions to improve your manuscript. I recognise that reviewer 3's report is rather informal in style and could perhaps be clearer on some points, but we think this reviewer (and the other two) raise constructive points that should help improve the manuscript. If you are able to revise the manuscript along the lines suggested, I will be happy receive a revised version of the manuscript. Please also note that Development will normally permit only one round of major revision. If it would be helpful, you are welcome to contact us to discuss your revision in greater detail. Please send us a point-by-point response indicating your plans for addressing the referees' comments, and we will look over this and provide further guidance.

Please attend to all of the reviewers' comments and ensure that you clearly highlight all changes made in the revised manuscript. Please avoid using 'Tracked changes' in Word files as these are lost in PDF conversion. I should be grateful if you would also provide a point-by-point response detailing how you have dealt with the points raised by the reviewers in the 'Response to Reviewers' box. If you do not agree with any of their criticisms or suggestions please explain clearly why this is so.

© 2023. Published by The Company of Biologists under the terms of the Creative Commons Attribution License (https://creativecommons.org/licenses/by/4.0/).

Reviewer 1

Advance summary and potential significance to field

In this paper, Perron et al. seek to build on previous findings on the role of neuronally produced Orion in inducing astrocyte infiltration into neuropils (Boulanger et al., 2021) for clearance of axonal debris during neuronal remodelling in the mushroom body. Here, using different neuronal remodelling paradigms they examine whether Orion independently promotes synapse engulfment and phagocytosis by astrocytes, as well as whether Orion plays a more general role in promoting phagocytosis by glia other than astrocytes. The study is of potential interest, however several data are referred to but not presented in the MS and sample sizes for several experiments are not presented. Ultimately, all of these weaken the manuscript considerably such that not all conclusions are fully supported by the data presented.

Comments for the author

First, the authors focus on the pupal VNC neuropil where astrocytes infiltration into the neuropil occurs before and independently of neuronal apoptosis, thus enabling the investigation of whether astrocyte engulfment and phagocytosis of synapses and axonal debris is dependent on Orion. They authors examined whole animal orion mutants and found that though astrocytic infiltration into the neuropils was unaffected neuronal synapses were not engulfed or phagocytosed. Thus, indicating that Orion is required specifically for astrocytes to engulf and phagocytose neuronal debris.
(See minor comments 1 and 2 below)
The authors then go on to study the role of Orion in transforming other glial sub-types, mainly the cortex and the ensheathing glia, into phagocytes. To do this, they focus on the Vcrz positive neuronal remodelling paradigm. In orion mutants, they nicely show that the Vcrz positive neurons are not cleared up to 18h APF.
Usin-g the elav-Gal4 line, they re-introduce Orion pan-neuronally in an orion mutant, and rescue their phenotype. Based on this rescue they conclude Vcrz+ neurons secrete orion (line 291-292). This is not sufficient evidence to make the claim. The authors are merely showing that expressing Orion pan-neuronally is sufficient to rescue clearance of Vcrz+ neurons.
Although the authors refer to Orion as a Neuron-secreted chemokine-like protein, they have not demonstrated that it is specifically expressed in neurons in these specific contexts and should do so.
Importantly, they should test whether orion expression in Vcrz+ neurons is required for their clearance by knocking Orion down by RNAi under the control of the Crz neuron-specific driver, which they use for other experiments. They should also re-introduce orion specifically in these neurons in an orion mutant and evaluate the phenotype.
The authors suggest that in the absence of neuronally-secreted orion, the glia (specifically the cortex glia) are unable to engulf the Crz neuronal cell bodies and transform into phagocytes. To test this, they first look at glial morphology using the Repo-Gal4 line. They mention that in orion mutants 42% of cell bodies were not in contact with glia. However, they do not provide an image for this, making it impossible to evaluate whether the authors conclusions are supported by the data. Moreover, no clear explanations are provided of how the authors distinguish between Crz neurons that are “in contact” with glia vs. those that are “engulfed” by glia in their analyses.The authors should show representative images for all three cases quantified in Figure 3Q.
Based on their cortex glia specific drivers and orion mutant experiments, the authors concluded that in orion mutants, the cortex glia are unable to engulf and phagocytose the Vcrz positive neurons. The authors need to show the following data to substantiate their argument:
1) Are cortex glia phagocytosing the Vcrz neuronal cell bodies (i.e. do they see any Vcrz positive neuronal debris) in controls?
2) Is the formation of phagolysosomes disrupted in cortex glia in orion mutants (i.e. using lysotracker)?
3) The authors must rule out developmental defects since they are using whole animal mutants. They need to show whether the cell bodies are ever contacted by cortex glia in control and orion mutants at earlier time points prior to neuronal apoptosis to know when the problem arises in the mutant.
Next, using the PDF-Tri neuronal remodelling paradigm, they show that orion is required for PDF neuronal clearance. They state that this is because orion mutant neurons are unable to signal to the cortex glia surrounding their cell bodies to phagocytose its neuronal debris after apoptosis.

© 2023. Published by The Company of Biologists under the terms of the Creative Commons Attribution License (https://creativecommons.org/licenses/by/4.0/).

Many of the points raised above apply here with regard to pinpointing the source of Orion, demonstrating that cortex glia do indeed phagocytose PDF-Tri neuron cell bodies in controls (note that Figure 6 G and I, which show cortex glial processes in the vicinity of a PDF-Tri cell body is not evidence of phagocytosis). Again, we suggest using lysotracker to mark the phagolysosomes to show that the cortex glia are actively phagocytosing the neuronal debris. Please also address issues of developmental defects here.
The last two results clearly demonstrate that orion plays no role in regulating the phagocytosis of severed axons by ensheathing glia after maxillary palp ablation or for neuronal debris removal by wrapping glia in the wings and NMJ dismantling.

Minor points:
1) To show that in orion mutants astrocytes are unable to transform into phagocytes, the authors generated astrocytic clones labelled for GFP in whole animal orion mutants. However, line 246 suggests that the astrocytic clones are mutant for Orion in a wildtype background. Please clarify text accordingly.
2) While the authors do state that the reason for using the pupal VNC was because astrocytic glia have already infiltrated it, their concluding statement (line 255-256) suggests that orion regulates astrocytic infiltration in the pupal VNC. The authors appear to be making this statement based on their previous paper (Boulanger et al., 2021) which was in the context of the mushroom body. Please can the authors clarify the text so that it is clear which context they are referring to.

Reviewer 2

Advance summary and potential significance to field

In this manuscript, Perron et al. characterise the impact of the protein Orion, secreted by the neurons, on the glial cells in different models of neuronal remodelling. They first show that Orion is required for astrocytes to phagocytose synaptic debris in the neuropil of pupal nervous system. Orion null mutant still have nc82 synaptic markers at 6hAPF and 18hAPF while the signal is gone in the controls. In addition, the Orion mutant astrocytes lack the vacuolar structures and lysotracker labelling observed in the controls, suggesting their inability to phagocytose neuronal debris. Second, the authors analysed the role of Orion on the phagocytosis of the vCrz neuron cell body in the pupal neuropil. They show that the expression of Orion by the neuron is required to eliminate the cell body of the vCrz neurons at early pupal stage. They determine that Orion acts downstream of neuron apoptosis and that the cortex glia phagocytose the cell body of the vCrz neuron. Third, the authors establish the role of Orion on the phagocytosis PDF-Tri neurons in the young adult fly. They show that Orion mutants fail to eliminate the PDF-Tri neurons in 3 days old adults and that the cortex glia target primarily the cell body and the ensheathing glia the distal debris. At last, the authors show that Orion is not involved in the debris removal induced by severed ORN axon and wing injury nor in the NMJ developmental dismantling.
Overall, the manuscript highlights the role of an essential neuron-derived ligand that capacitates the glia to phagocytose during specific developmental processes.
This is a very exciting and original piece of work that is general interest for the community. The manuscript is clear and well written and the data are convincing.

Comments for the author

Major comment:
Showing where and when is Orion expressed would be highly appreciated. This would also clarify the lack of phenotypes in the wing and at the NMJ. Is it present but not required or simply not expressed?
Minor comments:
Fig1G-I: there is no mention of the quantification of the nc82 puncta in the method section.
Fig2A-C: The criteria defining the phagocytic vesicles in the astrocytes need to be specified in the method sections.
Fig2F,H: the genotypes are missing in the list of fly strains.
Fig3P-Q: the scoring of the Crz neuron engulfment need to be more explicit possibly with figures to illustrate the scoring strategy. Fig3P does not allow a clear distinction of engulfed, in contact and no contact neurons. At last, the panel Q would benefit from a statistical test to evaluate the p-value.

© 2023. Published by The Company of Biologists under the terms of the Creative Commons Attribution License (https://creativecommons.org/licenses/by/4.0/).

Fig4D,E: the panel E should be renamed D’ since it represents the same image.
Method section “Drosophila stocks”: the Orion mutants are not mentioned in the list.
Method section “quantitation of immunolabelling”: it would be easier to have the different methods of quantification presented in their order of apparition in the text (phagocytic vesicle quantification, vCrz cell bodies engulfment…). In addition, the details on vCrz+ neuron engulfment scoring mentioned in the paragraph “microscopy and image processing” should be shifted to the paragraph “quantitation of immunolabelling”.
The lack of Orion leads to clear phenotypes such as the persistence of the nc82 labelling in VNC at 6h APF. How stable are the Orion induced defects? Is there a compensatory phenotype after some time?

Reviewer 3

Advance summary and potential significance to field

In this study, Perron and colleagues extended their previous findings about the role of Orion during MB neuronal remodeling into other paradigms of remodeling and Wallerian degeneration (WD). in their previous work, the authors found that Orion is secreted from MB neurons before they undergo pruning, and Orion recruits/activates/potentiates astrocytic infiltration into the axonal bundle and its engulfment capabilities. In this paper they find that Orion is required also for activating cortex glia, which are required to eliminate the cell bodies of the vCrz and PDF-Tri neurons. Interestingly, Orion is not required for WD, continuing to highlight that developmental remodeling and WD are two distinct processes.

Comments for the author

I like the wide scope of the study and I think it could be an excellent descriptive DEVELOPMENT paper.
However, I think that in many cases the authors over interpret their results, which also led them to come up with a title that is, in my opinion, hyped. The evidence in this study for "transformation of glial cells into phagocytes" is limited and I thus think one should refrain from going there. You have one figure, in which you quantify these astrocytic vesicles (whatever they really are). I will give more examples for overstatements in the specific comments.

Second big issue: The choice of vCrz and PDF-Tri are interesting - vCrz can be followed by antibody staining and PDF-Tri is unique as it undergoes remodeling in adulthood... However, in Drosophila the two best studied systems are the MB and DA sensory neurons. If this was tested in the Han BioRxiv paper (I don't remember as I read it a while ago) then please cite and comment on this. If not - why not test yourself? This would be a super nice expansion of the system - not only to dendrite pruning but also to engulfment by non-glial cells.

Could be super interesting!

Specifics:

1) Fig 1: why use different alleles in A/B and the rest of figure 1? Also - Thor and abd is misleading in the control as I initially thought you were referring to the genes thor and abd... Perhaps add some schematics to make this clearer? In general - figure annotations could (and should) be improved - in MANY cases, it was insufficient for me to read the text and look at the figures without going to the legends. This is not ideal. Fig 4A-C and other cases of entire stack compared to single slices are another example of insufficient annotation.

2) "Suppression of astrocytic transformation was also observed in individual orion mutant astrocyte clones labeled with mCD8-GFP driven by alrm-GAL4, in which vesicular phagocytic structures were not apparent compared to wildtype (Fig. 2 D, E)" - Is this an orion clone within a heterozygous animal or a labeled clone within a mutant background? also, how do you know this is a single cell? Can you show cell bodies? what are the arrows marking? OK - Orion is expecteed to be expressed in neurons, not astrocytes - so it's for visualizing - but read your text and see it's unclear.

© 2023. Published by The Company of Biologists under the terms of the Creative Commons Attribution License (https://creativecommons.org/licenses/by/4.0/).

3) In Fig 2 - "engulfed synaptic debris" - how is this defined? Or is this your interpretation of the data?

4) Fig 3P/Q - I don't understand how they relate and I would like to see examples to the categories quantified in Q.

5) "These results indicate that Orion is a neuronal signal..." - this is indeed a strong weakness of the paper.

You should its SUFFICIENT to express Orion in neurons (all neurons, unfortunatly) but you never, except in the MB - but that is another paper - show that it is REQUIRED in neurons. Ideally this is an experiment to do. Not to say that all is necessary for the revision but you have two examples in which neurites and cell bodies are eliminated by two glial populations - trying to understand the source of Orion, the distance it can diffuse, are interesting and doable in these systems.

6) the introduction of WD is weird and generates repetition. its strange that WD of the ORNs and WD of wing vein-neurons are in separate sections but the NMJ retraction is together with the wing WD - what is the logic??

7) Perhaps the most exciting finding here is the difference between developmental remodeling and WD. But then the NMJ comes and complicates the picture. To be honest - I don't understand the figures and do now know what conclusions can be drawn. What is a positive control, in which retraction is inhibited? In any case -
It is strange that the NMJ is not discussed at all. Could this be a retraction vs degeneration kind of thing?

First revision

Author response to reviewers' comments

Reviewer 1 Advance summary and potential significance to field

Responses to Reviews

Line numbers correspond to the main version of the manuscript (without corrections in red)

Reviewer 1 Advance Summary and Potential Significance to Field:
In this paper, Perron et al. seek to build on previous findings on the role of neuronally produced Orion in inducing astrocyte infiltration into neuropils (Boulanger et al., 2021) for clearance of axonal debris during neuronal remodelling in the mushroom body. Here, using different neuronal remodelling paradigms they examine whether Orion independently promotes synapse engulfment and phagocytosis by astrocytes, as well as whether Orion plays a more general role in promoting phagocytosis by glia other than astrocytes. The study is of potential interest, however several data are referred to but not presented in the MS and sample sizes for several experiments are not presented. Ultimately, all of these weaken the manuscript considerably such that not all conclusions are fully supported by the data presented.

Reviewer 1 Comments for the Author:
First, the authors focus on the pupal VNC neuropil where astrocytes infiltration into the neuropil occurs before and independently of neuronal apoptosis, thus enabling the investigation of whether astrocyte engulfment and phagocytosis of synapses and axonal debris is dependent on Orion. They authors examined whole animal orion mutants and found that though astrocytic infiltration into the neuropils was unaffected, neuronal synapses were not engulfed or phagocytosed. Thus, indicating that Orion is required specifically for astrocytes to engulf and phagocytose neuronal debris. (See minor comments 1 and 2 below)The authors then go on to study the role of Orion in transforming other glial sub-types, mainly the cortex and the ensheathing glia, into phagocytes. To do this, they focus on the Vcrz positive neuronal

© 2023. Published by The Company of Biologists under the terms of the Creative Commons Attribution License (https://creativecommons.org/licenses/by/4.0/).

remodelling paradigm. In orion mutants, they nicely show that the Vcrz positive neurons are not cleared up to 18h APF.
Using the elav-Gal4 line, they re-introduce Orion pan-neuronally in an orion mutant, and rescue their phenotype. Based on this rescue they conclude Vcrz+ neurons secrete orion (line 291-292). This is not sufficient evidence to make the claim. The authors are merely showing that expressing Orion pan-neuronally is sufficient to rescue clearance of Vcrz+ neurons. Although the authors refer to Orion as a Neuron- secreted chemokine-like protein, they have not demonstrated that it is specifically expressed in neurons in these specific contexts and should do so. Importantly, they should test whether orion expression in Vcrz+ neurons is required for their clearance by knocking Orion down by RNAi under the control of the Crz neuron-specific driver, which they use for other experiments. They should also re-introduce orion specifically in these neurons in an orion mutant and evaluate the phenotype.

We thank this reviewer for his suggestions and for the experiments he proposes to clarify if Orion is secreted by the vCrz and PDF-Tri remodeling neurons. We agree that the fact that Orion expression in neurons is sufficient to rescue the remodeling phenotypes of vCrz and PDF-Tri neurons does not imply that Orion is secreted by these neurons and that RNAi experiments are required to answer this issue.
Consequently, we have performed the experiments requested. Our results show that *UAS-orion-RNAi* expression driven by *Crz* and *PDF-Tri-GAL4* does not block pruning. In addition, *UAS-orion* expression driven by *Crz* and *PDF-GAL4* in an *orion* mutant context does not rescue the mutant phenotype at 6 h APF and three days respectively. Interestingly, we observe a statistically significant decrease of vCrz neuron pruning when expressing *UAS-orion-RNAi* driven by *Elav-GAL4*, confirming the fact that Orion is required in neurons. Consequently, we have modified the sentence “These results suggest that vCrz+ neuron-secreted Orion” on line **319** by “These results suggest that neuron-secreted Orion…”. These new experiments are now added in Fig 3. Concerning the PDF-Tri neurons, we observed some effect on pruning when expressing *UAS-orion-RNAi* with *elav-GAL4* compared to *elav-GAL4* alone, however, since the *UAS-orion-RNAi* alone gave some leaking in new born flies, we could not conclude about this experiment. This is now shown in Supplemental Figure 4.
Importantly, in our recent article in collaboration with Chun Han (H. Ji et al PNAS, 2023), we have shown that *orion* is also highly expressed in fat body, epidermal cells, trachea, hemocytes and glia. Since we cannot exclude additional sources of Orion for vCrz and PDF-Tri neuronal remodeling, we have removed “neuron secreted” from the title to avoid misunderstanding. All this is summarized now in a new last paragraph of the discussion.

The authors suggest that in the absence of neuronally-secreted orion, the glia (specifically the cortex glia) are unable to engulf the Crz neuronal cell bodies and transform into phagocytes. To test this, they first look at glial morphology using the Repo-Gal4 line. They mention that in orion mutants 42% of cell bodies were not in contact with glia. However, they do not provide an image for this, making it impossible to evaluate whether the authors conclusions are supported by the data. Moreover, no clear explanations are provided of how the authors distinguish between Crz neurons that are “in contact” with glia vs. those that are “engulfed” by glia in their analyses. The authors should show representative images for all three cases quantified in Figure 3Q.
Done. We show now representative pictures (Figure 4 A-C). In addition to simplify these data, we have now pooled the engulfed and in contact class in one unique class, to compare only engulfed and unengulfed vCrz cell bodies.

Based on their cortex glia specific drivers and orion mutant experiments, the authors concluded that in orion mutants, the cortex glia are unable to engulf and phagocytose the Vcrz positive neurons. The authors need to show the following data to substantiate their argument:

1) Are cortex glia phagocytosing the Vcrz neuronal cell bodies (i.e. do they see any Vcrz positive neuronal debris) in controls?

Done. We have reenforced this issue on the manuscript and we have pointed debris phagocytosed by cortex glia in the corresponding figure (Figure 4 G arrowheads).
Moreover, we share our Imaris 3D images with this reviewer showing Crz and PDF-tri soma derived

© 2023. Published by The Company of Biologists under the terms of the Creative Commons Attribution License (https://creativecommons.org/licenses/by/4.0/).

debris engulfed by cortex glia as well as PDF-Tri axon derived debris engulfed by ensheathing glia in the three spatial plans.

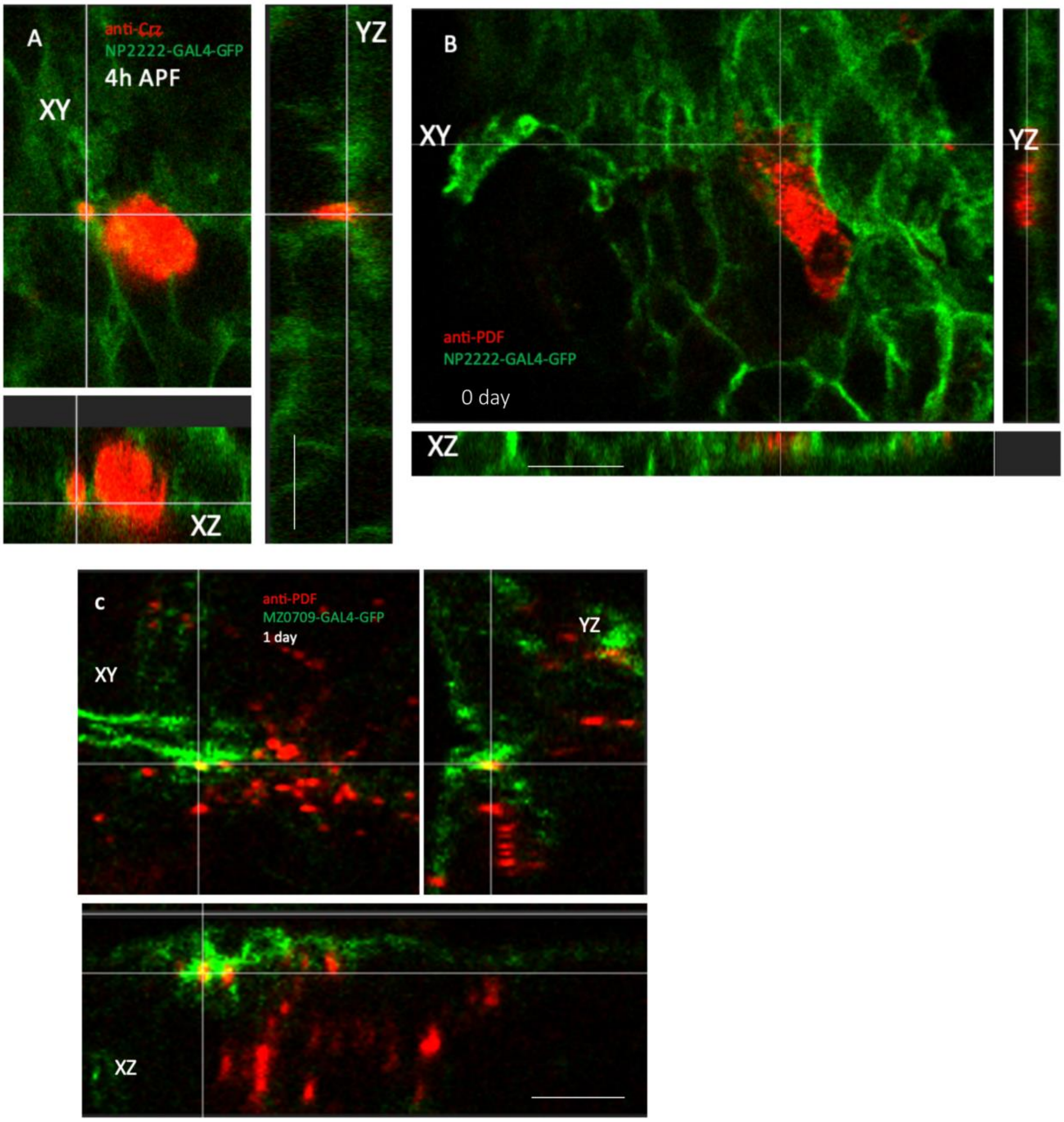

Fig. 1- Confocal 3 D plans (XY, YZ, XZ) showing GFP expression driven by NP2222- GAL4 (**A, B**) and MZ0709-GAL4 (**C**) in green, and anti-Crz expression in VNC at 4 h APF (A) and anti-PDF-Tri expression in new born (B) and one day flies C) in red. The cross shows the region in which debris are being engulfed by cortex (A, B) and ensheathing glia (C) in the three spatial plans (using Imaris software). XY, XZ and YZ are confocal plans in each axis at the cross-hair point indicated ($n \geq 5$). Scale bar is 10μm. Genotypes: *w^{*} / Y*; or *w^{*} / y w^{67c23}; NP2222-GAL4 / +; 2x UAS-mCD8-GFP/+ (A, B), w^{1118} / Y*; or *w^{1118} / y w^{67c23}; MZ0709-GAL4 / 2x UAS-mCD8-GFP (C).*

2) Is the formation of phagolysosomes disrupted in cortex glia in orion mutants (i.e. using lysotracker)?

Unfortunately, we could not use lysotracker in those experiments. In fact, in the experiment performed in figure 2 H, we used GAL4 driven intrinsic GFP without antibody staining and we visualized glia and lysotracker. In this case it was necessary to visualize the Corazonin (or PDF-Tri) neurons (since these neurons are not present all across the VNC like the synapses labeled with

© 2023. Published by The Company of Biologists under the terms of the Creative Commons Attribution License (https://creativecommons.org/licenses/by/4.0/).

nc82) and glia, which required the use of at least one antibody. However, here we encountered technical problems when using the lysotracker followed by antibody staining: *PDF-GAL4; UAS- GFP or Crz-GAL4; UAS-GFP* + anti-Draper required to visualize glia. In order to circumvent this issue, we used an endogenously tagged *Rab7-YFP* allele (*UAS- Rab7-YFP* driven by *Repo-GAL4*) to visualize phagocytic vesicles in glia. Indeed, Rab7 donut-shaped structures have already been described during VNC remodeling as a sign of late phagosome / lysosome presence during phagocytosis. Thus, Rab7 donuts in cortex glia usually form around apoptotic debris and they reflect enhanced expression/localization of Rab7 during corpse engulfment (McLaughlin C.N. et al Dev Cell 2019). Indeed, in controls we observed both diffuse cytoplasmic Rab7 expression and pronounced Rab7 localization in distinct donut-shaped structures.
These donuts are located in cortex glial membranes that surround vCrz$^+$ soma- derived particles at 2 h and 4 h APF and represent a consistent argument in favor of the phagocytic function of Orion-mediated cortex glia phagocytosis during remodeling. The impaired accumulation of Rab7 machinery in *orion* mutant backgrounds supports the idea that phagocytosis in cortex glia is defective. These data are actually included in the manuscript **(Figure 4 H-I).**

3) The authors must rule out developmental defects since they are using whole animal mutants. They need to show whether the cell bodies are ever contacted by cortex glia in control and orion mutants at earlier time points prior to neuronal apoptosis to know when the problem arises in the mutant.
We thank this reviewer for pointing out this question that was indeed not clearly addressed. We understand from this question that this reviewer is asking at which time point the Orion mutant defect (no glial contact) initiates. In fact, cell bodies are always contacted by glia in mutants and controls. Thus, we classified our phenotypes into three different classes: glial engulfment, contact, no contact, based on our observations of confocal plans using constant laser gain levels. Based on that, we observed different levels of GFP+ labelled glial processes. Consequently, the cell bodies for which glial GFP fluorescence was not visualized were considered as no contact. However, it is important to mention that all the Crz$^+$ cell bodies are surrounded by glia, the difference comes from the fact that the cell bodies that are being digested by glial cells display thicker glial processes and consequently higher GFP levels than the one that are not being digested. This is actually explained in lines **193-197**. We have also simplified the corresponding graph and pooled data into two categories Crz soma engulfed (engulfed and touched) and not engulfed (**Fig 4, A-C**), and we have done the same for PDF-Tri soma (**Fig 6, J-L)**. Nevertheless, in order to exclude any developmental effect in *orion* mutant glia, we have also examined Crz neuron-surrounding glia at L3 in control and *orion* mutants and we have not observed any morphological difference in cortex glia. Moreover, we have performed the same experiment for PDF-Tri neuron-surrounding glia at pharate stages and we have observed identical cortex and ensheathing glial morphologies in control and *orion* mutants, which rule out developmental defects in orion mutants.
See below our data, not showed in the manuscript, that we share with this reviewer.

© 2023. Published by The Company of Biologists under the terms of the Creative Commons Attribution License (https://creativecommons.org/licenses/by/4.0/).

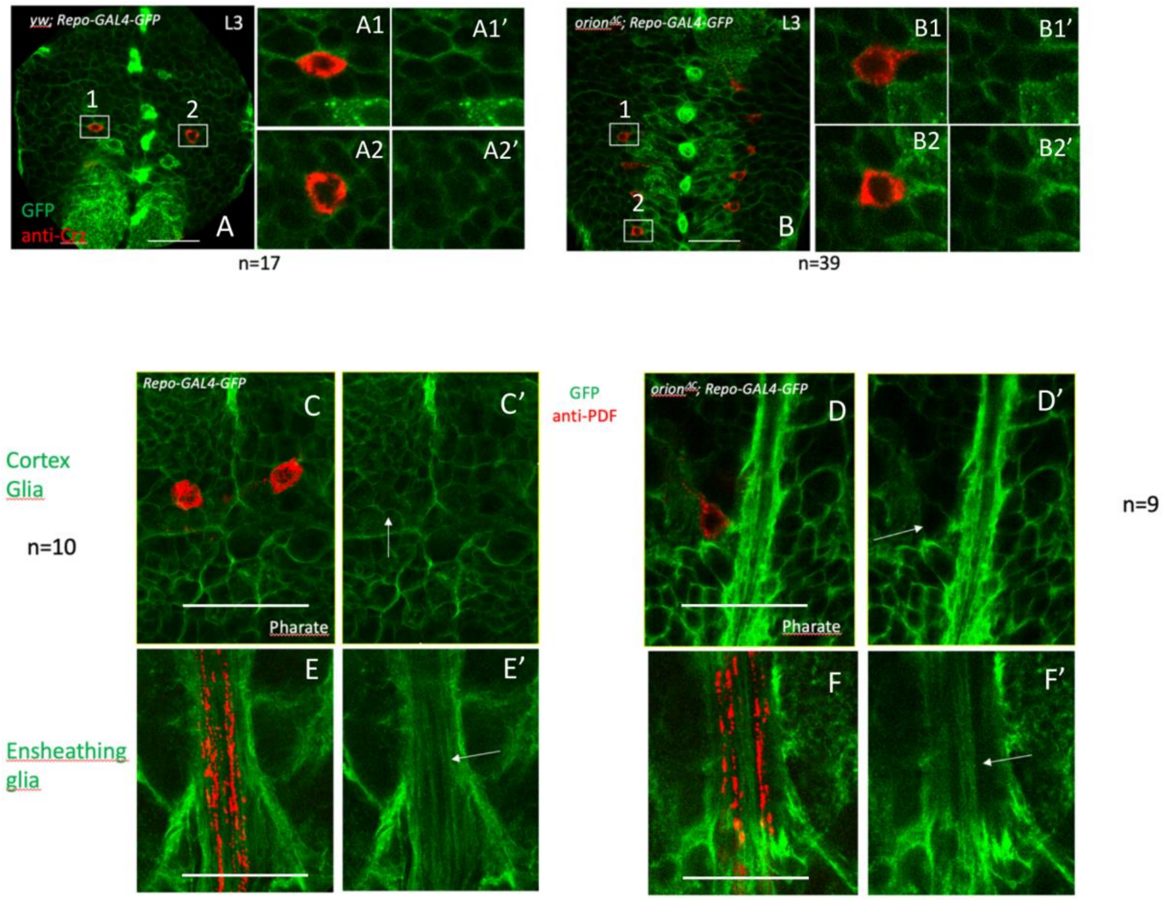

Fig. 2. (A, B) show UAS-GFP expression driven by repo-GAL4 (green) and anti-Crz staining (red) in controls (A) and orion mutants VNC and larval stages (L3). Insets 1, 2 show high magnification of vCrz cell bodies surrounded by cortex glia. Note the similar morphology of the cortex glia in control A1-2 and mutants B1-2. A1', A2', B1' and B2' are single channels of A1, A2, B1, B2 respectively. Scale bars are 30 µm.
Genotypes: *y* w^{67c23} sn^{3} *FRT19A / Y; repo-GAL4 UAS-mCD8-GFP/+* (A, C, E), w^{67c23} $orion^{\Delta C}$ */ Y; repo-GAL4 UAS-mCD8-GFP/+* (B, D, F). **(C-F)** show UAS-GFP expression driven by repo-GAL4 (green) and anti-PDF staining (red) in controls (C, E) and orion mutants (D, F) at pharate stages. Arrow point to cortex glia in C' and D' and ensheathing glia in E' and F'. Note the similar morphology of the cortex and ensheathing glia in control and *orion* mutants in regions close to cell bodies and axons. C', D', E' and F' are single channels of C, D, E and F respectively. Scale bars are 20 µm. Genotypes: *y* w^{67c23} sn^{3} *FRT19A / Y; repo-GAL4 UAS-mCD8-GFP/+* (A, C, E), w^{67c23} $orion^{\Delta C}$ */ Y; repo-GAL4 UAS-mCD8-GFP/+* (B, D, F).

Next, using the PDF-Tri neuronal remodelling paradigm, they show that orion is required for PDF neuronal clearance. They state that this is because orion mutant neurons are unable to signal to the cortex glia surrounding their cell bodies to phagocytose its neuronal debris after apoptosis. Many of the points raised above apply here with regard to pinpointing the source of Orion, demonstrating that cortex glia does indeed phagocytose PDF-Tri neuron cell bodies in controls (note that Figure 6 G and I, which show cortex glial processes in the vicinity of a PDF-Tri cell body is not evidence of phagocytosis). Again, we suggest using lysotracker to mark the phagolysosomes to show that the cortex glia are actively phagocytosing the neuronal debris.
Please see the answer above for the use of lysotracker and new experiments in Figure 6 J-L. Please note that we could not visualize Rab7 donuts in adult brains.

Please also address issues of developmental defects here.
Please see the answer above for both Crz and PDF-Tri neurons (Figure 2 above).

The last two results clearly demonstrate that orion plays no role in regulating the phagocytosis of severed axons by ensheathing glia after maxillary palp ablation or for neuronal debris removal by

© 2023. Published by The Company of Biologists under the terms of the Creative Commons Attribution License (https://creativecommons.org/licenses/by/4.0/).

wrapping glia in the wings and NMJ dismantling.

Minor points:
1) To show that in orion mutants astrocytes are unable to transform into phagocytes, the authors generated astrocytic clones labelled for GFP in whole animal orion mutants. However, line 246 suggests that the astrocytic clones are mutant for Orion in a wildtype background. Please clarify text accordingly.
Done.

2) While the authors do state that the reason for using the pupal VNC was because astrocytic glia have already infiltrated it, their concluding statement (line 255-256) suggests that orion regulates astrocytic infiltration in the pupal VNC. The authors appear to be making this statement based on their previous paper (Boulanger et al., 2021) which was in the context of the mushroom body. Please can the authors clarify the text so that it is clear which context they are referring to.
Done.

Reviewer 2 Advance Summary and Potential Significance to Field:
In this manuscript, Perron et al. characterise the impact of the protein Orion, secreted by the neurons, on the glial cells in different models of neuronal remodelling. They first show that Orion is required for astrocytes to phagocytose synaptic debris in the neuropil of pupal nervous system. Orion null mutant still have nc82 synaptic markers at 6hAPF and 18hAPF while the signal is gone in the controls. In addition, the Orion mutant astrocytes lack the vacuolar structures and lysotracker labelling observed in the controls, suggesting their inability to phagocytose neuronal debris. Second, the authors analysed the role of Orion on the phagocytosis of the vCrz neuron cell body in the pupal neuropil. They show that the expression of Orion by the neuron is required to eliminate the cell body of the vCrz neurons at early pupal stage. They determine that Orion acts downstream of neuron apoptosis and that the cortex glia phagocytose the cell body of the vCrz neuron. Third, the authors establish the role of Orion on the phagocytosis PDF-Tri neurons in the young adult fly. They show that Orion mutants fail to eliminate the PDF-Tri neurons in 3 days old adults and that the cortex glia target primarily the cell body and the ensheathing glia the distal debris. At last, the authors show that Orion is not involved in the debris removal induced by severed ORN axon and wing injury nor in the NMJ developmental dismantling.
Overall, the manuscript highlights the role of an essential neuron-derived ligand that capacitates the glia to phagocytose during specific developmental processes.
This is a very exciting and original piece of work that is general interest for the community. The manuscript is clear and well written and the data are convincing.

Reviewer 2 Comments for the Author:
Major comment:

Showing where and when is Orion expressed would be highly appreciated. This would also clarify the lack of phenotypes in the wing and at the NMJ. Is it present but not required or simply not expressed?
We agree with this reviewer that clarifications about Orion expression are missing in this article and would help to understand the Orion function. We have very recently published our work on Orion expression during development in collaboration with Chun Han (Ji et al, PNAS June 2023). Indeed, Orion is expressed in many tissues and can act at long distance in a non-cell autonomous way via the phosphatidylserine. Thus, expression or absence of Orion in a particular tissue does not determine Orion requirements in this specific tissue. We now referred to the PNAS article in some paragraphs (see lines **563-565, 578-579, 593-596**) and make comments about Orion expression for clarification as suggested. Please see also new results provided to answer first comment of reviewer 1 and comment 5) of reviewer 3 concerning Orion requirements and expression.

Minor comments:
Fig1G-I: there is no mention of the quantification of the nc82 puncta in the method section.
This quantification has been added in the corresponding section of the methods (lines **183-191**).

© 2023. Published by The Company of Biologists under the terms of the Creative Commons Attribution License (https://creativecommons.org/licenses/by/4.0/).

Fig2A-C: The criteria defining the phagocytic vesicles in the astrocytes need to be specified in the method sections.

This is done. See lines **191-193**.

Fig2F,H: the genotypes are missing in the list of fly strains.
These genotypes appear on the first line of Fig 2 genotypes in the supplemental data now, since they are the same as the genotypes used on Fig2 A.

Fig3P-Q: the scoring of the Crz neuron engulfment need to be more explicit possibly with figures to illustrate the scoring strategy. Fig3P does not allow a clear distinction of engulfed, in contact and no contact neurons. At last, the panel Q would benefit from a statistical test to evaluate the p-value.
Done. Illustrative pictures are provided. Scoring reorganization has been performed for Crz neurons to be homogenous with new similar scorings provided for PDF-Tri neurons as requested also by reviewer 1 (see **Fig 4 A-C** and **Fig 6 J-L**).

Fig4D,E: the panel E should be renamed D' since it represents the same image.

Done. These are now G and G' in the new Figure 4.

Method section "Drosophila stocks": the Orion mutants are not mentioned in the list.
Done.

Method section "quantitation of immunolabelling": it would be easier to have the different methods of quantification presented in their order of apparition in the text (phagocytic vesicle quantification, vCrz cell bodies engulfment...). In addition, the details on vCrz+ neuron engulfment scoring mentioned in the paragraph "microscopy and image processing" should be shifted to the paragraph "quantitation of immunolabelling".
This method section has been reorganized as proposed by this reviewer.

The lack of Orion leads to clear phenotypes such as the persistence of the nc82 labelling in VNC at 6h APF. How stable are the Orion induced defects? Is there a compensatory phenotype after some time?
Compensatory phenotypes and persistence depend on the paradigm used. We have now added explanations about *orion* mutant phenotype stability in some of the paradigms used (see lines **258, 305 and 396** for paradigms related to nc82, Crz^+ and PDF-Tri respectively).

Reviewer 3 Advance Summary and Potential Significance to Field:
In this study, Perron and colleagues extended their previous findings about the role of Orion during MB neuronal remodeling into other paradigms of remodeling and Wallerian degeneration (WD). in their previous work, the authors found that Orion is secreted from MB neurons before they undergo pruning, and Orion recruits/activates/potentiates astrocytic infiltration into the axonal bundle and its engulfment capabilities. In this paper they find that Orion is required also for activating cortex glia, which are required to eliminate the cell bodies of the vCrz and PDF-Tri neurons. Interestingly, Orion is not required for WD, continuing to highlight that developmental remodeling and WD are two distinct processes.

Reviewer 3 Comments for the Author:
I like the wide scope of the study and I think it could be an excellent descriptive DEVELOPMENT paper.

However, I think that in many cases the authors over interpret their results, which also led them to come up with a title that is, in my opinion, hyped. The evidence in this study for "transformation of glial cells into phagocytes" is limited and I thus think one should refrain from going there. You have one figure, in which you quantify these astrocytic vesicles (whatever they really are). I will give more examples for overstatements in the specific comments.

We thank this reviewer for this relevant comment. We have now new data showing the

© 2023. Published by The Company of Biologists under the terms of the Creative Commons Attribution License (https://creativecommons.org/licenses/by/4.0/).

transformation of cortex glia into phagocytes. More precisely:

1) we show that both Crz and PDF-Tri soma engulfment is increased in wild type flies (see **Figure 4 A-C** and **Figure 6 J-L**).

2) we produce 3D data showing Crz and PDF-Tri debris inclusions in cortex and ensheathing glia to reviewer 1 (**see Figure 1 below**). Debris are now pointed in **Figure 4 G, G'** and **Figure 6 F, F'** for clarification.

3) we provide data showing Crz^+ debris digestion in Rab7 positive vesicles derived from cortex glia which are not present in *orion* mutants. Please see new **Figure 4 H-I** and comment 2) of reviewer 1.

Second big issue: The choice of vCrz and PDF-Tri are interesting - vCrz can be followed by antibody staining and PDF-Tri is unique as it undergoes remodeling in adulthood... However, in Drosophila the two best studied systems are the MB and DA sensory neurons. If this was tested in the Han BioRxiv paper (I don't remember as I read it a while ago) then please cite and comment on this. If not - why not test yourself? This would be a super nice expansion of the system - not only to dendrite pruning but also to engulfment by non-glial cells. Could be super interesting!

Yes, indeed we collaborate with Chun Han in the BioRxiv article that is actually published in PNAS (Ji et al, PNAS 2023). In this article we show the key role of Orion in da sensory neuron pruning by epithelial cells during development and injury. We cite and comment on this in lines **563-565, 578-579, 593-596** as you suggest.

Specifics:

1) Fig 1: why use different alleles in A/B and the rest of figure 1? Also - Thor and abd is misleading in the control as I initially thought you were referring to the genes thor and abd... Perhaps add some schematics to make this clearer? In general - figure annotations could (and should) be improved - in MANY cases, it was insufficient for me to read the text and look at the figures without going to the legends. This is not ideal. Fig 4A-C and other cases of entire stack compared to single slices are another example of insufficient annotation.
Similar results are obtained with *orion*^{1} and *orion*^{l1C} alleles as described in our previous work in mushroom bodies. This is now explained here for clarification (line **248-249**).
Thoracic and abdominal appear now written in all letters each time we refer to "thor" and "abd". Their meaning is included in lines **185-186**. These regions are now schematized in **Figure 1 C.**
Figure 4 labelling has been modified accordingly. In addition, we have labeled the images in which stacks versus single plans are shown (stack / single plan).

2) "Suppression of astrocytic transformation was also observed in individual orion mutant astrocyte clones labeled with mCD8-GFP driven by alrm-GAL4, in which vesicular phagocytic structures were not apparent compared to wildtype (Fig. 2 D, E)"
- Is this an orion clone within a heterozygous animal or a labeled clone within a mutant background? also, how do you know this is a single cell? Can you show cell bodies? what are the arrows marking? - Orion is expecteed to be expressed in neurons, not astrocytes - so it's for visualizing - but read your text and see it's unclear.

-We have rewritten this sentence to clarify that those are visualization clones in homozygous animals (controls or mutants). Also, we agree with this reviewer that here we have not labeled astrocyte nuclei; therefore, we cannot exclude that the astrocyte clones that we visualize are not indeed clusters of single astrocyte clones. In accordance, we will name them astrocyte clones instead of individual astrocyte clones.
-Arrows point to vesicular structures (phagosomes) that reflect the transformation of astrocytes into phagocytes
-Orion is also expressed in glia (Hui et al, PNAS, 2023) but the input of glia in the Orion function is not yet known.

3) In Fig 2 - "engulfed synaptic debris" - how is this defined? Or is this your interpretation of the data?
Engulfed synaptic debris observed in Fig 2 are now defined in lines **269-270** as nc82 labelled

© 2023. Published by The Company of Biologists under the terms of the Creative Commons Attribution License (https://creativecommons.org/licenses/by/4.0/).

particles delimited by GFP^{+} rings contained in astrocyte processes. They are now pointed by an arrowhead in the inset of Figure 2 F.

4) Fig 3P/Q - I don't understand how they relate and I would like to see examples to the categories quantified in Q.
Done as reviewers 1 and 2 also requested. These are now new pictures in the manuscript: **Figure 4 A-C** and **Figure 6 J-L**.

5) "These results indicate that Orion is a neuronal signal..." - this is indeed a strong weakness of the paper.
You should its SUFFICIENT to express Orion in neurons (all neurons, unfortunatly) but you never, except in the MB - but that is another paper - show that it is REQUIRED in neurons. Ideally this is an experiment to do. Not to say that all is necessary for the revision but you have two examples in which neurites and cell bodies are eliminated by two glial populations - trying to understand the source of Orion, the distance it can diffuse, are interesting and doable in these systems.
Done. We have removed the sentence “Orion is a neuronal signal”. Please see experiments and answers provided to the first paragraph of Reviewer 1 concerning sources and requirements of Orion as well as our recent article (Ji et al PNAS 2023) which shows that *orion* is expressed in many tissues and acts in a non-cell autonomous way.

6) the introduction of WD is weird and generates repetition. its strange that WD of the ORNs and WD of wing vein-neurons are in separate sections but the NMJ retraction is together with the wing WD - what is the logic??
Done, we removed description of WD in ORN as well as the paragraph concerning Drpr as an Orion receptor candidate, both in the discussion part, and placed WD of ORN and wing in the same section.

7) Perhaps the most exciting finding here is the difference between developmental remodeling and WD. But then the NMJ comes and complicates the picture. To be honest - I don't understand the figures and do now know what conclusions can be drawn. What is a positive control, in which retraction is inhibited? In any case
-It is strange that the NMJ is not discussed at all. Could this be a retraction vs degeneration kind of thing?
Yes, indeed NMJ remodeling is a retraction process. This is now clearly enounced and discussed in lines **486-488**.
Positive controls in which retraction is inhibited are described in our previous work (Boulanger et al PlosOne 2012). Indeed, expression of *UAS-EcR*^{DN} either in motor neurons or in post synaptic muscle blocks NMJ retraction and dismantling.

Please see Supplementary Information for formatting and pictures.

<u>Second decision letter</u>

MS ID#: DEVELOP/2023/201633

MS TITLE: Chemokine-like Orion is involved in the transformation of glial cells into phagocytes in different developmental neuronal remodelling paradigms

AUTHORS: Clarisse PERRON, Pascal CARME, Arnau Llobet Rosell, Eva Minnaert, Salome Ruiz Demoulin, Heloise Szczkowski, Lukas Jakob Neukomm, Jean-Maurice Dura, and Ana Boulanger

I have now received all the referees' reports on the above manuscript, and have reached a decision. The referees' comments are appended below, or you can access them online: please go to BenchPress and click on the 'Manuscripts with Decisions' queue in the Author Area.

© 2023. Published by The Company of Biologists under the terms of the Creative Commons Attribution License (https://creativecommons.org/licenses/by/4.0/).

One of the reviewers was unable to provide a detailed review on this version of the manuscript, one is happy with your revisions and one considers that it is essential that you convincingly show the mutant phenotype data (images and not just quantifications) from which you draw your conclusions. I consider this to be an important point to address.

Please attend to all of the reviewers' comments and ensure that you clearly highlight all changes made in the revised manuscript. Please avoid using 'Tracked changes' in Word files as these are lost in PDF conversion. I should be grateful if you would also provide a point-by-point response detailing how you have dealt with the points raised by the reviewers in the 'Response to Reviewers' box. If you do not agree with any of their criticisms or suggestions please explain clearly why this is so.

Reviewer 1

Advance summary and potential significance to field

The authors demonstrate that the chemokine-like factor Orion is required for activating the phagocytic response in astrocytes, cortex and ensheathing glia in some but not all contexts of neuronal debris clearance in Drosophila.

Comments for the author

In the previous version of this manuscript my major concern related to the fact that the authors asserted that specific dying neurons secreted Orion and that this was required to activate the phagocytic response in various glial types (in a context-dependent manner) without evidence to support this conclusion. The authors have performed experiments aimed at pinpointing the neuronal source of Orion and have now tempered their conclusions to reflect that they have not pinpointed the relevant source in this work.

A few minor revisions to the text and figures are still required before publication:

Previously, I raised that the the authors stated that in orion mutants 42% of cell bodies were not in contact with glia but that they did not provide images. They now include a representative image in Figure 4B but it is not convincing. In panel A Crz localisation is nuclear whereas in panel B is it excluded from the nucleus. There is still GFP from glial membranes around the Crz expressing cell in B, it's just that the signal is much weaker. The thicker membranes the authors claim are associated with engulfment isn't validated in this work or elsewhere as far as I can tell. However, the authors do provide much more compelling images in Figure 4 panel G where they show with arrowhead small fragments of Crz+ debris surrounded by glial membranes (much smaller than Crz+ neuronal cell bodies). If the authors can show that these are not observed or observed with lower frequency in orion mutants, this would be very convincing.
Similarly the authors express UAS-Rab7GFP in glia and argue that cortex glia are engulfing neurons because of the the presence of GFP donuts (Figure 4 panel H with arrow). Based on the zoom-in provided, the arrow does not point to a donut but to diffuse GFP stain. Either way for both the experiments contained in panels G and H, representative images are only provided for the control but not for the orion mutants. It is not sufficient to only provide the quantifications. Please provide representative images of the same conditions in iron mutants.
To summarise, for Figure 4, the data is panels A-C are weak and should be removed. Matching representative images of orion mutants for panels G and H should be provided to support the arguments being made.
-minor point: the authors state that they use "an endogenously tagged Rab7 GFP allele" while referring to UAS-Rab7::GFP. This is not an endogenously tagged protein. Please amend the text.
To address whether there are developmental defects the authors examined earlier developmental stages. These images were included in the response to reviewers but were not visible in the pdf. please include these as a supplementary figure in the manuscript and amend the text accordingly.

The same still applies for PDF neurons, only control images are provided in Figure 6 but no images of the phenotype in orion mutants are included with the exception of figure 6 panel K which does show an unfragmented PDF cell body without thick glial membranes around it. Please provide the

© 2023. Published by The Company of Biologists under the terms of the Creative Commons Attribution License (https://creativecommons.org/licenses/by/4.0/).

appropriate data in the figure and also include the data to exclude developmental defects in a supplementary figure and amend the text to include this.

Reviewer 2

Advance summary and potential significance to field

Perron et al. characterise the impact of the protein Orion, secreted by the neurons, on the glial cells in different models of neuronal remodelling.
Overall, the manuscript highlights the role of an essential neuron-derived ligand that capacitates the glia to phagocytose during specific developmental processes.
This is a very exciting and original piece of work that is general interest for the community.

Comments for the author

The authors answered all the comments made by the reviewer(s) and is suitable for publication.

Reviewer 3

Advance summary and potential significance to field

In their revised manuscript the authors have toned down many of what I thought were over-hyped statements.
This would be a nice addition to the field, showing that Orion is required for multiple aspects of developmental remodeling (process elimination AND cell elimination) but not after injury.

Comments for the author

I had minimal time to review this revised version. From a quick read of the point-by-point letter and browsing quickly through the figures, it is my impression that the authors have addressed, or in some cases have attempted to address, all the major concerns that were raised.

Second revision

Author response to reviewers' comments

Answers to reviewer 1:

A few minor revisions to the text and figures are still required before publication:
Previously, I raised that the the authors stated that in orion mutants 42% of cell bodies were not in contact with glia but that they did not provide images. They now include a representative image in Figure 4B but it is not convincing. In panel A Crz localisation is nuclear whereas in panel B is it excluded from the nucleus.
These are typical morphologies of control and mutants at 4 h APF. Indeed, the control image corresponds to a wild type apoptotic soma being “destroyed”, characterized by DNA fragmentation and undistinguishable nuclei, so the distribution of the Crz peptide is observed in the whole soma (see Fig 3 Choi *et al*. *Development* 2006). In the case of the mutant image, the soma remains intact and displays normal morphology with Crz staining in the cytoplasm and obvious unlabeled nuclei.

There is still GFP from glial membranes around the Crz expressing cell in B, it's just that the signal is much weaker.
Yes, indeed the signal is much weaker in mutants because the membrane tethered-GFP signal is a read out of membrane extension and phagocytic cup formation during engulfment. Consequently, in mutants, decreased phagocytosis implies weaker GFP signal.

© 2023. Published by The Company of Biologists under the terms of the Creative Commons Attribution License (https://creativecommons.org/licenses/by/4.0/).

The thicker membranes the authors claim are associated with engulfment isn't validated in this work or elsewhere as far as I can tell. To summarise, for Figure 4, the data is panels A-C are weak and should be removed.
We do not agree with this reviewer concerning the weakness of these data. Our results clearly show active glia surrounding vCrz cell bodies in wild type, versus *orion* mutants, which generates phagocytic cups. Glial membrane expansion has already been proposed and validated as a read-out of phagocytosis in different works: Please see Figs. 6 and 7 *Ziegenfuss et al. Nat Neurosci 2016* from M. Freeman group, showing glial membranes recruitment to degenerating axons; Fig 1 in *Cold Spring harbor laboratory Press, Davidson and Wood: http://cshperspectives.cshlp.org/*, showing GFP-labeled enlarged glial membranes during phagocytic cup formation in an image provided from Kang and Freeman that is quite similar to the one showed in our Fig 4 for cortex glia surrounding Crz-labeled debris; and Fig 3 in *Winfree et al, Cell Death and Disease 2017* from M Logan group showing glial membrane proliferation in axon injured regions. Moreover, enlarged glial membranes creating phagocytic cups are also observed in mice during astrocytic phagocytosis, please see Fig EV4 *Konishi* et al, *EMBO J 2020*. In addition, recent morphologic characterization of phagocytic cups clearly establish that: *"The plasma membrane of the phagocytic cup is extended by the force of branched actin polymerization and, if a target is particularly large, additional membrane from intracellular stores is added to the cup by exocytosis" Barger et al Nature Comm 2019.* Consequently, based on these published data, we consider that our thicker GFP-labeled glial membrane observations are associated with engulfment and phagocytosis. Since the terms glial "membrane extension" and "phagocytic cup" were used in the cited studies to describe glial phagocytosis, we have added this on the text for clarification (lines 192-193, 314-315, 778-779). Thus, we would request these data to be maintained in this manuscript. It is a solid experiment and highly statistic, in which GFP^+ glial expansions around $vCrz^+$ soma have been largely quantified in control and mutants and we consider it is an important information for readers.

However, the authors do provide much more compelling images in Figure 4 panel G where they show with arrowhead small fragments of Crz+ debris surrounded by glial membranes (much smaller than Crz+ neuronal cell bodies). If the authors can show that these are not observed or observed with lower frequency in orion mutants, this would be very convincing. Similarly, the authors express UAS-Rab7GFP in glia and argue that cortex glia are engulfing neurons because of the presence of GFP donuts (Figure 4 panel H with arrow). Based on the zoom-in provided, the arrow does not point to a donut but to diffuse GFP stain. Either way for both the experiments contained in panels G and H, representative images are only provided for the control but not for the orion mutants. It is not sufficient to only provide the quantifications. Please provide representative images of the same conditions in orion mutants. Matching representative images of orion mutants for panels G and H should be provided to support the arguments being made.
Done. We provide new pictures (Figure 4 H-J') and additional quantifications (Figure 4 L) in which we compare wild type and *orion* mutants phagocytic vesicles.

-minor point: the authors state that they use "an endogenously tagged Rab7 GFP allele" while referring to UAS-Rab7::GFP. This is not an endogenously tagged protein. Please amend the text.
Done.

To address whether there are developmental defects the authors examined earlier developmental stages. these images were included in the response to reviewers but were not visible in the pdf. please include these as a supplementary figure in the manuscript and amend the text accordingly.
Done. This is now a new supplementary figure 2.

The same still applies for PDF neurons, only control images are provided in Figure 6 but no images of the phenotype in orion mutants are included with the exception of figure 6 panel K which does show an unfragmented PDF cell body without thick glial membranes around it. Please provide the appropriate data in the figure
We have provided a new supplementary figure 7 including images of the mutant. Please note that adult ensheathing glia enwrap PDF-Tri neuron extensions all along the neuron track and no differences between control and mutant ensheathing glial morphology can be observed. Importantly, since PDF-labelled axons disappear surrounded by ensheathing glia in control, opposite to mutant axons which do not disappear, and due to the fact that lack of Draper in ensheathing glia leads to unpruned axons (*Vita et al Nat Commun* 2021), we have considered our data suggest that Orion is also involved in PDF-Tri neurons and ensheathing glia communication during remodelling.

© 2023. Published by The Company of Biologists under the terms of the Creative Commons Attribution License (https://creativecommons.org/licenses/by/4.0/).

and also include the data to exclude developmental defects in a supplementary figure and amend the text to include this.
Done. This is now a new supplementary figure 8.

Third decision letter

MS ID#: DEVELOP/2023/201633

MS TITLE: Chemokine-like Orion is involved in the transformation of glial cells into phagocytes in different developmental neuronal remodelling paradigms

AUTHORS: Clarisse Perron, Pascal Carme, Arnau Llobet Rosell, Eva Minnaert, Salome Ruiz Demoulin, Heloise Szczkowski, Lukas Jakob Neukomm, Jean-Maurice Dura, and Ana Boulanger
ARTICLE TYPE: Research Article

I am happy to tell you that your manuscript has been accepted for publication in Development, pending our standard ethics checks.

© 2023. Published by The Company of Biologists under the terms of the Creative Commons Attribution License (https://creativecommons.org/licenses/by/4.0/).
